# Bio-Inspired Nanomaterials for Micro/Nanodevices: A New Era in Biomedical Applications

**DOI:** 10.3390/mi14091786

**Published:** 2023-09-18

**Authors:** Mohammad Harun-Ur-Rashid, Israt Jahan, Tahmina Foyez, Abu Bin Imran

**Affiliations:** 1 Department of Chemistry, International University of Business Agriculture and Technology, Dhaka 1230, Bangladesh; mrashid@iubat.edu; 2 Department of Cell Physiology, Graduate School of Medicine, Nagoya University, Nagoya 466-8550, Japan; israt.kyoto@gmail.com; 3 Department of Pharmacy, United International University, Dhaka 1212, Bangladesh; tahmina@pharmacy.uiu.ac.bd; 4 Department of Chemistry, Bangladesh University of Engineering and Technology, Dhaka 1000, Bangladesh

**Keywords:** bio-inspired nanomaterials, micro/nanodevices, biomedical applications, nanotechnology, biomimetic polymers, microfabrication, nano-biotechnology

## Abstract

Exploring bio-inspired nanomaterials (BINMs) and incorporating them into micro/nanodevices represent a significant development in biomedical applications. Nanomaterials, engineered to imitate biological structures and processes, exhibit distinctive attributes such as exceptional biocompatibility, multifunctionality, and unparalleled versatility. The utilization of BINMs demonstrates significant potential in diverse domains of biomedical micro/nanodevices, encompassing biosensors, targeted drug delivery systems, and advanced tissue engineering constructs. This article thoroughly examines the development and distinctive attributes of various BINMs, including those originating from proteins, DNA, and biomimetic polymers. Significant attention is directed toward incorporating these entities into micro/nanodevices and the subsequent biomedical ramifications that arise. This review explores biomimicry’s structure–function correlations. Synthesis mosaics include bioprocesses, biomolecules, and natural structures. These nanomaterials’ interfaces use biomimetic functionalization and geometric adaptations, transforming drug delivery, nanobiosensing, bio-inspired organ-on-chip systems, cancer-on-chip models, wound healing dressing mats, and antimicrobial surfaces. It provides an in-depth analysis of the existing challenges and proposes prospective strategies to improve the efficiency, performance, and reliability of these devices. Furthermore, this study offers a forward-thinking viewpoint highlighting potential avenues for future exploration and advancement. The objective is to effectively utilize and maximize the application of BINMs in the progression of biomedical micro/nanodevices, thereby propelling this rapidly developing field toward its promising future.

## 1. Introduction

Bio-inspired nanomaterials (BINMs), alternatively referred to as biomimetic nanomaterials (BNMs), are a class of materials that are intentionally engineered and manufactured to replicate the intricate structures, functionalities, or mechanisms observed in natural biological systems [1,2,3]. These advancements offer a novel trajectory for the field of material science, facilitating the creation of materials possessing unique characteristics that can effectively tackle a wide range of scientific, technological, and environmental obstacles through successful applications in multidimensional sectors, including medicine and healthcare [4], biotechnology and bioengineering [5], energy [6], environment [7], material science [8], robotics [9,10], and many more [11,12,13]. Various biological entities, including proteins, DNA [14,15], cells [16], and complete organisms [17], can serve as sources of inspiration for these materials. Using DNA’s self-assembling properties has facilitated the construction of shapes and patterns at the nanoscale level [18]. The adhesive characteristics exhibited by gecko feet have served as a source of inspiration for developing sophisticated adhesive materials [19]. Furthermore, the self-cleaning and hydrophobic characteristics exhibited by the lotus leaf have prompted advancements in creating self-cleaning surfaces and coatings with water-repellent properties [20]. The interdisciplinary field of BINMs integrates principles from biology, chemistry, physics, and material science. A bottom-up approach is often employed, commencing at the atomic or molecular level and progressing upward. This is juxtaposed with the conventional top-down methodology, wherein the initial focus is on a larger system that is subsequently deconstructed into smaller constituent parts. Various techniques can be employed to fabricate BINMs, such as molecular self-assembly, in which molecules autonomously organize themselves into desired structures. Another method is biosynthesis, which involves utilizing biological organisms such as bacteria, fungi, or plants to synthesize nanomaterials. The field of BINMs holds significant potential for scientific investigation; however, it is not devoid of inherent obstacles. One of the primary obstacles lies in the capacity to regulate the synthesis and assembly processes of these materials in order to attain the intended properties [21]. There exist additional concerns regarding the potential environmental and health ramifications associated with these nanomaterials, necessitating the need for further examination and comprehensive testing prior to their widespread implementation [22]. Notwithstanding these challenges, BINMs constitute a captivating and burgeoning area of investigation. The advancement of our knowledge in the fields of biology and nanotechnology is expected to enhance the possibilities for the development of novel and influential BINMs.

With the advancement and comprehension of BINMs, an opportunity arises to investigate the pragmatic utilization of these materials in the configuration of micro/nanodevices. Micro/nanodevices, as their nomenclature implies, are miniature devices that operate at the micro- or nanolevel. The significance of micro/nanodevices has increased substantially due to their potential to enhance capabilities in diverse sectors, including medicine, environmental monitoring, electronics, and energy production. These devices provide an unparalleled degree of control and accuracy at a minuscule level, enabling us to devise and develop solutions to previously insurmountable obstacles. The potential for developing novel and influential micro/nanodevices using BINMs is anticipated to grow due to technological advancements and improved comprehension of biological systems. Through the utilization of the distinct characteristics exhibited by these nanomaterials in the form of nanocomposite gels [23,24,25,26] and films [27,28,29], structural colored nanomaterials [30,31,32], organo-metallic nanomaterials [33], molecular machines [34], and nanobiosensors [35] have found widespread application and have replaced mainly more conventional bulk materials in a variety of sectors [36,37,38,39,40,41] as well as in theoretical inquiries [42]. Researchers and practitioners can fabricate devices that imitate or draw inspiration from biological systems to execute targeted functions, frequently surpassing the efficiency and efficacy of conventional devices. Medicine and healthcare are highly significant domains for applying micro/nanodevices [43,44]. Environmental monitoring is a field that extensively utilizes micro/nanodevices [45]. These encompass sensors capable of detecting various environmental pollutants, even in exceedingly low concentrations. These devices can continuously monitor air and water quality, thereby offering significant data that can be utilized to safeguard the environment. The electronics and computing sector represents a significant domain in which micro/nanodevices are widely used [46]. Modern electronic and computing devices rely on various components, such as transistors found in computers and sensors present in smartphones, which collectively serve as the fundamental infrastructure for these technologies. By further reducing the size of these devices and enhancing their operational capabilities, it is possible to develop electronic devices that are more potent and consume less energy. Micro/nanodevices are paramount in energy production and storage [47]. Nanostructured materials have been employed to improve the efficiency of solar cells, fuel cells, and batteries, among other applications. These devices have the potential to enhance energy efficiency, mitigate expenses, and foster the adoption of renewable energy sources. Although the prospect of micro/nanodevices is vast, there are still obstacles to overcome in manufacturing, integration, reliability, and safety. Current investigations in BINMs and their utilization in micro/nanodevices are actively tackling these obstacles, thus laying the foundation for a novel epoch in diverse industries.

The convergence of BINMs and micro/nanodevices is driving a transformative shift across various academic fields, with a particular emphasis on biomedicine. The convergence described in this context capitalizes on the distinctive characteristics of BINMs, which are derived from biological systems, to optimize the functionality of micro/nanodevices. These devices, in turn, provide a pragmatic framework for implementing these nanomaterials. The inherent characteristic of BINMs, known as the “bottom-up” approach, is highly compatible with the micro/nanoscale. This compatibility facilitates the formation of intricate structures through the process of self-assembly. The advantageous collaboration between nanomaterials and biological systems in biomedicine is of great significance, as it allows for the customization of nanomaterials to enhance their interaction capabilities with biological entities. One example of improving targeted drug delivery systems involves the integration of nanomaterials into micro/nanodevices, thereby enabling the accurate administration of drugs to particular cells or tissues. Moreover, developing micro/nanosensors with high sensitivity is feasible, thereby improving the capability for early disease detection and accurate environmental monitoring. Although this interdisciplinary field offers significant prospects, it is important to acknowledge the persistent challenges associated with the control of nanomaterial synthesis and assembly, their integration into devices, and the assurance of safety and efficacy in practical applications. However, the potential advantages signify a promising outlook for this convergence.

**Figure 1:**
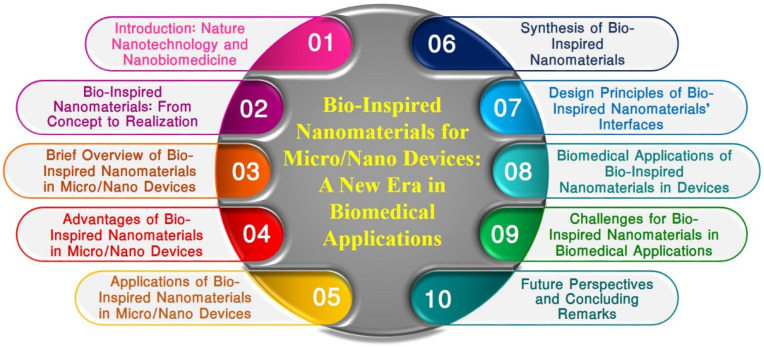
Bio-inspired nanomaterials for micro/nanodevices in biomedical applications.

In this thorough analysis, we set out on a complex trip to investigate the field of BINMs and their significant implications for creating and operating micro/nanodevices, particularly those used in the biomedical industry. We start by delving deeply into the idea of biologically inspired nanomaterials, illuminating the intrinsic functional possibilities they bring, and defining thestructure–function correlations observed in nature (Section 2). The many forms of BINMs and their key properties are then discussed (Section 3). The benefits of these intriguing nanomaterials in improving the performance of micro/nanodevices are underlined as we go along, from their flawless biocompatibility to their adaptability (Section 4). In other nanotechnology fields, a wide range of non-biomedical uses of BINMs are also covered (Section 5). The complex synthesis of BINMs, motivated by natural structures, biomolecules, and processes, is then covered in detail (Section 6). Design guidelines for BINM interfaces, emphasizing functionalization strategies and associated difficulties, significantly deepen our understanding (Section 7). We elaborate on the numerous uses of BINMs in micro/nanodevices, primarily focusing on the biomedical sector, including drug delivery systems, organ-on-chip technologies, wound healing approaches, and antimicrobial surfaces (Section 8). As this analysis draws to a close, we consider the ongoing difficulties associated with using BINMs in biomedical applications (Section 9). Finally, we believe in the prospects for the future and offer a few closing thoughts to summarize our discussion (Section 10). This article offers researchers, academics, and business executives a comprehensive grasp of the state of the art and the projected trajectory of BINMs in micro/nanodevices. Figure 1 represents the table of contents of this review article.

## 2. Bio-Inspired Nanomaterials: From Concept to Realization

The progression of BINMs serves as a testament to the notable amalgamation of biology and nanotechnology. They are designed to replicate the structures, functions, or processes observed in nature. This approach enables the development of novel materials that possess distinctive properties. The development of BINMs typically commences with a comprehensive comprehension of the biological system that researchers seek to emulate. The comprehensive understanding and replication of distinct structures, processes, or functions observed in nature at the nanoscale necessitate collaboration among biologists, chemists, and material scientists. The practical implementation of nanomaterials inspired by biological systems can be a multifaceted undertaking requiring meticulous planning and regulation. In certain instances, researchers can employ a biological process directly to synthesize nanomaterials. One illustrative instance involves using bacteria or fungi to generate nanoparticles (NPs), transforming these organisms into miniature factories for nanomaterial production.

**Figure 2:**
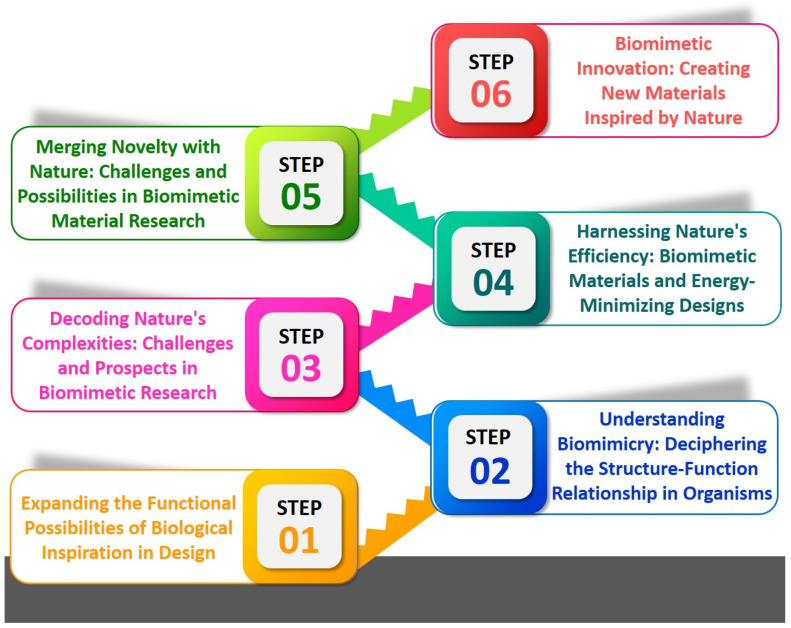
Implementation of BINM-based micro/nanodevices in biomedical applications using intensive research composed of six steps.

In some instances, scientists may be required to employ alternative approaches to accomplish their objectives. The potential application entails the development of artificial structures capable of autonomous assembly, emulating the structural characteristics observed in biological systems. One instance illustrating this phenomenon is the advancement in the creation of synthetic peptides capable of self-assembly into nanofibers, which resemble the nanofibers present in the extracellular matrix of various tissues [48]. Additionally, there exists the challenge of expanding the scale of these processes. Although these nanomaterials can be synthesized in a laboratory setting, scaling up the production process while preserving their intended characteristics poses a greater challenge. Furthermore, the realization of BINM concepts frequently necessitates meticulous optimization. It may be necessary to carefully adjust their properties to optimize the performance of these nanomaterials. This process may entail modifying various parameters, including the dimensions, morphology, or elemental composition of the nanomaterials. Developing BINMs involves a complex and intricate journey, necessitating a comprehensive comprehension of biology and nanotechnology. This research direction holds promise in generating novel materials that can effectively tackle various scientific and technological challenges. The application of biomimetics to multiple domains, such as design, product development, service enhancement, and biomedicine, can be facilitated through a basic research method comprising six distinct steps (Figure 2) [49].

### 2.1. Transforming the Functional Possibilities of Biological Inspiration in Design

Biomimicry, the process of using ideas from the natural world to address problems faced by humans, has long been at the forefront of ground-breaking discoveries. However, it is crucial to realize that biological things’ functional potential should not be replicated as it appears in nature. Instead, they should act as a springboard for creative, research-based designs. It has been seen in nature that millions of years of evolution have shaped these animals for certain functions in their settings, from the coordinated flight of birds to the exquisite designs on butterfly wings. It is possible that merely reproducing these capabilities will not satisfy the particular needs and limitations of human cultures and technology surroundings. Determining the basic principles and mechanisms that nature uses and then adapting or improving them for human use are crucial. The Japanese Shinkansen bullet train is one of the examples [50]. When these trains departed tunnels at high speeds, noise pollution posed a huge difficulty to the engineers. The engineers rebuilt the train’s nose by taking inspiration from the kingfisher, whose streamlined beak allows it to plunge into water with little splash. This improved speed and fuel economy while also reducing noise. Although nature served as the source of inspiration, the design was an adaption rather than a close match in this case.

The fundamental motivation should be broader despite the obvious economic attractiveness of such inventions. Discoveries or the confluence of disparate concepts can increase profitability and market supremacy. The objective is to improve the comfort and quality of human life, not just make money from a novelty. Designs that are straightforward but innovative, derived from nature but made specifically for people, have the power to change industries as diverse as transportation, healthcare, energy, and architecture. As a result, even while nature offers a priceless store of design solutions, the difficulty for innovators is in interpreting and implementing these solutions. The actual achievement is in developing goods or procedures that skillfully combine the brilliance of nature with the requirements and aspirations of people.

### 2.2. Understanding Biomimicry: Deciphering the Structure-Function Relationship in Organisms

Understanding the connection between an organism’s function and the governing principles of that function is a crucial component of biomimicry, a discipline that aims to mimic nature’s time-tested patterns and tactics. The development of bio-inspired designs and technologies is based on this understanding. To this aim, conducting a diligent study and assembling thorough databases to amass knowledge and utilize various materials according to their features is essential. In biomimicry, the interesting relationship between form and function is crucial. Designing self-cleaning materials, for instance, is influenced by the complex surface structure of a lotus leaf, which makes it water-repellent. A thorough knowledge of thisstructure–function link can be gained through cutting-edge scientific methods like scanning electron microscopy. This method makes it possible to notice the little details critical to an organism’s ability to operate, which is an essential first step in the biomimicry process.

Scanning electron microscopy provides a thorough image of the surface topography and composition of a material to understand better how an organism functions. For instance, the invention of specifically textured surfaces that limit fouling or microbial growth was motivated by observing the microscopic structure of a shark’s skin, which is made up of tiny, tooth-like scales known as denticles [51]. To effectively employ biomimicry, it is imperative to acquire a comprehensive understanding of the relationship between structure and function in living organisms, which should be underpinned by robust research and meticulously curated databases. This approach has the potential to further unlock the vast possibilities of nature-inspired concepts and technologies, thereby facilitating the development of a sustainable future.

### 2.3. Decoding Nature’s Complexities: Challenges and Prospects in Biomimetic Research

Biomimetics, the study and creation of engineering systems and contemporary technologies using biological techniques and systems found in nature, presents special potential and challenges. Understanding the intricate connections between organisms, their micro- and nanostructures, and their environment is perhaps the most important of them. Harnessing the potential of these structures requires understanding how they work, especially for those that have not yet been adequately investigated. These difficulties are multifaceted. For instance, an organism may use a certain structure to perform a given function in a particular environment, yet the same structure may be used otherwise in a different situation. Additionally, there could be less obvious tertiary or even secondary functions. Resolving this complex dance between structure and function that depends on the surrounding environment is like solving a complex puzzle for biomimetics. Understanding biological complexities and reproducing them in synthetic materials are complex tasks in biomimetic research. It takes skill and accuracy in material design and engineering to replicate the structures seen in nature, which frequently exist on the nano- or microscale [52,53].

The merging of biology, natural history, and material science is the next step in biomimetic research to address these issues. Each of these disciplines gives a unique viewpoint and set of instruments that can aid in revealing the mysteries of biological architecture. Understanding living things and how they work is made possible by biology, which serves as the basis for biomimicry. Natural history sheds light on how these processes have changed over time and their contributions to the organism’s success and survival. Last but not least, material science provides the skills and knowledge required to mimic these biological structures with artificial materials, enabling the implementation of biomimetic principles in practical settings. This integrative approach to biomimetics has great potential benefits. Medicine, architecture, energy, and manufacturing are just a few industries that might undergo a revolution if we can mimic and exploit the efficiency, adaptability, and sustainability inherent in natural systems. Though the road is difficult, the promise of biomimetic research keeps scientists and engineers motivated to discover the mechanisms behind nature’s intricate design.

### 2.4. Harnessing Nature’s Efficiency: Biomimetic Materials and Energy-Minimizing Designs

Exciting research prospects in the developing discipline of biomimicry are focused on identifying unique functional and environmental adaption strategies of organisms. Discovering how these organisms adopt energy-minimizing designs, a concept essential for our sustainable future, is a critical part of this frontier [54,55,56]. This is about using the creativity and efficiency of nature to inform and advance our designs and technologies. One successful instance of innovation is the development of antireflective coatings, which drew inspiration from an initially unremarkable source, namely the structure of a moth’s eye [57]. Despite their tiny size, moth eyes feature complex designs about 200 nm and astonishingly reflect visible light. The efficiency of solar panels can be increased by minimizing light reflection, and the legibility of electronic displays can be improved. Scientists have duplicated these nanostructures to create antireflective coatings, which have a variety of applications.

Remodeling hierarchical structures and the associated functions taken from nature is crucial in creating novel biomimetic materials. This procedure involves copying these structures and comprehending and using the guiding concepts. Then, designs and technologies that impact human society adapt and incorporate these concepts. Advancements in several sectors may result from creating novel materials motivated by the hierarchical structures found in nature [58]. Scientists have developed reusable, residue-free, and temperature-resistant adhesives by comprehending the nanoscale hair-like structures on a gecko’s feet.

### 2.5. Merging Novelty with Nature: Challenges and Possibilities in Biomimetic Material Research

Biomimetics is constantly growing, and new innovations and discoveries are routinely made. Integrating recently found materials with ongoing biomimetic research is crucial to this subject. This integration is believed to be crucial to comprehend the possible uses and constraints of such materials, opening up new avenues in technology and design. However, in order to fully fulfill this potential, it is imperative to develop a thorough grasp of both the advantages and disadvantages of biomimetics. Every newly discovered substance or method has special benefits and drawbacks. In contrast to conventional materials and processes, biomimetic designs, while frequently bringing about enhanced efficiency and sustainability, can also present cost, manufacturing complexity, or durability obstacles. Understanding the morphological and functional applications of novel materials is crucial, in addition to considering the pros and downsides. While the functional features describe how the material functions or interacts under various circumstances, the morphological qualities specify the material’s physical and structural properties. By gaining information into these areas, scientists can forecast how the material would perform in various applications and what adjustments might be required to maximize its performance.

Unexpected outcomes may arise from integrating novel materials into biomimetic designs [59,60,61]. These results need to be carefully examined and comprehended since they can indicate new applications for the materials or unforeseen limitations of the designs. Untangling these findings requires a systematic, step-by-step approach that progressively unveils the essence of the substance and its potential, much like peeling back the layers of an onion. It is difficult to advance in this field, it is true. The complicated and sophisticated nature of the systems being investigated and imitated makes biomimetic material research challenging. Nevertheless, despite the difficulties, there is an intense study going on because of the potential that biomimetics provides for developing future solutions that are more sustainable, effective, and innovative. There are many challenges in realizing the full potential of novel materials in biomimetics. Despite this, there is still a strong commitment to overcoming these obstacles since it is recognized that the benefits, such as advancing our technological skills and promoting sustainable practices, make the effort worthwhile.

### 2.6. Biomimetic Innovation: Creating New Materials Inspired by Nature

At its essence, biomimicry is about taking inspiration from nature to create new things. Analyzing the structure and function of biological components is a vital step in this process. This knowledge frequently acts as the starting point for creating new materials and the creative application of existing ones. For instance, new high-strength fibers have been developed due to the structural versatility of spider silk, a substance that is both stronger and lighter than steel. Similarly, the development of color-changing materials has been driven by the unique design of butterfly wings, which can reflect light without pigmentation. The fundamental principle is to gain knowledge from the complexity of natural structures and functions and then apply this knowledge to inspire and guide the creation of new materials. It is required to conduct thorough testing and analyses of the structures and operations of biological materials to accomplish this. This gives researchers important insights into these materials’ potential by enabling them to comprehend how they behave under diverse circumstances. These revelations can then influence the design and synthesis of novel materials with comparable properties.

Once these novel materials are created, they can be integrated with recent developments in various industries, including chemistry, nanotechnology, and medicine. This interdisciplinary approach can open up a wealth of cutting-edge uses that will considerably enhance human lives. For example, in the field of medicine, materials modeled after gecko feet are being created for their exceptional adhesive characteristics, which have the potential to revolutionize surgical techniques and wound healing [62]. The creation of catalysts based on enzymatic processes has been stimulated by biomimicry in chemistry. The design of water-repellent coverings in nanotechnology results from features like the nanoscale hairs that make lotus leaves self-cleaning [63]. Despite the enormous promise, it is necessary to recognize the difficulties. The intricacy of natural systems differs significantly from that of artificial systems; therefore, merely mimicking nature is not the solution. Instead, it is about comprehending and putting these biological systems’ core principles to use to develop novel, long-lasting, and efficient solutions.

## 3. Brief Overview of Micro/Nanodevices and Types of Bio-Inspired Nanomaterial

Micro/nanodevices frequently exhibit distinct properties and behaviors, which can be attributed to quantum effects and other phenomena that manifest exclusively at these reduced dimensions. They encompass a diverse array of instruments, such as sensors, actuators, and electronic components, among various others. Historically, micro/nanodevices have predominantly employed silicon-based materials due to their exceptional semiconductor characteristics, widespread availability, and well-developed knowledge of silicon processing methodologies. The significant impact of the semiconductor industry on this phenomenon can be attributed to its extensive utilization of silicon in producing microprocessors and various electronic components. Other materials, such as gallium arsenide, silicon carbide, and various polymers, ceramics, and metals, are employed per specific device specifications.

**Figure 3:**
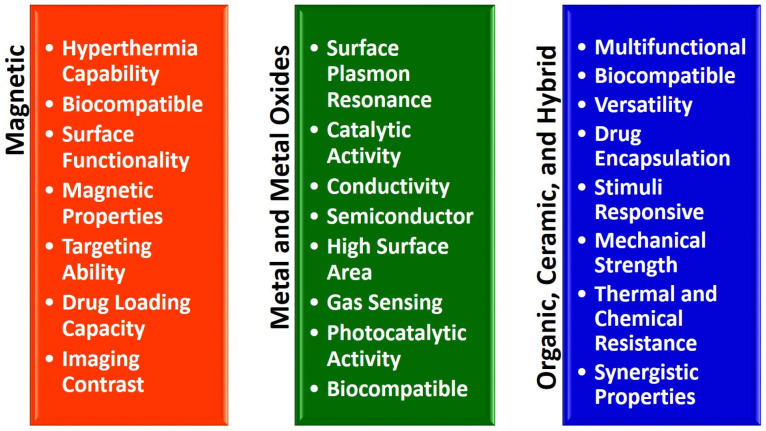
Types of BINMs and their unique characteristics have made them potential candidates for multiple biomedical applications.

The emergence of micro/nanodevices has led to notable advancements in biomedical applications. The diminutive dimensions of these entities facilitate engagements with biological systems at the cellular and molecular scale, thereby facilitating the development of accurate diagnostics, therapeutics, and research instruments. An illustration of the efficacy of nanoscale drug delivery systems lies in their ability to selectively target afflicted cells with minimal impact on surrounding healthy tissues. In diagnostics, they can swiftly identify disease biomarkers even at highly diluted levels, thereby facilitating timely identification and intervention [64]. Moreover, micro/nanodevices have been employed in tissue engineering and regenerative medicine to manipulate cellular behavior and facilitate tissue proliferation. Despite the considerable potential, several challenges need to be addressed. A notable obstacle lies in the manufacturing process, which necessitates the meticulous and consistent production of these devices at a miniature scale. One additional obstacle pertains to incorporating these devices into broader systems, necessitating the resolution of concerns related to connectivity, compatibility, and power management. There are other apprehensions regarding nanoscale materials’ potential health and environmental ramifications, as they exhibit distinct behaviors compared to their macroscale counterparts. Finally, the challenges pertaining to the stability, performance in varying conditions, and durability of micro/nanodevices are of utmost importance and require attention as we further exploit their capabilities. BINMs might be classified as magnetic biomimetic, metal and metal oxide biomimetic, and organic, ceramic, and hybrid biomimetic [65]. Figure 3 lists the types of BINMs and their unique characteristics that have made them potential candidates for multiple biomedical applications.

### 3.1. Magnetic BINMs

Magnetic BINMs nanoscale particles are intentionally designed and manipulated to replicate and imitate natural biological processes and structures. These particles leverage their inherent magnetic properties to serve a multitude of applications. The design of these NPs is influenced by biological systems, including cells, proteins, and viruses, to fabricate functional materials with distinctive characteristics. The bio-inspired nature of these NPs entails emulating specific attributes observed in biological systems. For instance, certain magnetic NPs are engineered to replicate the morphology and organization of specific cells, enabling them to engage with biological tissues selectively [66]. Individuals can imitate the actions of biomolecules, such as enzymes or receptors, to carry out specific tasks related to drug delivery or sensing. The manipulation of magnetic NPs by applying external magnetic fields enables precise control over their movement and facilitates targeted interactions within biological systems. The characteristic mentioned above is utilized in various biomedical contexts, including but not limited to drug administration, medical imaging, and the application of magnetic hyperthermia in cancer treatment. In general, magnetic NPs inspired by biological systems integrate the principles of nanotechnology and biomimicry to generate novel materials that hold promise for fields such as medicine, biotechnology, environmental remediation, and other domains.

Numerous magnetic BINMs have been successfully produced thanks to the application of biomimetic synthesis techniques, from the hard magnetic alloys FePt and CoPt to ferrimagnetic Fe_3_O_4_ found in magnetotactic bacteria (MTB) [67]. Because they use biological structures that can be altered through chemical or genetic engineering for certain functionalities and scalable production, biomimetic approaches have special advantages. Most previous publications on magnetic NP synthesis concentrated on physical and chemical techniques. However, more recent developments in magnetic BINMs have enabled the lab to replicate MTB-like chains of magnetic NPs, showcasing promising biomimetic techniques for biomedical applications [68]. They come in a variety of forms. One method uses magnetosome-associated MTB proteins to biomineralize Fe_3_O_4_ and produce polymer-coated and non-polymer-coated magnetite NPs. Chemotherapy, magnetic hyperthermia, enzyme immobilization, and photothermia are only a few of the uses for these NPs [69,70,71]. Purified and functionalized engineered structures derived from bacterial magnetosomes can be used as contrast agents in magnetic resonance imaging, magnetic particle imaging, and magnetic hyperthermia [72]. Hydroxyapatite (HAP)-coated magnetite NPs are a different class of magnetic BINMs created by mixing a liquid HAP precursor with a solution containing magnetite cores. These constructions help deliver genetic material, magnetic scaffold creation for bone tissue repair, and magnetic hyperthermia [73,74]. The design of biocatalysts for targeted enzyme prodrug therapy is made possible by combining magnetic NPs with active ingredients in a biomimetic matrix, such as SiO_2_ [75]. Methods for developing magnetic BINMs can be divided into two categories: those that use magnetic NPs that have already been obtained or are available commercially and those that make magnetic NPs from scratch. The latter group has a better chance of producing atomically precise structures that resemble their natural analogs. These techniques include recombinant MamC-based anaerobic biosynthesis [76], PEGylated human ferritin NP-based magnetite biomineralization [77], and encapsulation or biotinylation of isolated bacterial magnetosomes [72]. Despite a long research history, magnetic BINMs are not as commonly used in biomimetics as other materials. Nevertheless, recent developments in biomimetic synthesis and the distinctive characteristics of magnetic BNMs imply they have enormous potential for various biomedical applications.

### 3.2. Metal and Metal Oxide BINMs

Metal and metal oxide BINMs represent a captivating category of NPs that emulate natural structures and functionalities. Nanomaterials possess distinct characteristics that render them exceptionally well suited for various biomedical applications. Metal NPs, such as gold and silver, are frequently employed in biomedical research owing to their remarkable optical characteristics, substantial surface area, and adjustable surface chemistry. These technologies find utility in cancer treatment, precise administration of pharmaceuticals, and medical imaging. Gold NPs can undergo functionalization through the attachment of antibodies, enabling them to selectively target cancer cells and facilitate the direct administration of therapeutic agents to the tumor site [78]. Silver NPs possess antimicrobial properties, making them highly advantageous for wound dressings and antibacterial therapies [79]. Metal oxides, including iron oxide NPs, exhibit magnetic characteristics that make them well suited for various applications, such as magnetic targeting, hyperthermia-based cancer treatment, and the development of contrast agents for magnetic resonance imaging (MRI) [80]. External magnetic fields enable the precise localization of iron oxide NPs within the body, facilitating targeted drug delivery and localized therapeutic interventions. BINMs present a highly promising avenue for advancing various disciplines, including medicine, diagnostics, and therapeutics. The valuable attributes of NPs, such as their biocompatibility, functionality, and capacity to interact with biological systems, render them instrumental in addressing a wide range of health challenges and enhancing patient outcomes. Nevertheless, additional investigation is required to comprehensively comprehend their conduct within intricate biological settings and guarantee their reliability and effectiveness in clinical contexts.

By choosing particular micro- or nanoenvironments during synthesis, biomimetic synthesis techniques have been utilized to regulate the physical and chemical properties of metal and metal oxide nanomaterials. Several platforms have been used for biomimetic production of metal oxides, including ferritin, viral capsids, and bacterial cells. These platforms provide exact conditions for NP formation and produce narrow distributions in shape and size. These methods’ consideration of the environment and the possibility for expansion make them attractive. Due to its benefits, plant-based biomimetic synthesis of metal NPs has attracted interest recently, and researchers are investigating the mechanisms of nanomaterial synthesis and metal ion biological reduction in plants. Metallic BINMs have uses in the detection and eradication of contaminants. To develop sensors for biological substances and conduct research on nanotoxicology, biomimetic techniques can be used to make gold, silver, and bimetallic Ag-Au NPs [81]. For targeted drug delivery, biomimetic mineralization methods utilizing cubic nanostructures built on lipid membranes known as “cubosomes” are being investigated [82]. For the biomimetic synthesis of materials, metal-organic frameworks (MOFs) are another topic of study. They are useful for drug delivery, catalysis, and stabilizing biomacromolecules because they may be constructed with biomimetic active centers and restricted pockets [83].

### 3.3. Organic, Ceramic, and Hybrid BINMs

Organic, ceramic, and hybrid BINMs constitute a distinct category of NPs that exhibit various applications within biomedicine. Nanomaterials derive inspiration from natural structures and processes, showing distinctive properties that render them well suited for diverse biomedical applications. Organic BINMs are synthesized using naturally occurring molecules such as proteins, lipids, and carbohydrates. Biocompatibility is a characteristic exhibited by these entities, rendering them suitable for integration with biological systems. Moreover, they can be modified through engineering processes to acquire precise functionalities, such as targeted administration of pharmaceutical agents and facilitation of tissue regeneration. One illustrative instance involves the utilization of liposomes, which are lipid vesicles at the nanoscale level, to encapsulate drugs and facilitate their targeted delivery to precise locations within the human body [84]. This approach serves to mitigate adverse effects and enhance the efficacy of therapeutic interventions. Ceramic nanomaterials with bio-inspired characteristics, such as hydroxyapatite and silica NPs, exhibit a mineral composition resembling bones and teeth [85]. They are extensively utilized in bone tissue engineering, wherein they facilitate bone regeneration and augment the assimilation of implants into native bone tissue. Moreover, ceramic NPs exhibit considerable potential in drug delivery [86] and imaging [87] due to their inherent stability and biocompatibility. Hybrid BINMs amalgamate distinct material characteristics to attain heightened functionalities. Nanomaterials possess the capability to incorporate both organic and inorganic constituents, as well as to integrate magnetic attributes with organic coatings. An illustration can be found in the advancement of hybrid magnetic NPs designed for targeted drug delivery and hyperthermia-based cancer therapy [88]. A magnetic component facilitates convenient manipulation and precise localization within the human body. At the same time, implementing an organic coating ensures compatibility with biological systems and controlled release of therapeutic agents. In biomedicine, organic, ceramic, and hybrid BINMs exhibit significant promise. The valuable attributes of NPs, including their versatility, biocompatibility, and capacity for customization, render them highly advantageous in drug delivery, imaging, tissue engineering, and various other biomedical technologies. Nevertheless, it is imperative to thoroughly assess the safety and effectiveness of these novel nanomaterials before their extensive application in clinical settings.

Extensive research efforts have investigated BINMs comprising organic and ceramic constituents, demonstrating considerable potential in diverse biomedical domains. Proteins and peptides serve as templates for regulating the synthesis and self-assembly of organic BINMs, facilitating the development of multifunctional materials possessing distinct structures and functionalities. Peptoids, a biomimetic polymer category, are widely recognized as versatile constituents for constructing hierarchical BINMs [89]. Research in the ceramic biomaterial and nanomaterial domain is primarily centered around developing scaffolds that emulate the structural and functional characteristics of natural tissues, particularly bone. Ceramic structures with a high degree of porosity, similar in structure to cancellous bone, have been successfully manufactured to facilitate the ingrowth of cells and the formation of new tissue. Biomimetic ceramic scaffolds are infused with therapeutic molecules to enhance their biological efficacy. Hybrid BINMs are being investigated for their potential applications in tissue engineering in orthopedics and dentistry. There is ongoing research and development in drug delivery carriers, specifically on membrane-camouflaged NPs.

## 4. Advantages of Bio-Inspired Nanomaterials in Micro/Nanodevices

**Figure 4:**
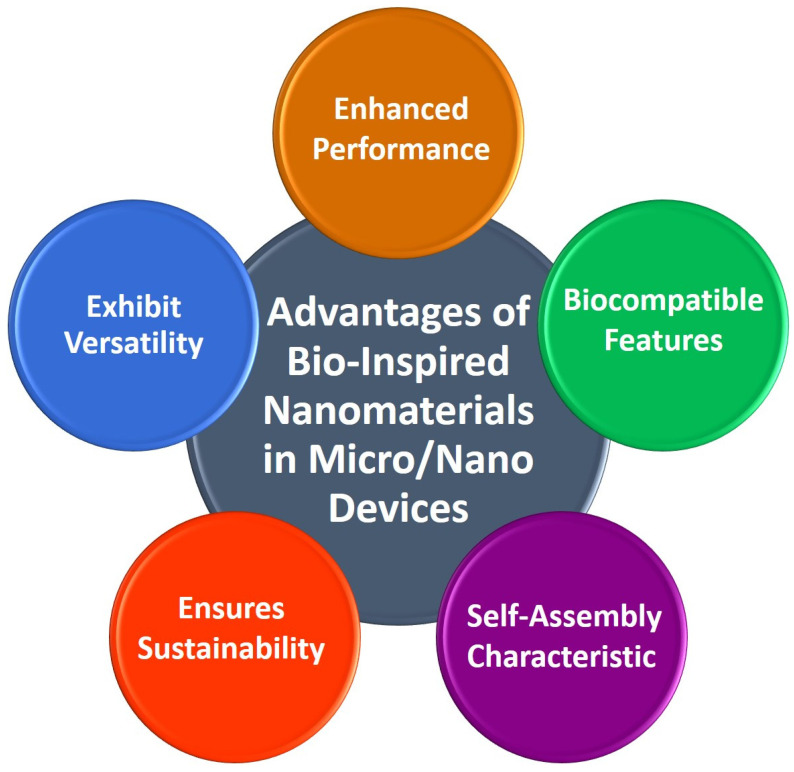
Advantages of BINMs in micro/nanodevices in biomedical applications.

The utilization of BINMs has emerged as a novel approach to advancing micro/nanodevices. Nanomaterials that draw inspiration from biological systems present a unique strategy for addressing the difficulties associated with device miniaturization while simultaneously improving performance, versatility, and biocompatibility. The utilization of BINMs in micro/nanodevices encompasses a wide range of disciplines, such as electronics, optics, environmental science, and biomedicine. The electronics field is investigating the potential of bio-inspired materials, such as protein-based nanowires and biogenic semiconductors, to develop novel electronic devices with distinctive electronic characteristics. BINMs have significantly transformed micro/nanodevices within the biomedical field. For example, nanomaterials have been utilized by drug delivery systems to augment the precision of drug administration and regulate the release of therapeutic agents, leading to notable advancements in treatment efficacy. BINMs exhibited enhanced sensitivity and specificity in detecting biomarkers, thus facilitating the potential for early disease diagnosis and disease monitoring. They possess numerous advantageous characteristics when integrated into micro/nanodevices, such as improved performance, biocompatibility, self-assembly capabilities, sustainability, and versatility (Figure 4).

### 4.1. Enhanced Performance

The utilization of BINMs has the potential to greatly enhance the performance of micro/nanodevices by capitalizing on their distinct properties. The observed enhancement in performance can be attributed to the remarkable characteristics that nature has developed over an extensive period of time. One potential application of nanomaterials is their ability to replicate the adhesive properties observed in gecko feet [90]. This unique characteristic has garnered significant attention due to its exceptional adhesive strength. Consequently, nanomaterials with gecko-inspired adhesion can potentially revolutionize the development of micro-robots and wearable devices, enabling them to adhere to diverse surfaces [91]. Likewise, the utilization of nanomaterials inspired by shark skin, which possesses characteristics that reduce drag, holds potential for enhancing the energy efficiency of microfluidic devices [92].

### 4.2. Biocompatibility

One notable advantage of numerous nanomaterials inspired by biological systems is their biocompatibility. Nanomaterials that draw inspiration from or are derived from biological entities possess an inherent compatibility with biological systems. The compatibility between these materials and living tissues or cells mitigates potential adverse reactions. In drug delivery, biocompatible nanomaterials enable the transportation of therapeutic agents within the human body while mitigating the risk of eliciting detrimental immune responses [93]. Likewise, in biosensing, NPs can be utilized for extended monitoring periods without inducing any adverse tissue irritation or rejection [94].

### 4.3. Self-Assembly

Self-assembly is a captivating characteristic observed in numerous biological systems, wherein complex structures are formed spontaneously [95]. BINMs frequently inherit this intriguing property. A range of factors, including pH, temperature, and ionic strength, can control the process of self-assembly [96]. This ability to direct self-assembly can be utilized to construct complex structures with minimal external intervention. This streamlined manufacturing process offers the potential to create intricate designs for devices that would present significant challenges or even be unattainable through conventional fabrication methods [97].

### 4.4. Sustainability

The design and synthesis of BINMs frequently incorporate the principles of green chemistry and biomimicry, contributing to the promotion of sustainability [98]. This methodology has the potential to facilitate the advancement of fabrication processes and devices that are environmentally sustainable. Numerous BINMs can be synthesized using gentle conditions, devoid of toxic solvents or by-products. Moreover, certain nanomaterials can undergo biodegradation, thereby mitigating their environmental impact upon completing their functional lifespan.

### 4.5. Versatility

The extensive array of biological systems that serve as sources of inspiration for the design of nanomaterials provides a wide spectrum of potential applications. The potential of BINMs is vast, as demonstrated by their ability to replicate the light-harvesting capabilities of photosynthetic organisms to enhance solar cells [99] or imitate the structural color found in butterfly wings to improve optical devices [100]. These capabilities hold significant promise in developing materials with customized properties that can effectively fulfill the distinct demands of diverse applications, thereby expanding the possibilities for advanced micro/nanodevices.

## 5. Bio-Inspired Nanomaterials in Micro/Nanodevices

**Figure 5:**
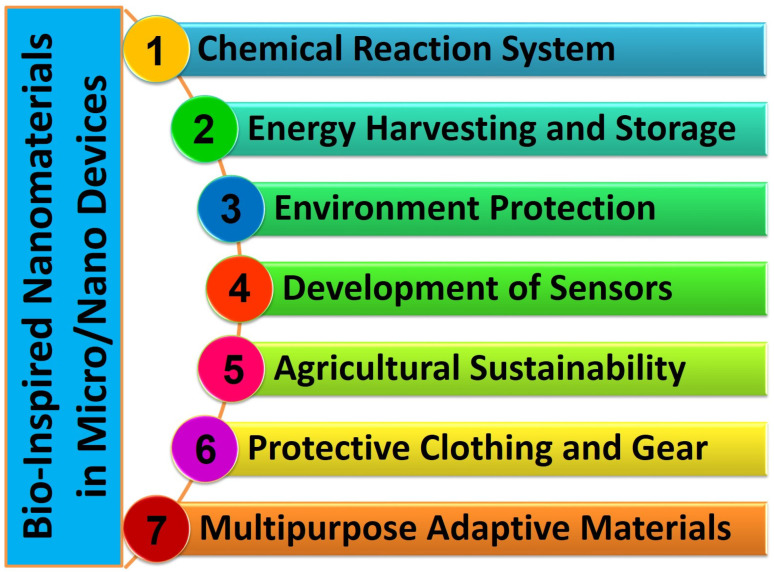
The applications of BINMs in micro/nanodevices other than biomedicine.

The utilization of BINMs in micro/nanodevices represents the integration of intricate designs found in the natural world with the capabilities of modern nanotechnology. This convergence establishes a mutually beneficial relationship with potential remarkable advancements in various domains. These materials draw inspiration from the distinctive characteristics displayed by biological organisms. By leveraging these properties, they enable the development and production of micro/nanodevices that offer improved performance, biocompatibility, self-assembly capabilities, sustainability, and versatility. The effective integration of these materials in various devices is evident in multiple instances, such as using self-cleaning solar panels, dry adhesives for micro-robots, photonic sensors, drug delivery systems, and other notable applications [101,102,103,104]. The advent of BINMs in micro/nanodevices signifies a significant advancement in technology, medicine, and environmental sustainability, showcasing the extensive possibilities of biomimicry on the nanoscale. The applications of BINMs in micro/nanodevices other than biomedicine have been summarized in Figure 5, while Table 1 summarizes the subsections under this section and lists the applications of BINMs in various types of micro/nanodevices belonging to diverse nanotechnology domains.

### 5.1. Chemical Reaction Systems

The utilization of BINMs is of significant importance in chemical synthesis and reactions, as it draws inspiration from nature’s intricate designs to enhance various aspects such as efficiency, selectivity, and sustainability. Chemical processes are frequently improved by emulating the hierarchical structure and multifunctionality observed in biological systems, such as enzyme-catalyzed reactions. For example, the development of bio-inspired catalysts, which draw inspiration from the highly efficient and specific catalytic mechanisms observed in biological systems, facilitates the execution of reactions with enhanced selectivity and reduced environmental impact [105,106]. This approach effectively minimizes both waste generation and energy consumption. The phenomenon of self-assembly, which is widely observed in nature, is also utilized as a reliable technique in producing nanomaterials. This technique enables the creation of intricate structures using mild conditions. In addition, the utilization of BINMs that replicate the biomineralization mechanism observed in corals has enabled the synthesis of nanocrystalline materials under ambient conditions [107]. This advancement holds significant potential for their application in various chemical reactions. In general, the utilization of BINMs in the context of chemical synthesis and reactions is a compelling demonstration of the efficacy of leveraging natural principles to propel the field of synthetic chemistry forward [108,109].

The synthesis of ammonia through the electrocatalytic reduction of nitrate/nitrite can be conducted using bio-inspired metalloenzymes [110]. Drawing inspiration from the natural NO_3_^−^ reductase, utilizing bio-inspired metalloenzymes presents a promising alternative to metal nanomaterials. This substitution can significantly enhance the electrocatalytic performance of NO_3_^−^/NO_2_^−^ reduction to NH_3_ (NRA) and improve NH_3_ selectivity in a neutral environment. Ford and his colleagues conducted a research study to create an iron catalyst influenced by the active sites found in NO_3_^−^ reductase enzymes. This catalyst was intended to treat industrial wastewater in challenging environmental conditions [111]. The catalyst possesses a secondary coordination sphere that assists in oxyanion deoxygenation. The reduction of oxyanions forms a Fe(III)-oxo species, which subsequently acts as a catalyst, facilitating regeneration while concurrently releasing water in the presence of protons and electrons. Using the bio-inspired iron catalyst in NRA has offered a sustainable approach for effectively utilizing the nitrogen resource. Through electrostatic interactions, a biomimetic nickel bis-diphosphine complex fixed on altered carbon nanotubes (CNTs) contained the amino acid arginine in the outer coordination sphere [112]. With a catalytic selectivity for H_2_ oxidation at all pH levels, the functionalized redox nanomaterial demonstrates reversible electrocatalytic activity for the H_2_/2H^+^ interconversion from pH 0 to 9. The high activity of the complex over a broad pH range enables us to integrate this BINM either in a proton exchange membrane fuel cell (PEMFC) employing Pt/C at the cathode or in an enzymatic fuel cell in conjunction with a multicopper oxidase at the cathode. Comparing the Ni-based PEMFC to a full-Pt traditional PEMFC, its maximum output is just six times lower at 14 mW cm^2^. A new efficiency record for a hydrogen biofuel cell using base metal catalysts is set by the Pt-free enzyme-based fuel cell, which produces 2 mW cm^2^.

### 5.2. Energy Harvesting and Storage

Using BINMs in the energy sector has sparked the development of ground-breaking strategies for effective energy generation, storage, and conservation. These materials make creating more effective and sustainable energy systems easier by taking their design cues from nature’s perfectly regulated energy processes. For instance, the distinctive light-harvesting systems seen in photosynthetic organisms have been imitated to increase the effectiveness of photovoltaic cells, allowing them to more efficiently gather and transform sunlight into electricity [113,114,115]. BINMs have also influenced the creation of durable and light-weight materials for wind turbine blades, improving both the performance and longevity of these devices [116,117]. Furthermore, progress has been made in designing BINMs for energy storage devices, including batteries and supercapacitors. For instance, bee-hive-inspired honeycomb shapes have been employed to make electrodes with a high surface-to-volume ratio, increasing their energy storage ability [118,119,120]. The cooling systems found in some animals have influenced the design of materials for effective heat dissipation in energy devices.

### 5.3. Environmental Protection and Sustainability

Using nature’s design principles to address urgent environmental concerns, BINMs have opened new vistas for environmental preservation and sustainability. For example, the distinctive capacity of lotus leaves to self-clean has sparked the creation of coatings based on nanomaterials that lessen the need for harsh cleaning agents, thereby reducing water pollution. Similar to photocatalytic materials, which help break down pollutants when exposed to sunlight, photocatalytic materials are inspired by photosynthesis in plants and offer a promising alternative for air purification and wastewater treatment. Indirectly reducing the carbon footprint, BINMs have a considerable impact on the energy sector, helping to create better energy storage devices and increasing solar cell efficiency. Additionally, sustainable BINMs that are recyclable or biodegradable have been created using the concepts of biomimicry, supporting a circular economy.

### 5.4. Development of Sensors

BINMs are transforming sensor technology by boosting sensitivity, specificity, and dependability. For instance, photonic sensors that can sense minute changes in light, temperature, and pressure have been developed using structures that mirror the delicate architecture of butterfly wings [121]. Developing tactile sensors based on nanomaterials is highly sensitive to various stresses [122]. Similarly, acoustic sensors for detecting light sounds or vibrations have been created using the structure of spider silk, which is well recognized for its sensitivity to air movements [123]. The invention of microphones that simulate the highly directed hearing of the parasitic Ormiaochracea fly was made possible by the development of acoustic sensors, inspired by spider silk’s sensitivity to air movements. Chemical sensors that can detect trace amounts of explosives, narcotics, or other compounds, similar to dogs’ noses, have been developed. These sensors are based on the exceptional sense of smell that animals possess. Identical to the infrared-sensing organs of the pit viper, thermal sensors, modeled after the heat-sensing abilities of some snakes, have led to devices that can detect temperature changes without being impacted by ambient temperature. The biocompatibility and high surface-area-to-volume ratio of several of these materials make them excellent for detecting biomolecules at very low concentrations, which has important implications for biosensors. Utilizing the special qualities of BINMs, sensors can be developed that outperform conventional designs in terms of performance, flexibility, and versatility, with uses in various fields, including security, healthcare, and environmental monitoring.

### 5.5. Agricultural Sustainability

BINMs can potentially bring about significant transformations in the agriculture and food sectors, presenting viable solutions to key challenges these industries face. The applications of these technologies encompass precision agriculture, wherein bio-inspired nanosensors are employed to monitor soil conditions and crop health, drawing inspiration from the moisture detection mechanism found in plant roots [102,124,125]. This enables the optimization of resource utilization. In pest and disease management, NPs that draw inspiration from naturally occurring plant or microbial compounds, such as those that imitate the pyrethrins found in chrysanthemums, can selectively target particular pests or pathogens [126]. Within the realm of the food industry, nanomaterials play a significant role in the development of intelligent packaging [127]. One notable application involves utilizing nanosensors capable of detecting ethylene levels, a naturally occurring compound that indicates fruit ripening [128]. By employing such nanosensors, monitoring changes in food quality and minimizing wastage are possible. In addition, biosensors enhance food safety by emulating the immune response, enabling the prompt identification of foodborne pathogens such as *E. coli* or *Salmonella* [129]. The enhancement of nutrient delivery is achieved by employing nano-encapsulation techniques that draw inspiration from inherent cellular mechanisms, thereby augmenting the assimilation of nutrients or probiotics. The field of waste management stands to gain advantages from utilizing BINMs, specifically those that imitate the natural catalysts found in the gut of termites. These nanomaterials can expedite the decomposition process of agricultural waste, resulting in the production of valuable resources such as biofuel or compost [7,130]. Furthermore, implementing water purification techniques inspired by the physiological processes observed in xylem tissues of plant species such as pine trees plays a crucial role in enhancing the safety of water used for irrigation purposes [131]. Using BINM applications offers a collective approach toward achieving enhanced global food security and environmental sustainability, resulting in more sustainable, efficient, and effective solutions.

**Table 1:** A thorough summary of the subsections under this section and a list of the applications of BINMs in various types of micro/nanodevices belonging to diverse nanotechnology domains.

Sector	Devices	Bio-Inspiration	Mechanism	Applications	Refs.
Synthesis	Catalytic converter	Enzymes/natural catalyst Peptide sequence Luffa sponge	Electrocatalysis	Water splitting Oxygen reduction CO_2_ reduction Metal nanoparticle (MNP) fabrication	[132,133,134]
	Photovoltaic device Fog harvester	Photosynthesis Butterfly wings	Electrochemical Photocatalysis	Water splitting Fog harvesting	[135,136,137]
Energy	Microbial biofuel cells	Photosynthesis Hydrogenases in microorganisms	Electrochemical Photocatalysis	Hydrogen production Energy production	[138,139,140,141]
	Electrodes	Bee honeycomb	Proton conduction as an electrode Electrochemical	Conversion of fuel energies into electricity Long-term energy conversion, transfer, and storage	[142,143,144,145]
	Solar cells	Photosynthesis Peptide nanomaterials Virus Cobweb	Photovoltaic	Electricity generation	[146,147,148,149,150]
	Battery and supercapacitors	Benzoquinone (BQ) in photosystem Adenine in DNA Human tissues Nanocluster arrays on a lotus leaf M13 virus protein shells Tobacco mosaic virus DNA	Electrochemical	Supercapacitor Li-ion battery electrode Li-sulfide batteries Rechargeable batteries	[136,151,152,153,154,155,156,157,158]
Environment	Hybrid photocatalysts Adsorbent Magnetic polymer nanocomposites Ultrafiltration membrane	Enzymes Peptides Biomolecules	Photocatalysis Adsorption Magnetism Ultrafiltration	Environmental detoxification Metal removal Dye removal Saline water separation Oil separation	[108,109,130,159,160,161,162]
	Electrochemical biosensor	RNA Nicking enzymes	Electrochemical sensing	Mercury identification Detection of *Salmonella enteritidis*	[163,164]
	Hybrid membranes	Aggregated amyloid protein fibrils	Filtration	Heavy metal removal	[165,166]
	Biosorbent	Bacteria	Adsorption	Elimination of Cd	[167]
	High-performance nanofilter	Tau protein *Moringaoleifera* pods	Nanofiltration	Air purification	[168,169]
Sensors	Potentiometric e-tongue	Tongue	Field-effect-transistor-based	Detecting the bitterness	[170,171]
	Organoid-based biosensor	Taste bud	Electrophysiological signals	Taste sensation	[172]
	Enzyme biosensor	Horseradish peroxidase	Electrochemical	Detection of H_2_O_2_	[173]
	Olfactory biosensor	Cardiomyocytes	Electrochemical	Odor detection	[174,175]
	Potentiometric sweetness sensor	Sweetness sensor GL1	Potentiometry	Detecting the sweetness	[176]
	Chemiresistive sensor	Sensor organ	Electrochemical	Detection of N_2_	[177]
	Photonic sensor	Morpho butterfly scales	Optoelectrochemical	Detection of H_2_, CO, and CO_2_	[178]
	Photonic nose	Turkey skin M-13 bacteriophage	Colorimetric	Detection of molecules	[179,180,181]
	Acoustic sensors	Spider slit organ Lotus leaf	Electrical	Voice recognition	[182]
		OrmiaOchracea fly	Electro-mechanical	Direction finding sensor	[183]
	Infrared sensor	Snake skin	Photomechanical	IR sensing systems	[184]
	Hydrodynamic sensors	Fish and some amphibians	Electro-mechanical	Hydrodynamic artificial velocity sensor	[185]
	Humidity sensors	Spider silk	Electrochemical Biological structures Electrical conductivity Transduction mechanisms	Humidity and strain detection	[186]
	Motion sensors	Snake movement	Electro-mechanical	Robot	[187]
	Magnetic sensors	Pigeons’ magnetoreception ability	Electromagnetic Magnetoreception Signal transduction Miniaturization and integration	Wastewater treatment	[188]
Protective Clothing and Gear	Smart fabric	Lotus leaf Algae eyespot-stigmata design Touch-me-not (Mimosa sps.) pulvinus Pine cone Chameleon skin Fish scale Mammalian tissue Spider silk Firefly glow Shark skin Mammal skin Spider silk Chameleon Cactus spines	Microfabrication Mechanochromic Photonic elastomer Biomimetic structures Sensing and actuating system Self-regulation Energy harvesting and storage	Anti-dust cloth Water-repellent fabrics Light- and touch-sensitive apparel Smart breathing fabrics Camouflage apparel Self-healing fabric Anti-tear fabric design E-circuited luminescent fabrics Antibacterial clothing Thermo- and pressure-sensitive fabric Self-adapting textiles Self-hydrated clothing	[189,190,191,192,193,194,195,196]
Multipurpose Adaptive Materials	Self-healing materials	Wound healing Bone remodeling Plant healing and regeneration Blood clotting and vascular repair Microbial biofilms Skin healing	Molecular mobility Triggered response Microcapsulation Dynamic covalent bonding Autonomic healing	Field-effect transistors Pressure sensors Strain sensors Chemical sensors Triboelectric nanogenerators Soft actuators Smart coating	[197,198,199,200]
	Shape-memory materials	Creatures reducing impact damage Muscle contractions Insect wing folding Plant movements Tendons and ligaments Caterpillar and snake movements Soft tissues	Two-way shape memory effect Phase transitions Molecular reconfiguration Energy storage and release Microstructure design	Biomedical devices Aerospace engineering Robotics Textiles	[201,202,203,204,205]
	Responsive surfaces	Lotus leaf Gecko adhesion Butterfly wing Shark skin Mussel adhesion Pinecone closing Venus flytrap	Hierarchical structures Stimulus-responsive materials Self-assembly Wetting and capillary forces Surface gradients Biomolecular interactions	Self-cleaning coatings Anti-fouling surfaces Stimuli-responsive materials Environmental monitoring	[206,207,208,209,210,211]
	Multifunctional composites	Bone structure Plant fiber Nacre Spider silk Biomineralization processes	Synergy of materials Hierarchical structure Synergistic interfaces Functionalization and integration	Aerospace and automotive industries Energy harvesting and storage Protective coatings Robotics	[212,213,214,215]

### 5.6. Protective Clothing and Gear

The utilization of BINMs exhibits significant promise in augmenting the safety, performance, and comfort attributes of protective clothing and gear. For example, the distinct denticles found on the skin of sharks, which can impede the growth of bacteria, serve as a source of inspiration for developing materials used in protective clothing. These materials exhibit similar properties by effectively resisting microbial presence, thereby mitigating the risks of infection in environments where individuals are highly exposed to such hazards. The remarkable mechanical properties of spider silk have been replicated in nanoscale architectures, resulting in materials that possess a unique combination of strength, flexibility, and low weight. These materials have applications in various protective gear, such as bulletproof vests and helmets, where their exceptional properties are highly advantageous. The phenomenon of structural coloration observed in peacock feathers, wherein color changes occur in response to environmental stimuli, provides a valuable model for developing materials capable of indicating hazardous conditions via color alterations. The water retention abilities exhibited by cactus spines serve as a source of inspiration for developing survival suits that can extract moisture from the atmosphere in arid environments. Moreover, the phenomenon known as the self-cleaning “lotus effect,” observed in the leaves of lotus plants, has prompted the advancement of self-cleaning materials that are particularly suitable for use in protective garments within environments characterized by dirt or contamination.

### 5.7. Multipurpose Adaptive Materials

The utilization of BINMs shows great potential in multipurpose adaptive materials (MAMs) for their ability to modify their properties according to environmental variations. Researchers have developed self-healing materials that exhibit automatic repair mechanisms, drawing inspiration from the regenerative capabilities observed in starfish and salamanders. These materials mimic the natural healing processes found in living organisms. Shape-memory materials have been engineered to imitate the adaptive response of the Venus flytrap to external stimuli. These materials have found applications in various fields, including smart textiles and precise administration of pharmaceuticals. The remarkable hydrophobic characteristics exhibited by lotus leaves have served as a source of inspiration for developing responsive surfaces that can repel water and dirt. These surfaces have practical utility in various domains, such as self-cleaning windows and anti-fouling coatings for maritime vessels. Scientists have utilized the heat-resistant adaptations of the Saharan silver ant as inspiration to create energy-efficient materials that can passively cool, thereby decreasing the need for energy-consuming air conditioning systems. Furthermore, the hierarchical arrangement of nacre, also known as the mother of pearl, has served as a source of inspiration for the development of multifunctional composites that possess a combination of strength and toughness. These composites have proven valuable in applications such as protective coatings and producing durable construction materials.

## 6. Synthesis of Bio-Inspired Biomedical Nanomaterials

**Figure 6:**
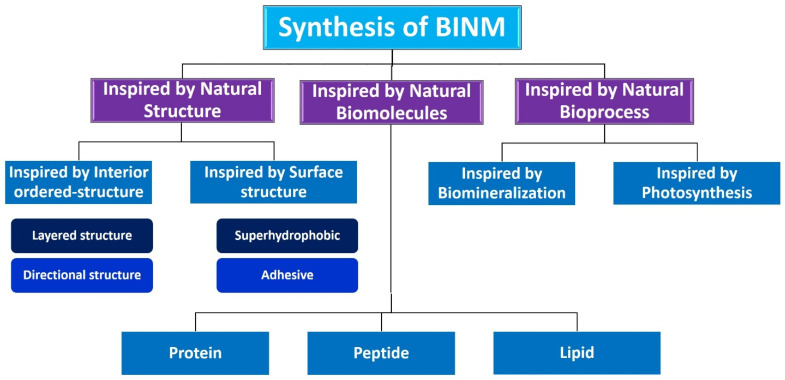
Synthesis of BINM suitable for biomedical applications.

Living things have developed the ideal arrangements of their structures, parts, and functions, giving rise to special qualities like the great strength of bones, the hardness of enamel, and the capacity of shark skin to lessen fluid drag. In recent decades, an attempt has been made to comprehend the connection between these elements in high-performance natural materials. Some processes, such as the layered structure of nacre for improved mechanical characteristics, the nanostructure of lotus leaves for hydrophobic properties, and the proteins in the feet of Mytilusedulis for adhesion, have been postulated. High-performance artificial materials have been created using these concepts in industries like energy, architecture, aircraft, and biomedicine. New synthesis techniques have also been put forth to better replicate the hierarchical components or structures of natural materials. Natural bioprocesses, including biomineralization, cell metabolism, and photosynthesis, that are essential to developing and operating natural biological systems have also received attention. These biological processes have advantages over artificial synthetic ones since they frequently occur in calm environments. A new study area called “bioprocess-inspired fabrication,” which merges biology, life science, and material science, aims to create novel synthesis methods inspired by biological processes in nature. High-performance biomaterials are being developed using bio-inspired techniques for tissue regeneration, medication delivery, biosensing, and monitoring applications. To build biomimetic scaffolds for bone tissue engineering or to use mussel-inspired bioadhesives for skin wound healing, these methodologies imitate natural structures, such as the hierarchical structure of bone. The synthesis of BINMs might be divided into three categories (Figure 6): synthesis inspired by natural structure/components, synthesis inspired by biomolecules, and synthesis inspired by bioprocesses [21].

### 6.1. Inspired by Natural Structures

The structural characteristics of a material are closely associated with its physical and chemical properties. Numerous natural substances exhibit intricate and well-organized patterns across various levels, such as the “brick and mortar” arrangement observed in nacre, the collagen fibers in a bone that are mineralized directionally, and the nanostructures resembling branches found on the surface of a lotus leaf. The exceptional performance of these distinct structures can be attributed to their specialized functions. At present, replicating the composition and arrangement found in natural materials is a prominent approach in biomedical materials to improve their mechanical strength, adhesion, and antibacterial characteristics.

#### 6.1.1. Inspired by Interior Ordered Structure

Numerous natural materials exhibit remarkable mechanical properties, including strength, toughness, and lightness, despite comprising constituent components of lower inherent strength. The primary reason for this phenomenon can be attributed to the multi-scale hierarchical structures and diverse failure mechanisms observed in various materials. In tissue engineering, biomedical materials must have exceptional biocompatibility and sufficient mechanical properties. Within this framework, we shall present several noteworthy internal ordered structures observed in the natural world. These structures encompass the “brick and mortar” layered structure, the Bouligand structure, and the directional arrangement structures evident in enamel and bone. These structures are prototypes for developing biomedical materials that exhibit enhanced mechanical strength. Nanomaterials, with potential for biomedical applications, can be synthesized by the inspiration of the layered structure or multi-directional ordered structure of nature.

The distinctive layered structure of nacre, resembling bricks, has served as a significant source of inspiration in advancing biomaterials with exceptional performance capabilities. The fantastic mechanical qualities of this structure, renowned for its great strength and toughness, have motivated researchers to explore the intricacies of reproducing similar layered nanostructures in order to attain exceptional mechanical characteristics in synthetic biomaterials. The development of nacre-like films by Yoo et al. is a notable achievement in this field [216]. The aforementioned nanocomposite films, composed of structured boron nitride nanosheets (BNNSs) and gelatin, were developed by leveraging the electrostatic attractions between the charged groups of gelatin and BNNSs. To improve self-assembly and the connection at the interface of these components, BNNSs were enhanced with hyperbranched polyglycerol. The alignment of the BNNSs on a 2D plane could be adjusted by increasing the BNNS quantity or through a specific functionalization technique, resulting in a shift from a chaotic orientation to a structured brick-and-mortar arrangement. By varying the BNNS and gelatin mixture in the composite and adjusting the BNNS arrangement, one can modulate the nanocomposite’s mechanical attributes, such as its strength and rigidity. This adjustment produces a substance with mechanical qualities mirroring human cortical bone. Preliminary in vitro tests showed that this BNNS/gelatin blend could promote attachment, sustenance, and growth of adipose-derived stem cells, marking its potential in the biomedical sector. The combined mechanical and biological results hint at the material’s potential applications in medical fields, especially tissue restoration. Similarly, Zhang et al. took inspiration from nacre to address the longstanding issue of inadequate strength in conventional guided bone regeneration (GBR) membranes. By organizing graphene oxide nanosheets in a nacre-like fashion, they successfully amplified the mechanical strength of the GBR membrane [217]. Nevertheless, a constraint arose regarding the dimensions and structure of these planar membranes. The researchers were unable to manage substantial bone deficiencies effectively. One potential approach that has been suggested is the conversion of these membranes into cylindrical scaffolds with three-dimensional structures [218,219]. This particular strategy shows potential for future developments in bone restoration. The application of bi-directional freezing technology achieved the synthesis of a silicate-based bioceramic composite. This innovative approach resulted in the formation of an ordered lamellar microstructure, which bears resemblance to naturally occurring layered structures. The material exhibits increased strength and facilitates a regulated discharge of bioactive ions, hence expanding its possible uses [220]. Moving beyond nacre, the Bouligand structure, commonly found in entities like fish scales and crab shells, has also captured the attention of biomaterial developers. Li et al.’s development of a chitosan film derived from crab shells beautifully replicates this dense Bouligand structure, offering increased tensile strength and inherent antibacterial properties [221]. Furthermore, Han et al. innovatively transformed fish scales, using in situ mineralization of calcium silicate, into scaffolds for tendon repair, highlighting the diverse potential of these natural structures [222]. These investigations highlight the possibility of combining natural structural influences with contemporary technologies. They demonstrate the progress in synthesizing biomaterials inspired by biological systems and indicate the numerous avenues for further investigation. The incorporation of natural structures alongside bioactive molecules holds the potential to facilitate the development of a novel cohort of multifunctional biomaterials that are customized for distinct biological purposes. As we progress, a collaborative endeavor to extract and incorporate knowledge from these studies might establish the trajectory for future investigations in the field of multifunctional biomaterials.

**Figure 7:**
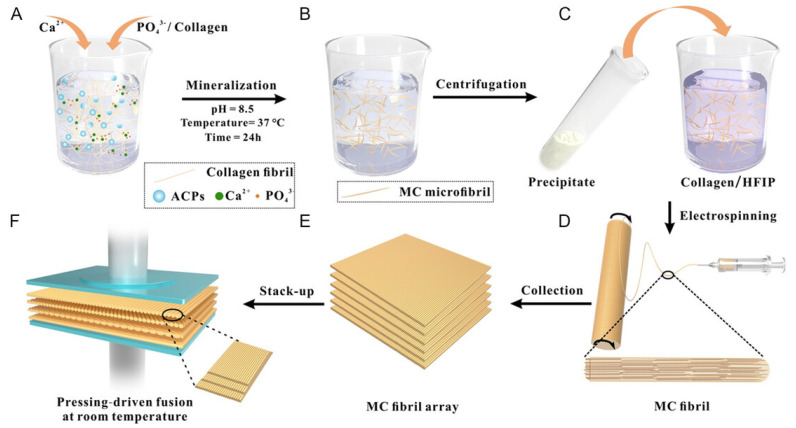
Schematic representation of the “multiscale cascade regulation” approach employed in the fabrication of artificial lamellar bone (ALB). (**A**) The process of calcium phosphate mineralization mediated by collagen. (**B**) The mineralized collagen (MC) microfibril precipitate obtained through centrifugation. (**C**) The electrospinning sols prepared by incorporating MC microfibril into a solution of collagen and HFIP. (**D**) Fabrication of MC fibrils through electrospinning. (**E**) An aligned array of MC (microcrystalline) fibrils obtained using a roller collector. (**F**) Bulk aluminum boride (ALB) forms through pressure-driven fusion at ambient temperature. Reprinted with permission from Ref. [226], Copyright 2023, Authors. CC-BY.

**Figure 8:**
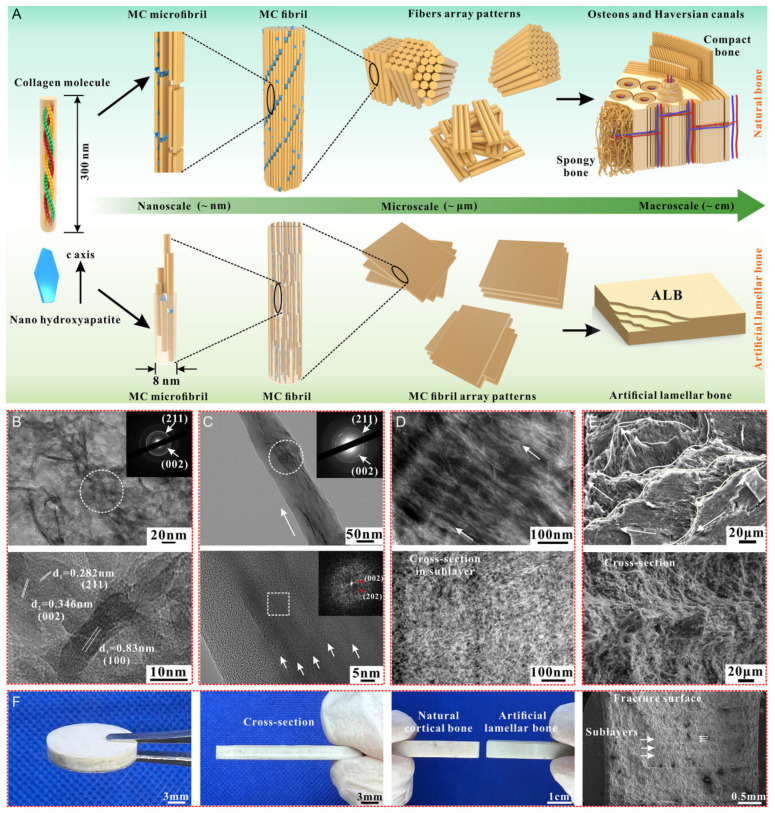
The illustration of the multiscale analysis and morphological features of the Asian longhorned beetle (ALB) and its comparison to natural bone. (**A**) Hierarchical organization from nanoscale to macroscale in natural bone and synthetic ALB is examined. (**B**) Transmission electron microscopy (TEM) images reveal MC microfibrils, with the inset showing the electron diffraction pattern identified as HA. High-magnification TEM images at the bottom visualize the interplanar spacings of the HA crystalline lattice planes ((211), (002), and (100)). (**C**) TEM images display uniform MC fibril distribution. The accompanying selected area electron diffraction (SAED) pattern presents HA crystals’ concentric rings, indicating a dominant alignment along the (002) plane. High-magnification TEM and fast Fourier transform (FFT) analysis evidence crystalline and amorphous calcium phosphate coexistence. (**D**) Scanning transmission electron microscopy (STEM) images of thin foils from a single sublayer, obtained through the focused ion beam (FIB) technique, show parallel (upper) and perpendicular (lower) fiber orientations. (**E**) Scanning electron microscopy (SEM) images reveal the ALB’s fracture surfaces displaying a rotating layer pattern akin to plywood. (**F**) The synthetic ALB sample demonstrates morphological similarities to natural cortical bone, as seen in the various shapes and sizes, with the fracture surface exhibiting a lamellar structure, as shown on the right. Reprinted with permission from Ref. [226], Copyright 2023, Authors. CC-BY.

Multi-directional ordered-structural arrangements can be seen in addition to layered structures in various natural materials, including tooth enamel, bone, muscle, and tendon. Natural materials have outstanding strength, toughness, and impact resistance thanks to these structures, which span the nanoscale to the macroscale. Bones, teeth, tendons, and ligaments are frequently replaced or repaired using biomedical materials that mimic these components. With a 96 wt% inorganic component, tooth enamel is a highly mineralized structure renowned for its extreme durability. It has been utilized as a bionic template to create materials for dental restoration. Notably, a sort of ceramic that resembles enamel was made by directing the growth of TiO_2_ nanorods on a tin oxide substrate that had been doped [223]. Using layers of HA and ZrO_2_, Zhao et al. developed a multiscale assembly process to produce bulk dental enamel [224]. Artificial tooth enamel (ATE) was developed, and it demonstrated exceptional levels of toughness, strength, and hardness, making it a suitable material for dental restoration. Type I collagen and hydroxyapatite nanocrystals combine to form mineralized collagen fibers in bone, another highly inorganic tissue. The basic network of the bone is made up of these fibers, which are grouped periodically. In the realm of biomaterials, materials that resemble bone are a popular issue. Through protein-induced intrafibrillar mineralization of cell-laden collagen, Thrivikraman et al. created a scaffold that resembles bone, offering a model system for studying bone physiology and disease [225]. Furthermore, utilizing a multiscale cascade regulation technique, Zhao et al. designed a collagen/HA artificial lamellar bone (ALB) that is centimeter-sized and has mechanical characteristics close to real bone [226]. The successful development of a centimeter-sized artificial lamellar bone has been achieved for the first time by implementing a meticulously coordinated strategy known as “multiscale cascade regulation.” This approach utilizes molecular self-assembly, electrospinning, and pressure-induced fusion methodologies implemented across multiple scales ranging from the molecular to the macroscopic (Figure 7). The artificial lamellar bone, predominantly composed of mineralized collagen fibrils organized hierarchically, successfully replicates the chemical composition, multiscale structural arrangement, and rotated plywood-like structure observed in natural lamellae. Notably, this achievement is accomplished without the incorporation of any synthetic polymer. Consequently, it presents a distinctive amalgamation of possessing a low weight and exhibiting a high degree of stiffness (Ey ≈ 15.2 GPa), strength (σf ≈ 118.4 MPa), and toughness (KJC ≈ 9.3 MPa m^1/2^). Implementing a multiscale cascade regulation strategy effectively addresses the limitations associated with individual techniques, enabling the development of advanced composite materials. This approach facilitates the precise control of hierarchical structural organizations at multiple scales, enhancing mechanical properties.The fabricated artificial lamellar bone (ALB) brilliantly mirrors the structural intricacies found in natural lamellar bone. It emulates the intricate hierarchical organization of mineralized collagen (MC) microfibrils, ranging from the nanoscale to the macroscale (Figure 8A). At the smallest scale, mineralized collagen microfibrils, each approximately 8 nm in diameter, form progressively through a biomimetic mineralization process. This process starts with nucleating amorphous calcium phosphate (ACP) precursors on the pre-constructed collagen microfibrils. The ACP transitions to crystalline apatite, simultaneously elevating the order of MC microfibrils. Transmission electron microscopy (TEM) images and fast Fourier transform (FFT) patterns give credence to the in situ co-assembly of nHAp and collagen microfibrils. Furthermore, the SAED patterns display diffraction rings characteristic of nHAp, mapped to the (002) and (211) crystallographic planes (Figure 8B). The electrospun MC fibrils, mimicking the spontaneous assembly seen in nature, have a consistent diameter of about a hundred nanometers, setting the stage for organization at more intricate levels (Figure 8C). Distributed uniformly within the electrospun MC fibrils, these MC microfibrils orientate along the fibril’s length, directed by the strong electric field and shear force during electrospinning. The SAED pattern further emphasizes the polycrystalline nature of HAp, showcasing a (002) preferred orientation parallel to the collagen fibrils (Figure 8C insert). These electrospun MC fibrils accumulate in layers, either in aligned or random formations, resembling the pervasive structures in natural bones. Layer upon layer of aligned MC fibrils, adjusting by 30° each time, replicate a lamellar unit, eventually compacting into a bulk bone that retains the initial orientations and structures post pressure fusion (Figure 8E,F). Supplementing this, the focused ion beam (FIB) produced thin foils, which, when observed through scanning transmission electron microscopy (STEM), confirmed the maintained orientation of MC microfibrils and fibrils (Figure 8D). Lastly, the resulting ALB strikingly resembles the natural cortical bone, with its fracture surface showcasing a tightly packed lamellar-like microstructure (Figure 8F).

#### 6.1.2. Inspired by Surface Structure

In conjunction with their inherent internal composition, the distinctive surface architectures of natural substances frequently play a substantial role in endowing them with exceptional characteristics, including enhanced wetting behavior, adhesive properties, and tactile perception capabilities. The external structures exhibit novel approaches in the development of functional biomedical materials. The superhydrophobic and adhesive surface structures could inspire synthetic nanomaterial routes.

Numerous natural creatures, including lotus leaves, red rose petals, mosquito complex eyes, butterfly wings, and water strider legs, exhibit superhydrophobicity, a unique wetting feature with a contact angle higher than 150 degrees. It has been demonstrated to possess antimicrobial qualities. The nanoarray surface structure of cicada wings, which kills bacteria in three minutes, was used by Ivanova et al. [227]. Using a reactive-ion etching process, the scientists also created black silicon with nano-protrusions modeled after dragonfly wings. This bio-inspired surface structure demonstrated strong antibacterial efficacy against Gram-positive and Gram-negative bacteria. To reduce the contact area and minimize biofouling, the lotus leaf’s superhydrophobic feature, which results from hierarchical architectures of microsized protrusions and nanosized wax tubules, induces self-cleaning [228]. With over 98% bactericidal effectiveness against Escherichia coli, a team led by Jiang successfully developed a hydrophobic surface inspired by lotus leaves [229]. Li and colleagues used straightforward dip-coating techniques to design functional gauzes by coating regular gauze with a PDA hydrophobic layer and depositing Ag NPs to obtain a shape resembling a lotus leaf. In vivo experiments revealed that this has effective anti-adhesion properties, reducing wound adhesion and harming skin less when removed [230].

**Figure 9:**
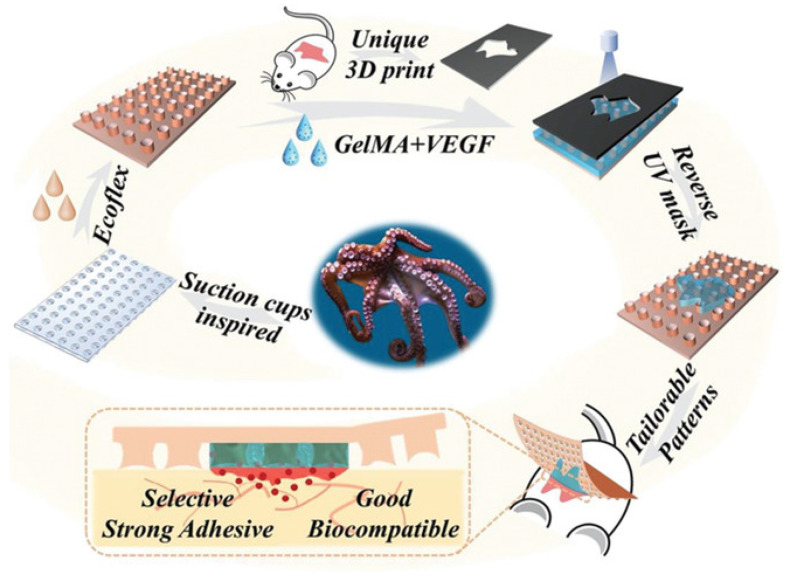
Schematic illustration of a biomimetic, skin-adhesive patch with customizable wound coverage. The distinctive GelMA-VEGF dressing, tailored to a specific wound shape, is developed on the surface of the Ecoflex patch by incorporating a UV mask. Reprinted with permission from Ref. [232], Copyright 2021, Authors.

**Figure 10:**
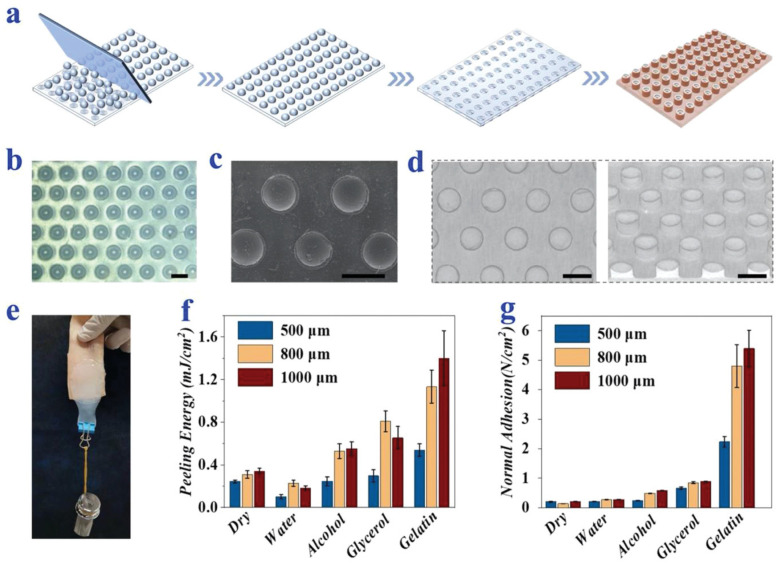
Creating and evaluating the adhesive capacity of the biomimetic patches. (**a**) An illustrative representation of the production procedure of the biomimetic patch. (**b**,**c**) Visual (**b**) and scanning electron microscopy (**c**) images showcasing the suction cups (measuring 800 μm in diameter). (**d**) Micro-CT scans of the suction cups (800 μm diameter) viewed from above and from the front. All accompanying scale bars represent 1000 μm. (**e**) An image capturing the adhesive patch adhered to pig skin, effectively bearing a weight of 0.2 kg. (**f**,**g**) Graphs indicating the resistance to peeling (**f**) and the vertical detachment and (**g**) forces of suction cups of three varied sizes when tested on both dry and damp surfaces. Reprinted with permission from Ref. [232], Copyright 2021, Authors.

Through billions of years of evolution, several creatures, including the gecko, tree frog, and octopus, have acquired incredible sticky skills. Due to their hierarchical microstructures, which allow their toe pads to adhere to various surfaces, geckos have an exceptional capacity to attach to surfaces [231]. The suction cups with protuberances on an octopus’ tentacles give them their adhesive properties, and tree frogs’ toe pads are made of closely packed nanopillars and tightly arranged epithelial cells. To provide good adhesion to dry and wet surfaces, Huang et al. developed a wound patch inspired by octopi’s adhesive structure [232]. The team has developed a biocompatible wound patch with a targeted design and selective stickiness by combining template replication and mask-guided lithography. Figure 9 depicts a biomimetic, skin-adhesive patch with customizable wound coverage. The distinctive GelMA-VEGF dressing, tailored to a specific wound shape, is created on the Ecoflex patch’s surface by incorporating a UV mask. For adhesion to healthy skin, these patches use an Ecoflex film with microstructures that resemble the suction-cup effect, and for contact with the wound, they use a biocompatible gelatin methacryloyl (GelMA) hydrogel. Using a flexible ultraviolet mask, the GelMA hydrogel is customized to the geometry of each unique wound location, combining adhesion and non-adhesion properties into a single patch. Vascular endothelial growth factor is also included in the patch to hasten recovery. As the authors demonstrate, these characteristics enable the patches to adhere to different skin types and improve the healing of a rat skin wound model. The limitations of conventional patches are thus anticipated to be solved by this adaptable patch, making it an attractive option for wound healing and associated biomedical uses. A gravity-driven self-assembly technique allowed monodispersed steel microspheres to settle into the microcolumns. A wiper blade ensured each microsphere was properly placed and secured. The microspheres were designed to have a radius slightly bigger than the cavity’s, enabling the creation of a negative mold with a convex bottom (Figure 10a). The yet-to-be-cured Ecoflex liquid precursor was poured over the mold’s surface, filling the microcolumns that had convex bottoms. The micro-suction-cup array was finally produced after vacuum degassing, heat curing, and removal from the mold (Figure 10b–d). It is important to highlight that by altering the size of the negative mold, different-sized Ecoflex patches could be produced, showcasing the method’s adaptability. When a vacuum is generated within the cavity, molecules at the boundary will restrict external liquids from entering, aiding in preserving the vacuum. This phenomenon becomes more pronounced on textured surfaces like pig skin. The research group tested four commonly used clinical liquids (water, ethanol, glycerin, and gelatin) for interface wetting. The outcomes revealed that the Ecoflex patch displayed exceptional adhesion on damp surfaces, holding up to a 0.2 kg weight in a tangential manner when stuck vertically to moist pig skin (Figure 10e). The standard adhesion and peel strength data further supported this observation (Figure 10f,g).

**Figure 11:**
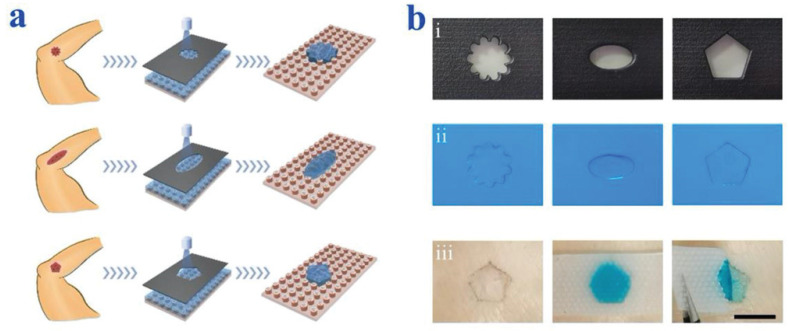
Creating GelMA dressings with customizable shapes. (**a**) Diagrams illustrating the process of creating GelMA dressings with adjustable shapes. (**b**) (**i**) Various shapes of UV masks produced through 3D printing. (**ii**) Creating GelMA hydrogels with shapes tailored to fit the UV masks. (**iii**) The GelMA hydrogel can accurately cover each pig skin wound area, and the contrasting properties of adhesion and anti-adhesion are incorporated within the same patch film. Blue dye was included in the GelMA solution for enhanced imaging. The scale bar indicates 1000 µm. Reprinted with permission from Ref. [232], Copyright 2021, Authors.

GelMA hydrogels with unique shapes were produced using mask lithography on Ecoflex patches, as depicted in Figure 11a. In particular, a UV mask was fabricated initially using 3D printing technology to conform to the distinct contours of individual wounds. The purpose of this mask was to partially impede the transmission of UV light while allowing other portions to pass through. Following that, a solution of GelMA was administered onto the surface of the Ecoflex patch and subsequently concealed with the mask. Consequently, when subjected to ultraviolet (UV) light, solely the pregel solution located in the specific regions exposed to UV radiation would undergo polymerization, forming a solid hydrogel.

In contrast, the portions of the solution that were not exposed to UV light would remain in a liquid state and could subsequently be removed by wiping. The methodology resulted in developing customized hydrogel dressings to fit the diverse contours of UV masks, as depicted in Figure 11b. In addition, GelMA hydrogels of different concentrations were prepared, and their efficacy in generating distinct geometries was assessed. The results demonstrated that GelMA hydrogels with varying pregel concentrations (10, 15, and 20 wt%) exhibited high efficacy and reliability in achieving distinct geometries.

The hierarchical topologies of tree frog toe pads and the octopus suction-cup structure inspired Kim et al.’s development of a skin patch, which demonstrated increased peeling resistance and enhanced adhesion [233]. These biomimetic methods influence how sticky biomaterials are created for dry and moist environments. The preparation of materials with unique performances for various healthcare needs has been made easier by biomimicry. For example, high-strength NPs inspired by nacre are used for orthopedic implant materials, and hydrophobic surfaces inspired by lotus leaves are used for antibacterial purposes. Biomimetic structures have been created in artificial materials using a variety of production processes, including freeze casting, plasma etching, and self-assembly. To fully realize the potential of biomimetic nanomaterials in the biomedical field, additional research is required to address issues like scale-up fabrication, accurately reproducing the structures or properties of natural materials, and understanding the connection between bio-inspired structures and biological properties.

### 6.2. Inspired by Natural Biomolecule

Biomolecules serve as the essential building blocks of living organisms, fulfilling crucial functions in various physiological processes, including metabolism, transmission of genetic information, and immune responses, among other vital activities. Given the progress made in medical standards and the increasing demand for treatments, there is an urgent requirement to develop safe and efficient biomaterials. These biomaterials are crucial for various applications, such as targeted drug delivery and regenerative medicine. The utilization of biomimicry principles in the replication of biomolecules presents a viable method for the development and production of biomaterials with exceptional performance characteristics. This methodology enables the transformation of chemical compounds into artificial materials that imitate the composition and behavior of naturally occurring biomolecules, thereby meeting the rigorous requirements of biomedical uses. This section provides an overview of various biomedical nanomaterials that draw inspiration from biomolecules, encompassing their synthesis techniques and applications in biomedicine.

#### 6.2.1. Protein-Inspired Nanomaterials

Proteins are essential to all living things and are involved in many biological processes, structure, and function preservation. Scientists have attempted to mimic the sticky surface structure of natural creatures to overcome the difficulty of obtaining strong adhesion in wet interfaces. For instance, mussels use the amino acid 3,4-dihydroxyphenylalanine (DOPA) to secrete their adhesive protein to cling to submerged surfaces. DOPA’s catechol group promotes robust adherence in a wet environment. In response, Gan et al. created polydopamine (PDA), which has a molecular structure comparable to DOPA and is rich in catechol groups, to create antibacterial and sticky hydrogels for wound healing. This method increased the bacterial hydrogel’s ability to adhere to surfaces, strengthening the sterilizing effect [234]. The hydrogels also showed a strong affinity with cells and tissues, which sped up the healing process. Barnacle cement proteins can also create potent hydrophobic and cation interactions that strengthen cohesion. Ni et al. created modified polyphosphazene/polyvinyl alcohol tissue adhesive hydrogels using this model. These demonstrated a potent adhesive force to tissue surfaces in aqueous conditions while avoiding the oxidation frequently associated with catechol-based hydrogels [235]. Additionally, the hydrogels favorably affected hemostasis and skin wound recovery, indicating a possible application in treating urgent wounds.

Bone and tooth proteins are essential to the biomineralization process. Notably, the tooth protein amelogenin directs the synthesis of HA, which is necessary for tooth enamel. Based on these findings, Chang et al. created a bioactive peptide to restore tooth enamel through biomimetic mineralization using low-complexity protein segments (LCPSs) found in the “fused in sarcoma” protein [236]. The LCPSs allowed amorphous calcium phosphate to convert more slowly into hydroxyapatite, producing a more integrated mineral structure and stronger enamel layers. This study offers a useful strategy for tooth enamel remineralization. Enzymes, primarily proteins, also catalyze biochemical events in the body and are crucial for detecting and managing diseases. The development of sophisticated artificial enzymes holds great promise for biomedicine. For instance, by incorporating MoO_3_ into a metal-organic framework, Yang et al. created a bio-inspired spiky peroxidase-mimic enzyme to catch and kill germs. It has the potential for use in tissue engineering [237]. Additionally, Zhang et al. created a method to quickly gelate injectable hydrogels to fill tissue defects [238]. Glucose oxidase and ferrous glycine (Fe[Gly]_2_), an enzyme complex inspired by biological systems, were used in this procedure. The ensuing reactions produced carbon free radicals, which facilitated quick polymerization. These hydrogels demonstrated adequate mechanical stability and an excellent capability for cartilage regeneration, making them viable fillers for cartilage repair.

#### 6.2.2. Peptide-Inspired Nanomaterials

Short sequences of amino acids known as peptides are essential for controlling hormone release and metabolism. The design and manufacturing of peptide-inspired nanomaterials, which have several uses in the biomedical industry, have advanced significantly in recent years. Due to their specificities, peptide-inspired nanomaterials can be used as targeting agents. For instance, Yang et al. created the biomimetic peptide R4F with Apolipoprotein A-I as inspiration to target the SR-B1 receptors on M1 macrophages in rheumatoid arthritis cases [239]. This peptide was subsequently altered to coat an anti-inflammatory medication on a neutrophil membrane-wrapped F127 polymer, drastically reducing M1 macrophage polarization and increasing M2 macrophage polarization, suggesting successful rheumatoid arthritis treatment. Nanomaterials inspired by peptides are also widely used in cancer treatment. They enable the precise delivery of nanomedicines or photosensitizers to tumor locations. Under particular light wavelengths, these photosensitizers produce heat or reactive oxygen, which causes the death of tumor cells. This tactic works well and has fewer negative consequences.

In the antibacterial field, peptide-inspired nanomaterials are also promising. For instance, to combat methicillin-resistant Staphylococcus aureus (MRSA), Xie et al. prepared an antibacterial peptoid polymer based on host-defense peptides [240]. After prolonged use, this polymer showed outstanding anti-infection performance without producing drug resistance and reduced the growth of MRSA biofilms. In addition, tissue restoration has been performed with nanomaterials inspired by peptides. Self-assembled antimicrobial-antioxidative peptides enhancing infected wound healing in vivo have been the subject of certain studies. Another study described the usage of a bone-healing scaffold as a method for repairing bone defects in the presence of infection. This scaffold was made by altering an antibacterial peptide and an osteogenic growth peptide on a polyetheretherketone surface.

#### 6.2.3. Lipid-Inspired Nanomaterials

Lipids, organic substances found in the body in structures like vesicles and cell membranes, are essential for energy storage and chemical communication among cells, tissues, and organs. As a result, the biomedical industry makes substantial use of lipid-inspired nanomaterials. Cell membranes, mostly made of lipids and proteins based on fatty acids, can isolate cells from their environment and regulate the flow of nutrients and waste products. They can transport NPs or medicines directly. For instance, Ying et al. improved tumor targeting effectiveness by encapsulating nanocamptothecin into macrophage membranes [241]. These macrophage-mimicking nanocamptothecin particles accumulated more at tumor sites than uncoated NPs in a mouse model of breast cancer. Additionally, nanocarriers coated with erythrocyte membranes were employed for in vivo biological imaging and medication delivery since they could resist macrophage clearance. Similarly, functional NPs were encapsulated in platelet membranes, reducing macrophage-like cell absorption and demonstrating preferential adherence to human blood vessel injury.

Vesicles, composed of lipid bilayers, store, digest, or transport materials. Recently, hybrid bio-inspired nanovesicles were developed for lung tissue transport and immunomodulatory activity by fusing lung-targeting liposomes and macrophage-derived nanovesicles. Extracellular vesicle-based collaborative anti-infective therapy was the subject of a straightforward biomimetic technique by Qiao et al. [242]. Pd-Pt nanosheets and ginger-derived extracellular vesicles gave EVs-Pd-Pt NPs good biocompatibility and sustained blood circulation, producing a potent antibacterial effect. Extracellular vesicles (EVs) have also been employed to encourage the differentiation of stem cells into several adult somatic cell types. In a recent method, EVs stimulate fibroblasts to transdifferentiate into cells that resemble induced cardiomyocytes. Direct conversion of embryonic fibroblasts toward mature induced cardiomyocytes may be accelerated using EVs generated from embryonic stem cells during cardiomyocyte differentiation. The electrical and typical cardiac calcium transient properties of these generated cardiomyocytes were similar to those of cardiomyocytes.

#### 6.2.4. Other Biomolecule-Inspired Nanomaterials

Biomolecules outside proteins and lipids, such as viruses and saccharides, influence biomedical nanomaterial design. Complexes of saccharides and proteins are essential for biological processes like immunological control and drug transport. Taking this as a foundation, Duan et al. created a branched glycosyl polymer-pyropheophorbide-a conjugate that may be used as a drug delivery system [243]. Anticoagulants like heparin, a highly sulfated glycosaminoglycan produced by mast cells, are frequently utilized. Sodium alginate was used as a biological macromolecule model by Ma et al. to build a heparin-like anticoagulant biomolecule to reproduce its function. This biomolecule demonstrated good anticoagulant efficacy and biocompatibility in vitro and in vivo [244]. To develop a new design methodology for anticoagulant surfaces, Wang et al. explored the endothelialization of a novel heparin-like polymer [245]. Viruses, contagious organisms that multiply inside live cells, can be bionic models for artificial materials. Chen et al. developed a reversible and activatable near-infrared II nanoprobe by imitating a virus. This might help with future viral encephalitis interventions by tracking the progression of viral infection in real time [246]. In a different investigation, Li et al. created a nanodrug inspired by a virus that might avoid lysosomal hydrolysis while being delivered, potentially providing a unique method for treating tumors [247].

Nanomaterials with biomolecular inspiration have demonstrated promise in tumor therapy, medication transport, and tissue engineering. They increase the effectiveness of treatment, lessen adverse effects, and are frequently artificially produced, ensuring a plentiful supply of raw ingredients with strict quality control. Making multifunctional BINMs with intricate architectures resembling natural proteins is still difficult. These NPs also need to have their long-term safety, immunogenicity, and in vivo stability investigated. Emerging technologies like artificial intelligence may help the development of these nanomaterials in the future by providing fresh perspectives for individualized and targeted design [248].

### 6.3. Bioprocess-Inspired Nanomaterials

The bioprocess can efficiently and accurately construct hierarchical structures or synthesize essential substances in a natural environment while operating under mild conditions. The remarkable bioprocesses offer environmentally sustainable methods for the production of synthetic materials. The acquisition of knowledge regarding natural bioprocesses has facilitated the development and utilization of advanced synthesis technologies in creating high-performance biomaterials.

#### 6.3.1. Inspired by Biomineralization Process

The production of organic–inorganic composites in structures like bone and teeth, known as biomineralization, inspires the creation of biomedical materials [1,249,250,251]. Researchers have used this procedure to produce materials for cancer therapy, medication creation, and hard tissue repair. By simulating the mineralization process, Li et al. created a dental replacement with enamel’s mechanical qualities [252]. Tang et al. suggested a method for restoring enamel using a solution of calcium phosphate ion oligomers [253]. Zhou et al. produced extremely rigid DNA-HA bulk composites for dental applications using an engineering mineralization method [254]. Enhancing bone healing materials has also relied heavily on the biomineralization process. Bioactive nanominerals can be added to bone-repairing scaffolds using methods inspired by biomineralization to promote osteogenic activity. To better understand the process of bone formation, Ping et al. produced strontium carbonate crystals inside collagen fibers [255]. New cancer treatment approaches are also inspired by biomineralization. Zhao et al. suggested exploiting the buildup of calcium salts, which causes cell calcification and cancer cell death, as a drug-free tumor treatment method [256]. A macromolecular medication that causes cancer cells to calcify extracellularly was created through other studies. Last, a biomineralization-inspired technique was applied to safeguard tumor-targeted delivery carriers, highlighting its applicability in numerous biomedical research fields.

#### 6.3.2. Inspired by Photosynthesis

Advanced artificial photosynthetic systems for biomedical materials have been developed due to photosynthesis, a crucial bioprocess found in plants, algae, and cyanobacteria. Microalgae, particularly cyanobacteria, have recently been used as effective oxygen sources to combat tumor hypoxia. For instance, Huo et al. produced oxygen using a hybrid of cyanobacteria and photosensitizers, which, when exposed to laser irradiation, transformed into singlet oxygen and killed cancer cells [257]. In addition to supplying oxygen for wound healing, microalgae also promote angiogenesis and collagen synthesis [258]. Artificial photosynthesis systems, like the one created by Chen et al. using spinach nano-thylakoid units (NTUs), show promise for degenerative disorders like osteoarthritis. The development of CM-NTUs, which combine NTUs with the chondrocyte membrane (CM), improved intracellular ATP and NADPH levels, enhancing cell metabolism and delaying the onset of osteoarthritis [259]. Nanomaterials with inspiration from biomineralization processes are widely used in biomedical disciplines and can safeguard the environment and promote the sustainable development of biomaterials. The inhomogeneous conveyance of mineralized media, slow reaction speeds, and difficulty in creating substantial, structurally sound structures continue to be problems. Like photosynthesis-inspired solutions that offer novel disease treatments, they are still in the early phases. They must fully address issues with light penetration into tissue, immunological responses, and biological safety.

### 6.4. Challenges for Bio-Inspired Synthesis

To improve performance in biomedical applications, bio-inspired design efficiently transfers special features and functionalities from natural materials to synthetic materials. Natural materials’ hierarchical systems and individual components can be studied to provide solutions to various biomedical problems. The idea of manufacturing inspired by biological processes has evolved, enabling the creation of biomedical materials under benign circumstances. Designing biomaterials for various medical applications is made possible by certain biomimetic techniques. For instance, biomimetic nanovesicles are used for medicine administration, whereas bio-inspired layered nanostructures improve the mechanical qualities of bone implants. Biomedical nanomaterials inspired by biomineralization and photosynthesis also show great potential for treating cancer and tissue engineering.

Although notable advancements have been achieved in bio-inspired biomedical nanomaterials, unresolved obstacles still necessitate attention to facilitate their effective implementation. These materials frequently require optimal performance in fluidic environments characterized by dynamic flow, such as bodily fluids, where their properties may undergo compromise relative to their performance in dry or stabilized conditions. The occurrence of mechanical failure in moist environments is a prevalent concern. One of the primary obstacles encountered in the field is expanding the production of BINMs. This is due to the increasing complexity associated with achieving precise arrangement of structures and components at the nanoscale, which consequently restricts the size and yield of these materials. There is currently a deficiency in cost-effective and efficient techniques for producing goods on a large scale. Furthermore, it is imperative to conduct extensive research and analysis on the biological characteristics of BINMs. This is crucial because numerous materials with potential applications have not undergone thorough in vitro and in vivo assessments, which poses a significant obstacle to their successful implementation in clinical settings.

To enhance the efficacy of the development process for multifunctional biomedical nanomaterials, it is imperative to incorporate advanced methodologies such as integrating multiple bio-inspired strategies and utilizing computational simulations. Moreover, it is important to note that the study of biomedical materials encompasses various interdisciplinary domains, including material science, biology, and medicine. Consequently, the comprehensive assessment of BINMs necessitates the collaborative efforts of researchers specializing in materials, biologists, and medical professionals. Collaboration is pivotal in facilitating the effective translation of materials from the laboratory setting to clinical applications.

## 7. Design Principles of Bio-inspired Nanomaterials’ Interfaces

Recent interest in research on bio-inspired interfaces has increased as a result of its expanding significance in the field of biomedical science. Enhancing medicine effectiveness while reducing adverse effects is one of the main goals in this sector. This includes increasing the effectiveness of distribution systems and giving targeting abilities more specificity. Another important factor is biomedical imaging, which provides essential information about molecular distributions in vitro and in vivo. The use of NPs in biomedical applications has been extensively explored due to their adaptability and accessibility [260]. When designing a delivery method, it is critical to consider the NPs’ trajectory through the body and any potential roadblocks on the way to the illness site. NP-based delivery techniques typically include injection or oral consumption to penetrate the circulatory system.

**Figure 12:**
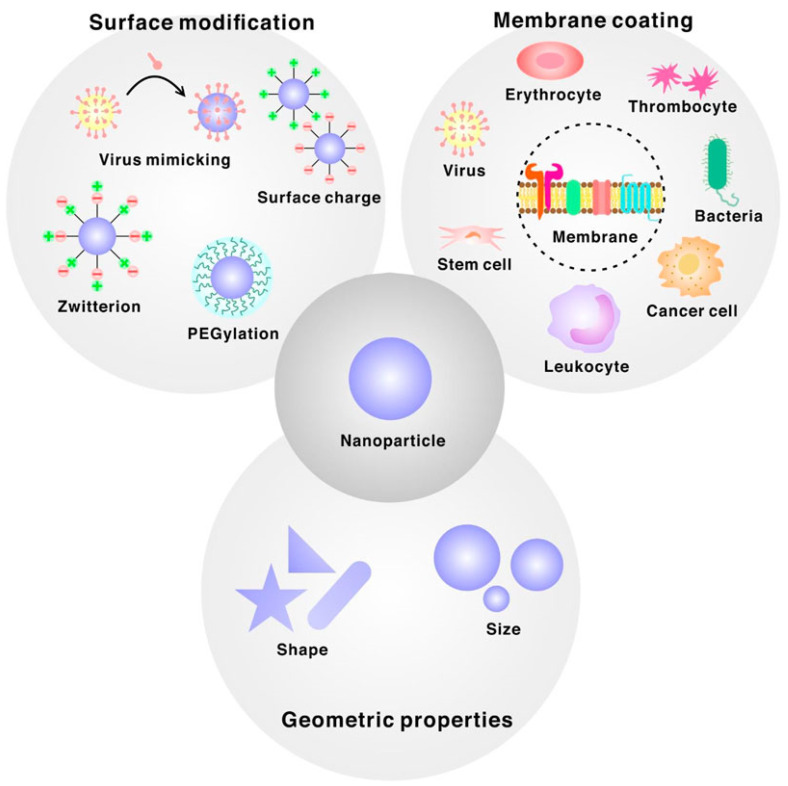
Three distinct categories can be drawn from the design guidelines for NP interfaces inspired by biological systems. First, membrane-coated NPs are produced using components like mammalian cells, cancer cells, bacteria, or viruses. The second category includes ligands that modify surfaces. The third category of design concepts for these bio-inspired interfaces is the alteration of the geometric aspects of the NPs, such as their size or shape. Reprinted with permission from Ref. [260]. Copyright 2022, Authors (CC-BY).

Consequently, ensuring that the NPs reach the intended organs without being eliminated by the body is a crucial component of effective delivery. The reticuloendothelial system (RES), which comprises the liver and spleen, eliminates NPs from the circulatory system. When NPs are administered intravenously, they are frequently labeled as alien substances and go through hepatic Kupffer cell sequestration [261]. An additional issue emerges when NPs interact with various biomolecules, including plasma proteins, upon entering a complex biological milieu like blood, interstitial fluid, or the extracellular matrix, creating a protein corona. This corona development activates the mononuclear phagocyte system (MPS) for quick ejection as proteins build up on the NP surfaces [262]. To overcome these obstacles, NPs must have the proper mechanisms that will allow them to safely pass through the body and arrive at their target without being prematurely removed. One common technique to prevent RES clearance and early ejection is to cover NPs with the membranes of circulating blood cells [263]. Providing enough time in the circulatory system is crucial for the designed NPs to reach their target site. The capacity of NPs for selective targeting, or the capacity to interact just with the area of interest while disregarding other sites (cells, tissues, etc.), is another key aspect of NPs. NPs must also be securely cleared from the body when the task is completed without having any negative effects. They are essential for creating NPs since they ultimately dictate how the particles will behave when ingested by the body. Given its complexity as a biological environment, the human body can vary significantly from person to person. Designing NPs that can overcome these obstacles while keeping their intended functionality becomes even more difficult and complex. Figure 12 illustrates three distinct categories that can be drawn from the design guidelines for NP interfaces inspired by biological systems. First, membrane-coated NPs are produced using components like mammalian cells, cancer cells, bacteria, or viruses. The second category includes ligands that modify surfaces. This includes altering the NPs’ surface using polyethylene glycol (PEG), zwitterions, positively or negatively charged ligands, or viral capsids. The third category of design concepts for these bio-inspired interfaces is the alteration of the geometric aspects of the NPs, such as their size or shape.

**Figure 13:**
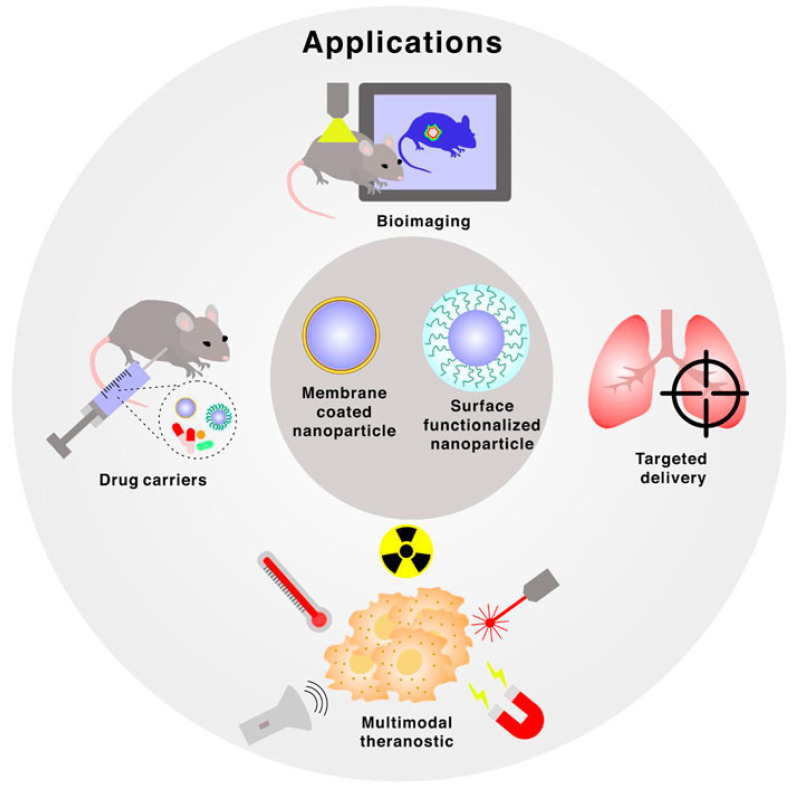
Membrane-coated and surface-functionalized NP applications include drug delivery, multimodal theranostic, and bioimaging. Reprinted with permission from Ref. [260]. Copyright 2022, Authors (CC-BY).

Researchers are interested in bio-inspired NPs because of the wide range of biomedical science applications they could have, including targeted drug administration, in vivo therapies, bioimaging, and cancer treatment. However, the human body’s capacity for recognizing and getting rid of foreign compounds is one of the main obstacles to overcome. For example, the mononuclear phagocyte system (MPS) is quickly expelled from the body in response to opsonin interactions. The targeting abilities of ligand-functionalized NPs might also be lost due to excessive protein corona formation, which can also significantly affect the surface chemistry of NPs, negating or changing their desirable features. Without further surface alterations, NPs rely solely on the improved permeability retention effect, which is typically ineffective [264]. Numerous methods have been developed to address these problems by changing the way NPs function or their geometrical characteristics. These techniques are divided into various groups in the following sections based on the type of alterations. Applications of membrane-coated and surface-functionalized NPs span a broad spectrum of fields (Figure 13). One of these is bioimaging, which uses NPs to improve visual data at the cellular or molecular level. NPs can be used in targeted delivery to deliver medications to illness locations directly, boosting therapy effectiveness and lowering side effects. In multimodal theranostics, a discipline that integrates diagnostics and therapies, NPs are also employed to diagnose and treat diseases simultaneously. Finally, these NPs are used as drug carriers, acting as a means of controlled and precise drug delivery.

### 7.1. Biomimetic Functionalization of BINPs

**Figure 14:**
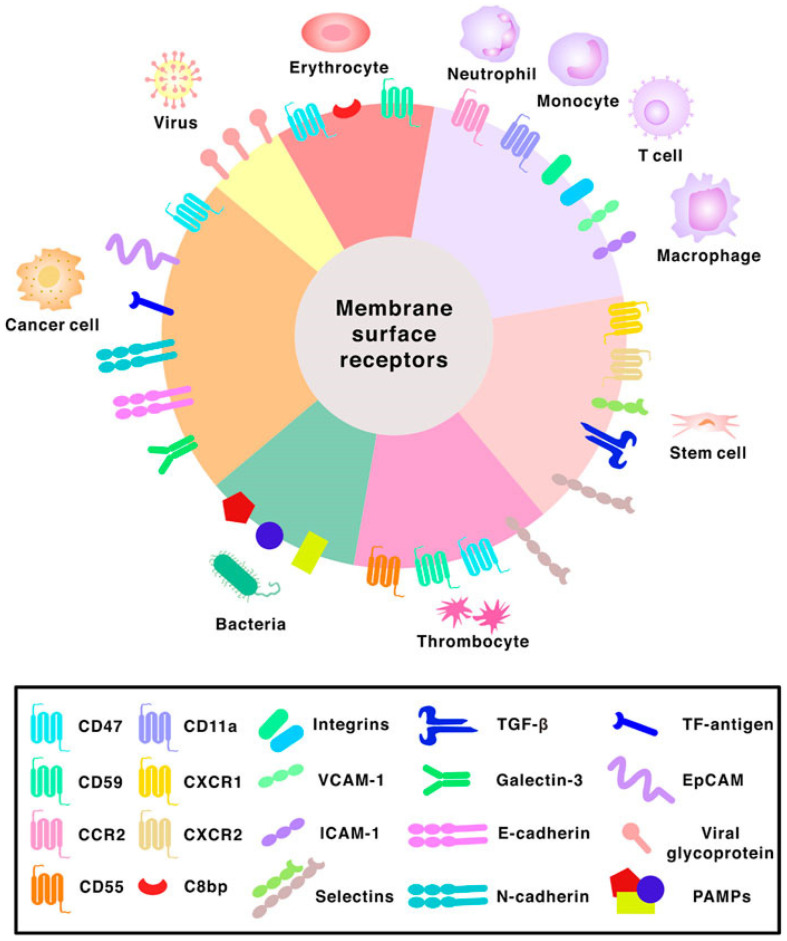
Various membranes host a diverse array of surface receptors. The abbreviations are listed below: CCR2 is an acronym for C-C chemokine receptor 2, CXCR1 represents C-X-C chemokine receptor 1, and CXCR2 denotes C-X-C chemokine receptor 2. C8bp is an abbreviation for C8 binding protein, VCAM-1 signifies vascular cell adhesion molecule-1, and ICAM-1 refers to intercellular adhesion molecule-1. TGF-β corresponds to transforming growth factor beta, TF-antigen refers to Thomsen–Friedenreich antigen, EpCAM stands for epithelial cell adhesion molecule, and PAMPs is the abbreviation for pathogen-associated molecular patterns. Reprinted with permission from Ref. [260]. Copyright 2022, Authors (CC-BY).

Biomimetic substances can replicate or imitate the attributes, composition, chemical properties, and functionalities of their biological counterparts [265]. Both red and white blood cells can traverse the circulatory system without premature elimination by the mononuclear phagocyte system (MPS). Platelets can resist phagocytic activity and are equipped with surface receptors that enable them to selectively target specific sites, thereby aiding in tissue repair following an injury. Cancer and stem cells have also been investigated in this field of study (Figure 14). The emergence of membrane-coated nanoparticles (MCNPs) has resulted from the need to prolong circulation time upon entry into the bloodstream, enable precise drug delivery within the body, or function as contrast agents for bioimaging applications [266]. Moreover, scholarly investigations have been undertaken on materials derived from pathogens, including bacteria [267] and viruses [268], which exhibit distinctive capacities in the delivery of payloads. Consequently, it is crucial to comprehend the unique characteristics of different biological entities and exploit their therapeutic capabilities. This section examines the utilization of the intrinsic characteristics of biological materials by scientists to develop NPs for biomedical purposes while also addressing the potential obstacles they may face during this process.

#### 7.1.1. Erythrocyte Membrane

Red blood cells (RBCs) are the predominant cellular constituents within the human body. They possess notable attributes such as biocompatibility, prolonged circulation duration, and biodegradability, rendering them highly suitable for utilization as carriers of NPs. The CD47 proteins, which serve as markers of self, are found on the surfaces of red blood cells (RBCs) and play a role in preventing their phagocytosis by immune cells. As a result, the presence of these proteins leads to an extended circulation time for RBCs [269]. This characteristic has been employed to enhance drug delivery systems, wherein NPs are enveloped with red blood cell membranes (RBCMs), forming RBCM-NPs. The successful generation of RBCM-NPs has been achieved by encapsulating poly (lactic-co-glycolic acid) (PLGA) NPs within RBCMs. Applying this coating facilitates a 64% reduction in macrophage uptake of the NPs and prolongs their elimination half-life [270]. RBCM-NPs have demonstrated efficacy in selectively targeting particular conditions, such as atherosclerotic plaques, and in cancer therapy.

Nevertheless, the effectiveness of RBCMs is dependent on the enhanced permeability and retention (EPR) effect, given its inherent absence of natural tumor-targeting capabilities. To augment the targeting efficacy, ligands are introduced into red blood cell membranes (RBCMs) through chemical synthesis or lipid insertion techniques [271]. These supplementary components have substantially improved their capacity to target cancer cells selectively. Furthermore, RBCM-NPs have demonstrated bioimaging practicality, particularly in in vivo tumor imaging. Ongoing investigations and advancements in dual-functionalized RBCM-NPs show encouraging outcomes in enhancing bioimaging capabilities.

#### 7.1.2. Leukocyte Membrane

Leukocytes, also known as white blood cells (WBCs), are important immune cells that protect the body from diseases and infections. WBC membrane-coated NPs able to self-recognize, penetrate biological barriers, and bind to receptors at disease locations have been developed due to their varied capabilities. For instance, WBC membrane-coated nanoporous silica particles that maintain WBC characteristics, such as avoiding the immune system and crossing the endothelium barrier, have been produced. Various WBCs, including neutrophils, macrophages, monocytes, and T cells, have been employed as NP carriers. By simulating the interaction of the cell with inflammatory tissues, neutrophil membrane-coated NPs, for example, can target and lower bacterial load at infection sites [272]. Due to their capacity to target inflammation and tumor endothelium specifically, macrophages have been incorporated into macrophage membrane-coated nanoparticles (MMCNPs) to slow the progression of atherosclerosis [273]. They also show promise in treating cancer and bioimaging, although they have drawbacks, such as the ability to target particular cancers [236,237] exclusively. The power of monocytes to infiltrate has been taken advantage of in a lipid NP-based drug-delivery platform, which enables monocytes to transport and deliver lipid NPs to sick areas [274]. Monocytes are normally recruited when physiological changes occur in the body. T cells have been employed to coat NPs’ membranes, increasing their circulation duration and enhancing cancer targeting. T cells have a higher concentration of targeting proteins than other WBCs. For instance, coated NPs with azide-modified T-cell membranes exhibit strong fluorescence intensity and an improved photothermal response. However, the lack of tumor-specific indicators displayed by solid tumor cells limits their ability to treat solid tumors [275].

#### 7.1.3. Thrombocyte Membrane

Blood components called platelets are in charge of starting blood clots when an artery is damaged. Their membranes’ distinctive qualities make them advantageous for coating NPs, as they can support active and passive drug targeting. Platelet membranes include CD47 “marker-of-self” proteins, similar to those found in red blood cell membranes, which resist immune clearance, extending the circulation of platelet membrane-coated nanoparticles (PMCNPs) for passive drug targeting [276,277]. Additionally, platelet membranes include particular surface receptors such as glycoprotein Ib that can connect to exposed collagen in injured vascular tissues to stimulate tissue repair or directly bind to pathogenic bacteria to enable active drug targeting [278,279]. They have become quite popular in the creation of nanotherapeutics as a result. Platelet-like proteoliposomes that strongly interact with circulating monocytes have been created to enhance post-infarction therapy. Because PM-NPs naturally include platelet surface proteins that can interact with anti-platelet antibodies, they can be used as an antibody decoy in treating immune thrombocytopenia [280]. Additionally, they are utilized in tumor imaging, drug delivery, and the detection of cancer cells. In a mouse model of human lung cancer, for instance, a study revealed that docetaxel-loaded PLGA NPs coated with platelet membranes preserved the maximum drug concentration in tumors, successfully reducing tumor growth. Additionally, PM-NPs have been used in developing phototheranostic nanoprobes to target various tumors and deliver additional anti-cancer medications actively. The immune system can remove PM-NPs from patients with autoimmune disorders when platelet autoantibodies combine with them to form immune complexes [281].

#### 7.1.4. Cancer Cell Membrane

Because they have surface receptors that permit adhesive contact, resulting in metastatic deposits, cancer cell adhesion molecules (CCAMs) play a significant role in cancer metastasis. Additionally, CD47 surface proteins are expressed by cancer cells, allowing them to avoid the immune system. Using these characteristics, researchers have coated NPs with cancer cell membranes (CCMs). CCM-coated NPs (CCM-NPs) can avoid immune detection and target malignant locations or tumors that are similar to them. They can be customized to satisfy the particular requirements of cancer therapy [282]. According to studies, compared to uncoated NPs and red blood cell membrane-coated NPs (RBCM-NPs), CCM-NPs have much higher cellular absorption and a strong affinity for source cancer cells [283]. CCM-NPs can achieve self-recognition, internalization by the source cancer cell lines, and highly selective targeting to the homologous tumor in vivo by adjusting the source of the cell membrane coating [284]. In tumor imaging, CCM-NPs have been widely employed and frequently functionalized with extra features [285,286]. For instance, recent research produced unique iridium compounds functionalized with black-titanium NPs coated with CCMs. These NPs have the potential to accumulate in malignant cells, accumulate in mitochondria, develop effective photothermal capability when exposed to NIR-II radiation, and form reactive oxygen species when exposed to ultrasonic radiation. This enables precise imaging of the tumor site and results in the elimination of tumor cells in mice models [287].

#### 7.1.5. Stem Cell Membrane

Mesenchymal stem cells (MSCs), extensively researched in biomedical research, are renowned for their simplicity in separation and capacity to target tumors. MSCs have been effectively used in medication delivery systems based on NPs. According to research, the loading of NPs into MSCs preserved cell viability and differentiation. A human glioma model also shows great selectivity for MSC membrane-coated NPs (MSCM-NPs) [288]. Numerous cancer-related research studies have used MSCM-NPs. Due to various chemical recognition moieties on the MSC membrane, an MSC membrane-coated gelatin nanogel, for instance, displayed excellent stability and tumor selectivity both in vitro and in vivo [289]. In an orthotopic breast cancer model, PLGA NPs covered with an MSC membrane demonstrated strong anti-tumor effectiveness [290]. Taking advantage of MSCs’ capacity for tumor homing, MSCM-NPs have been widely applied in bioimaging. A biocompatible MSCM-NP with multimodal imaging abilities for near-infrared fluorescence, magnetic resonance, and computed tomography has recently been developed [291].

#### 7.1.6. Bacterial Membrane

Despite being frequently harmful, some aspects of bacteria can be utilized in therapeutic settings. Immunogenic antigens and adjuvants in bacterial membranes activate innate immunity and support adaptive immunological responses. These can be carefully coated onto NPs to simulate how the immune system reacts to antigens naturally when germs are present. Bacterial outer membrane vesicles (OMVs) are typically coated on NPs to create bacterial membrane-coated NPs (BM-NPs) [292]. BM-NPs are a relatively recent development in cell membrane-coated NP research, yet they have a number of special benefits. They showed bacterial-specific targeting; for example, S. aureus-infected macrophages and organs were selectively targeted by PLGA NPs coated with S. aureus OMVs, a feature not seen in other membrane-coated NPs. Given the rapid rise of bacterial drug resistance, BM-NPs may be used to create antibacterial vaccines as an alternative to antibiotics [293]. For instance, BM-NPs were created utilizing OMVs from CRKP, which improved the survival probability of immunized mouse models when exposed to a lethal dose of CRKP. Because some bacteria naturally target tumors, BM-NPs can be used for cancer treatment or tumor imaging. BM-NPs have been used to create a cancer vaccine that, when paired with radiotherapy, demonstrated increased tumor growth inhibition and made anti-cancer immunological memory.

However, research on using bacterial membranes as NP coating agents is ongoing. The impact of OMV size on host cell entrance and the cytotoxicity of BM-NPs for realistic biomedical applications are among the difficulties. To minimize the inflammatory response of BM-NPs, lipopolysaccharide neutralizing peptides have been suggested as a partial answer; nonetheless, these issues must be uniformly resolved before BM-NP-based vaccines and treatments can go further.

#### 7.1.7. Virus-Derived Strategies

Viruses are frequently used in biomedical applications because they can protect and transfer nucleic acids into host cells while eluding the immune system. Adenoviruses and retroviruses have been utilized as viral gene vectors to introduce particular genes into host cells. Due to their pathogenicity, probable toxicity, mutagenesis, and constraints on size and cargo capacity, these vectors do have drawbacks. Alternatives include virosomes and virus-like particles (VLPs). While virosomes are liposome-like particles with integrated surface glycoproteins but lack capsid proteins, VLPs mimic the capsid architecture or envelope proteins of genuine viruses. Both can enclose a variety of payloads, yet neither contains viral genetic material. They hold potential for drug delivery, imaging, immunotherapy, and theranostics because they retain important virus traits, including cellular entrance, immune evasion, and precise targeting [294]. Viral proteins can be applied to NPs to give them additional functionality. For instance, magnetic NPs were enclosed in a hepatitis B core VLP, which improved cellular uptake and demonstrated potential for magnetic resonance imaging [295]. As an alternative, metallic NPs were joined to a specific adenoviral platform, producing an NP-labelled vector that could infect cells and target tumors. Surface alterations of NPs to resemble virus surfaces are a common component of other virus-derived techniques [296].

### 7.2. Surface Modification to Functionalize NPs

A crucial step in customizing NPs for particular biomedical applications is surface modification. The characteristics of NPs can be significantly altered using this rather simple procedure. For instance, coating NP surfaces with PEG might prevent the mononuclear phagocyte system (MPS) from clearing them away. It is possible to vary the surface electrical charges of NP surfaces by precisely adjusting the attachment of functional groups, which can affect how quickly cells absorb substances. Some strategies even try to mimic viruses to give NPs virus-like characteristics. This section lists the common ligands used in surface modifications and their benefits and drawbacks.

#### 7.2.1. PEGylation

PEGylation, which involves bonding polyethylene glycol (PEG) molecules to NPs, was initially published in 1977 and is a widely used method to extend the period that NPs circulate in the bloodstream [297]. PEGylation creates an “anti-fouling” surface for the NPs by forming a hydrophilic brush coating. This layer inhibits aggregation, opsonization, and phagocytosis, preventing the NPs from being quickly eliminated from the body by the mononuclear phagocyte system (MPS). PEGylation has been crucial in developing therapeutic NP uses, from cellular pathways to sonodynamic therapy and tumor targeting for cancer. Additionally, PEGylated NPs have improved stability and biocompatibility in intricate biological contexts. mRNA-based COVID-19 vaccines have benefited greatly from PEG-decorated NPs [298].

PEGylation does have certain downsides, though. Unwanted immunological events, such as hypersensitivity reactions and the production of anti-PEG antibodies, have been documented, especially following the administration of numerous doses of PEGylated NPs [299]. Uncertainty surrounds the precise mechanisms causing these reactions. Despite these drawbacks, PEGylation’s advantages—such as decreased immunogenicity, antigenicity, and toxicity—ensure its relevance in nanomedicine research.

#### 7.2.2. Zwitterions

Researchers are looking toward substitutes like biodegradable poly(glutamic acid), non-biodegradable poly(glycerol), and zwitterionic compounds due to worries regarding PEG’s drawbacks. These zwitterionic materials, which permit charge neutrality and super hydrophilicity by having equal amounts of cationic and anionic moieties, are of great interest. Similar to PEG, they can prolong the half-life of NPs in blood circulation without provoking an immunological reaction. Due to the robust hydration layer that zwitterionic materials create through electrostatic contact, they also resist nonspecific protein adsorption. This characteristic may, however, disrupt target cell communication and lessen the effectiveness of cellular absorption. This difficulty could be overcome by adding a variety of unique functional groups, enabling improved NP stability and biocompatibility, and offering a configurable interface for varied purposes [300]. Zwitterions have been used in some cutting-edge applications, including the development of ratiometric pH sensors based on quantum dots, the creation of reduction-responsive materials to boost intercellular drug-release rates in tumor cells, and the development of pH-sensitive materials to envelop NPs for effective tumor targeting. Additionally, zwitterionic materials can be functionalized to respond to additional stimuli, including temperature or light. For instance, multifunctional NPs coated with zwitterion have demonstrated great capabilities for imaging-guided cancer therapy [301].

#### 7.2.3. Surface Electrical Charge

The absorption and subsequent behavior of NPs are greatly influenced by their surface charge [302]. Positively charged NPs are naturally drawn to cell membranes because they normally have a negative charge, but negatively charged NPs may be less readily taken up by cells. NPs can draw different proteins to them in a complex biological context, creating a “protein corona” on their surface. The qualities and functioning of the NPs could be altered by this process, possibly leading to them losing their intended role or acquiring undesirable traits. The protein corona may alter the surface charges or physical characteristics of NPs, which may enhance the likelihood of non-specific internalization. Surface functional group adsorption or environmental exposure are two ways to change the surface charge of NPs. As a result, when designing NPs, it should be decided whether to use or prevent protein adsorption.

#### 7.2.4. Virus Mimicking

It has been demonstrated that surface alterations of NPs to imitate virus features can improve therapeutic effectiveness and efficiency of distribution. The surface topology of the virus highly influences viral interactions with host cells in question, such as enveloped viruses. Researchers have encouraged interactions between NPs and target cells, thereby improving delivery efficiency by mimicking these topological features, such as adding smaller silica NPs to larger ones to increase surface roughness [303]. Additionally, scientists are imitating viral design, particularly the viral capsid, which is essential for cellular entry and targeting. Viral capsids can be made with synthetic building pieces that give specialized targeting functions, unlike natural virus vectors like virus-like particles (VLPs) or virosomes. One illustration is a multifunctional viral mimic created from self-assembled amphiphilic dendritic lipopeptides that showed the ability to infect solid tumors and tumor cells like a virus and suppress tumor growth in both in vitro and in vivo experiments [304]. The ability of NPs to deconstruct and distribute their cargos—a benefit for intracellular interactions—can be achieved by integrating stimuli-responsive receptors to connect with specified places through viral capsid mimicry [305]. These virus-mimicking methods eliminate the necessity for viral components such as VLPs and virosomes that might cause infections. This field of study has the potential to aid in creating multifunctional artificial “viruses” that could get beyond the drawbacks of the NP drug delivery systems that are now in use [306].

#### 7.2.5. Surface Modification by Bioactive Molecules

Specific biological functionalities can be bestowed on NP surfaces by adding bioactive chemicals [307]. With these alterations, bioactive compounds can selectively target particular cells or tissues, enabling targeted medication delivery. This guarantees effective delivery of medicinal substances to the intended site. NPs can also be made less hazardous through surface alterations, making them safer for biomedical applications. A further benefit is the improved biocompatibility, which enables NPs to operate in a biological context without inducing an unfavorable immune response. Surface modification can be achieved using a variety of methods. Physical adsorption is one technique in which bioactive chemicals are non-covalently attached to the surface of the NPs [308]. Covalent bonding is an alternative strategy that uses chemical reactions to permanently link the bioactive chemicals to the surface of the NPs. The encapsulation method traps the bioactive chemical inside the NP framework [309]. For these goals, several bioactive compounds are frequently used. Targeting certain cell receptors or enhancing the solubility and stability of NPs are possible with peptides [310] and proteins [311]. Chitosan [312] and hyaluronic acid [313] are natural polysaccharides that can facilitate targeted drug administration and improve biocompatibility. Antibodies can target particular cells or pathogens because of their high specificity, whilst some tiny compounds can increase the solubility of NPs or be used for targeting.

The utilization of bioactive compounds offers a number of benefits. Targeting ligands on the surface of NPs, for instance, can increase receptor-mediated endocytosis and increase the effectiveness of cellular uptake. Because some bioactive compounds can react to certain stimuli, such as pH changes [314], temperature changes [315], or the presence of specific enzymes [316], controlled medication release is possible. Additionally, NPs can reduce off-target and negative effects frequently associated with many therapeutic medicines by concentrating on particular tissues or cells. There are, however, issues to take into account [317]. Under physiological circumstances, the NP and the bioactive chemical bond must stay stable. It can be challenging to scale up from laboratory synthesis to larger-scale production without losing the effectiveness of bioactive molecule attachment. Furthermore, therapeutic NP change may be subject to regulatory review and rigorous testing. Using bioactive compounds to modify NPs holds great promise, particularly for biomedicine. NPs can work more precisely and effectively by utilizing these molecules, making them suitable for a range of tasks from imaging to medicine delivery. Although there are obstacles to overcome, current research in this field promises to produce ground-breaking and revolutionary answers.

### 7.3. Functionalization through Geometric Change

Even without any further surface alterations, the geometry of NPs, particularly their size and shape, significantly impacts their characteristics and interactions with cells. The size of the NPs taken up by cells directly affects how cytotoxic they are. Additionally, how NPs interact with cells is influenced by their form. For example, rod-shaped NPs interact with cells more effectively than spherical NPs because they have more accessible binding sites [318]. Designing more efficient NPs requires an understanding of the connection between the geometry of NPs and their functionality.

#### 7.3.1. Size

The potential for endocytosis, a process that enables NPs to be internalized by cells, and the interactions of NPs with cell membranes are strongly influenced by their size. Increasing the number of ligands on NP surfaces to facilitate endocytosis is advantageous, but doing so requires bigger NP sizes. The size must fall within a specific range, though, as NPs less than 30 nm might not be able to drive the membrane-wrapping process as well as those larger than 60 nm. Regardless of the NP core and surface charge, in vitro studies indicate that the ideal range for cell uptake is between 10 and 60 nm [319]. There has been some variation in the effect of NP size on cellular internalization among studies. According to some studies, cellular internalization of functionalized Au NPs is inversely correlated with size [320], whereas, according to others, internalization of Au NPs larger than 50 nm is more common [321]. These differences could result from variances in the gold NPs’ production processes or decorating ligands.

#### 7.3.2. Shape

Beyond only size, NPs’ form greatly impacts how well they interact with cells and deliver drugs. Spheres, rods, triangles, stars, and wires are examples of frequently produced NP shapes [322]. Due to their greater aspect ratio (AR), or length-to-width ratio, compared to spherical NPs, rod-shaped NPs have demonstrated increased effectiveness in cellular uptake [323]. This discovery has sparked additional in vivo research. For instance, after intravenous administration, NPs with bigger ARs were seen to collect in the spleen, while those with lower ARs were probably stuck in the liver of mice [324]. In a different study, mice given rod-shaped MSNs orally showed higher content in all organs than mice given spherical MSNs. This may be explained by the prolonged half-life of rod-shaped MSNs in blood circulation and their capacity to resist macrophage engulfment in RES (reticuloendothelial system) organs, including the liver and spleen [325]. The form of NPs also influences the cellular uptake mechanism. A comparison of gold NPs with star, rod, and triangle forms revealed a substantial relationship between the endocytosis pathway and NP shape, which calls for more research [326]. The decreased internalization rate seen for rod-shaped NPs relative to spherical NPs must be considered, even though larger AR NPs have been connected to higher cellular absorption efficiency [327]. Therefore, the benefits of modifying NP shape must be carefully assessed in the context of particular biomedical applications.

### 7.4. Challenges to Achieve Successful Designed Interfaces

Manufacturing challenges persist, and there is a lack of uniformity in the methods used to fuse cell membrane vesicles with NP cores. Extrusion is a technique that can create uniform particles, but it has complexity and manufacturing scale difficulties. Since cell membrane coating integrity affects internalization, current approaches frequently produce a mixture of fully, partially, and uncoated NPs. The classification of the therapeutic effects of various MCNPs will require further research to determine how to differentiate fully coated NPs. Future MCNP manufacturing efforts should improve procedures and create a successful, all-encompassing process for cell membrane extraction and fusing with NP cores. Moving toward industrial-level output requires automation. In the future, process development should take precedence over discovery more often.

NPs can be functionalized in a variety of ways employing surface modification techniques, sometimes with the addition of ligands or molecules for decoration. Despite its advantages, there are a few difficulties. Due to their effect on cellular absorption efficiency, the density and orientation of ligands on the NP surface must be considered. The complete evaluation of NPs with various surface changes also lacks defined approaches. The geometric features of NPs must also be considered because they can affect cellular absorption and possibly prevent planned functionalization. The next part will go into more detail regarding how NPs’ geometric characteristics affect them.

## 8. Biomedical Applications of Bio-inspired Nanomaterials in Micro/Nanodevices

**Figure 15:**
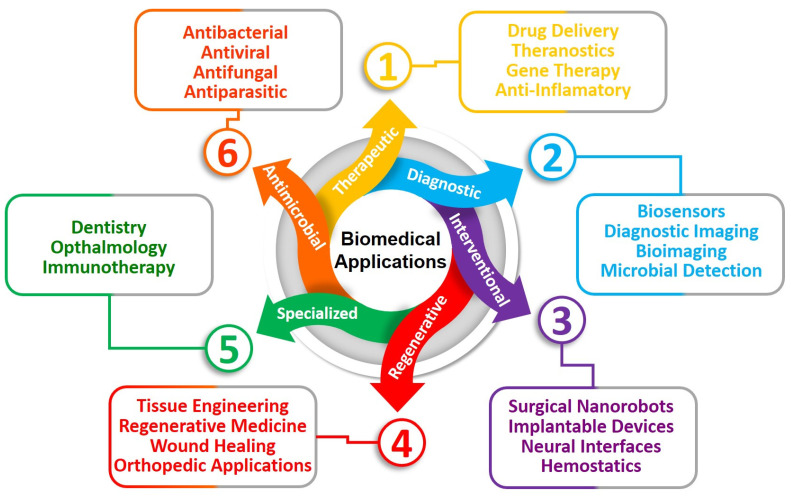
Biomedical applications of BINMs in micro/nanodevices.

When the many uses of bio-inspired nanomaterials in micro/nanodevices are examined, a wide range of biological uses is found. From improving drug delivery methods to coming up with new ways to treat patients, these new materials are paving the way for big changes in healthcare. Drug delivery involves complicated systems, nanotheranostics for combined therapy and diagnostics, gene therapy for genetic disorders, and the creativity of self-propelled active nanovehicles and biohybrid micro/nanomotors that can move through complex biological environments. Bio-inspired nanobiosensors that can identify molecules in a complex way add to these achievements. Also, bio-inspired organ-on-a-chip technology gives us new ways to test drugs, and cancer-on-a-chip models change how we study cancer. Bio-inspired wound healing dressing mats and antimicrobial surfaces, such as those made from structure-oriented peptides, metal/metal oxide NPs, and chitosan, show how bio-inspired nanomaterials have a wide range of uses in medicinal applications. Bacteriophage-based antimicrobial surfaces use the power of nature to fight bacterial diseases. This all-around look at bio-inspired nanomaterials shows their importance and opens up a new era of opportunities for biomedical progress in micro/nanodevices. Figure 15 summarizes the biomedical applications of BINMs in micro/nanodevices.

### 8.1. Drug Delivery and Therapeutic Applications

#### 8.1.1. Drug Delivery Systems

In the field of medicine, BINMs have made major advancements, particularly in the creation of sophisticated drug delivery systems. These nanomaterials can potentially address major difficulties in targeted medication delivery because they were created and constructed to resemble biological structures and processes. Micro- and nanodevices made from BINMs are at the core of these breakthroughs. In several ways, these devices can improve drug delivery. They can increase the specificity of drug delivery, ensuring that the medications reach the cells or tissues that require them most. This can reduce the risk of negative effects while significantly increasing the drug’s effectiveness. Liposomes, for instance, nano-sized vesicles modeled after biological membranes, are frequently utilized as drug delivery systems. They can encapsulate both hydrophilic and hydrophobic medications, guard against deterioration, and release them gradually at the intended spot. Dendrimers, another category of BINMs, are spherical, highly-branching NPs. They are good candidates for targeted drug administration due to their well-defined structure, controllable size, and surface functional groups. Recently, scientists have started investigating the use of BINMs for targeted drug delivery that imitates the structure of cells, bacteria, and viruses. For instance, NPs with red blood cell (RBC) membrane coatings have shown promise in drug delivery while dodging the body’s immunological response. But creating these biologically inspired micro- and nanodevices is difficult. Addressing concerns with stability, biocompatibility, scalability, and reproducibility is necessary. Extensive testing and clinical trials are also necessary to determine the safety and effectiveness of these technologies for human usage.

**Figure 16:**
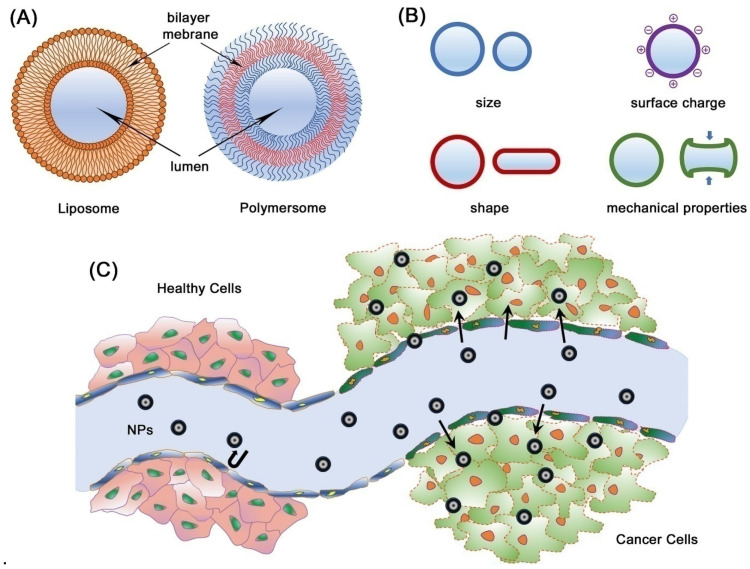
Graphical representations of several polymersome and liposome-related topics. (**A**) the structural differences between polymersomes and liposomes are shown in cross-section. (**B**) The main physicochemical characteristics of nanocarriers are depicted. (**C**) The enhanced permeability and retention (EPR) effect. It is a passive buildup of nanocarriers through fenestrated endothelial cells in tumor tissues. Due to their leaky vasculature and compromised lymphatic outflow, tumor tissues are more conducive to nanocarrier accumulation. Reprinted with permission from Ref. [329], Copyright 2023, Elsevier.

Liposomes are widely recognized as a promising and versatile means of drug delivery. Liposomes present several advantages in comparison to traditional drug delivery systems. These advantages include targeted delivery to specific sites, controlled and sustained release of drugs, protection against degradation and clearance, enhanced therapeutic outcomes, and reduced toxic side effects. These beneficial characteristics have contributed to the effective authorization and medical utilization of numerous liposomal pharmaceutical products within recent decades [328]. Liposomes can be divided into several categories depending on their lamellarity and compartment structure. These categories include multivesicular liposomes (MVLs), unilamellar vesicles (ULVs), oligolamellar vesicles (OLVs), and multilamellar vesicles (MLVs). OLVs and MLVs both have an onion-like structure; however, MLVs have more than five lipid bilayers, while OLVs only have two to five concentric lipid bilayers. On the other hand, MVLs have several non-concentric aqueous chambers that are each surrounded by a single bilayer lipid membrane, giving them the appearance of a honeycomb. Small unilamellar vesicles (SUVs, 30–100 nm), large unilamellar vesicles (LUVs, >100 nm), and giant unilamellar vesicles (GUVs, >1000 nm) are subcategories of ULVs based on particle size. According to several research studies, ULVs come in various sizes, including SUVs that are less than 200 nm and LUVs that are between 200 and 500 nm in size. Numerous techniques are applied for the preparation of liposomes. The manufacturing methods that are frequently utilized encompass thin-film hydration, ethanol injection, and double-emulsion techniques. The conventional procedures in these processes encompass several steps. Firstly, multilamellar vesicles (MLVs) or unilamellar vesicles (ULVs) are prepared, depending on the chosen method. Secondly, the size of the vesicles may be reduced if deemed necessary. Thirdly, the drug solution(s) are prepared and loaded into the liposomes. In the case of passive drug loading, this step is combined with step one. Fourthly, buffer exchange and concentration are performed if required. Fifthly, sterile filtration or aseptic processing is carried out. Lastly, if deemed necessary, lyophilization is conducted, followed by packaging. Figure 16 shows graphic representations of several polymersome- and liposome-related topics. In Panel A, the structural differences between polymersomes and liposomes are shown in the cross-section. The main physicochemical characteristics of nanocarriers are depicted in Panel B. The enhanced permeability and retention (EPR) effect, shown in Panel C, is a passive buildup of nanocarriers through fenestrated endothelial cells in tumor tissues. Due to their leaky vasculature and compromised lymphatic outflow, tumor tissues are more conducive to nanocarrier accumulation [329]. The liposome-inspired drug delivery system is very promising in multidimensional biomedical applications, including pulmonary nanotherapeutics [330], tumor-targeted therapy [331], anti-biofilm agents [332], and multiple diseases [333].

**Figure 17:**
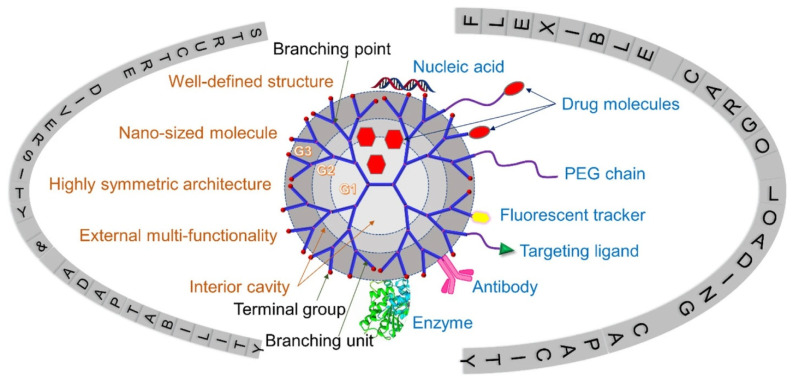
Dendrimers with a wide range of structural configurations and versatile cargo-loading capabilities for Generation 1, Generation 2, and Generation 3, correspondingly. Reprinted with permission from Ref. [334], Copyright 2022, Authors (CC BY 4.0).

**Figure 18:**
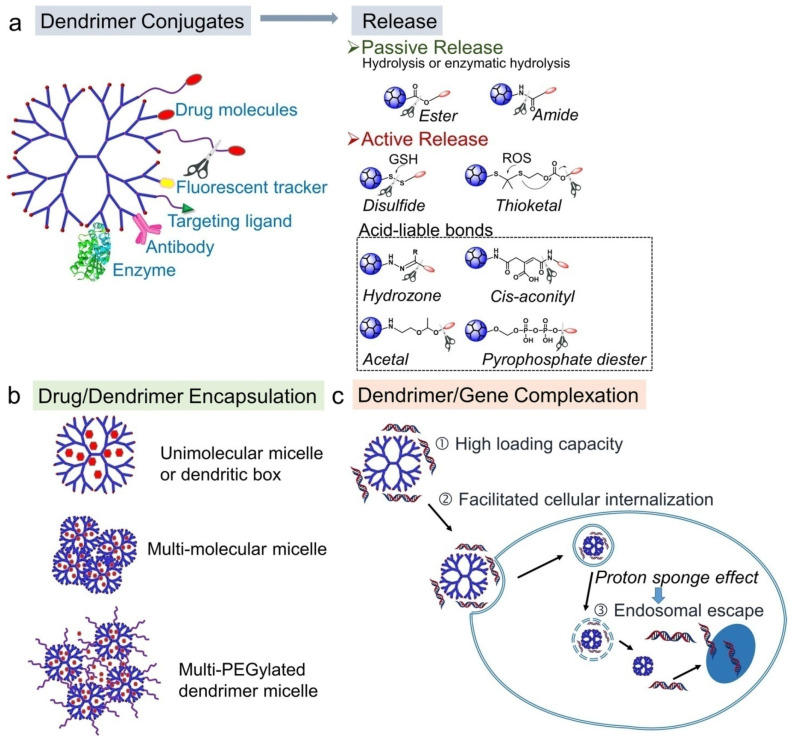
Strategies for dendrimers in drug and gene delivery: (**a**) dendrimer conjugation and cleavable linkers for enhanced delivery, (**b**) exploring three drug/dendrimer encapsulation models, (**c**) dendrimer-mediated gene complexation, and advancing transfection efficiency with cationic dendrimers. Reprinted with permission from Ref. [334], Copyright 2022, Authors (CC BY 4.0).

Dendrimers have emerged as crucial nanostructured carriers in nanomedicine for treating numerous diseases [334]. Thanks to their structural diversity, they can deliver medications and genes in various ways (Figure 17). For example, dendrimers with a hydrophobic center and a hydrophilic periphery can act like unimolecular micelles and successfully saturate hydrophobic medicines. The use of cationic dendrimers as non-viral gene carriers is widespread. Drugs and functional moieties can be attached to dendrimer surface groups to increase stability and solubility. Enhancing dendrimer compatibility and binding properties involves conjugating them with polymers like PEG or polysaccharides. Utilizing ligands like hyaluronic acid or mannose has improved tumor penetration and targeted distribution to specific cell types, such as macrophages. Compared to free medicines, dendrimer–drug conjugates have fewer systemic side effects and more localized efficacy. Dendrimer conjugation can lengthen the half-life of pharmaceuticals, improving medicinal efficacy and reducing administration frequency. Dendrimers increase the solubility of drugs, increasing their potency. When compared to timolol maleate, a study on a dendrimer–drug combination known as DenTimol demonstrated encouraging outcomes for the treatment of glaucoma. Various cleavable or stimuli-responsive linkages ensure that medications released from dendrimer–drug conjugates reach the intended area. For this aim, disulfide/thioketal linkers and pH-responsive linkers are frequently employed. Dendrimer–drug conjugates have promise as efficient drug delivery systems with controlled release mechanisms for better therapeutic results. Figure 18 illustrates the strategies for dendrimers in drug and gene delivery. Over free medicines, dendrimer–drug conjugates have several benefits, such as fewer systemic side effects and increased efficacy at the target site. They can lengthen a drug’s half-life and make it more soluble, enhancing patient compliance and therapeutic results. For instance, PAMAM dendrimers have been utilized successfully to deliver antiglaucoma medications, exhibiting better effects on decreasing intraocular pressure. Examining drug release from such conjugates is crucial since regulatory agencies’ classification of dendrimer–drug conjugates might be complicated. Drug release from dendrimers in tumor cells has been facilitated by cleavable linkers like disulfide and thioketal, increasing the effectiveness of cancer therapy. By serving as “unimolecular micelles” or “dendritic boxes,” dendrimers also provide drug encapsulation through their hydrophobic cavities, enhancing the solubility of hydrophobic medicines in water. PAMAM dendrimers have also been extensively used as gene transfection vectors because of their great biocompatibility and capability for nucleic acid loading. They can improve endosomal escape and cellular uptake, increasing transfection effectiveness. Dendrimers can overcome intracellular gene delivery hurdles when decorated with functional moieties like peptides. This results in successful gene delivery and tumor growth inhibition. Overall, dendrimer-based medication and gene delivery systems provide considerable promise for treating various disorders using nanomedicine [335,336,337].

Polymeric micelles (PMs) are nanostructures created through amphiphilic block copolymers (ABCs) self-assembled in an aqueous medium. These micelles possess a distinctive core-shell architecture. In conventional micelles, the hydrophobic segment of the polymer is oriented toward the interior, forming the core, whereas the hydrophilic segment is positioned on the outer surface. Reverse micelles exhibit an orientation that is opposite to that of regular micelles. Mixed micelles are generated by adding solubilizers to the existing surfactant micelle structure. The drug is expelled into the micelle core by the hydrophobic component of the copolymers, thereby enabling the solubilization of pharmaceuticals with low solubility. The intermolecular hydrophobic interactions between the drug and copolymers are important in modulating the drug release rate and enhancing its solubility. Numerous hydrophobic copolymers have undergone testing to solubilize drugs with low solubility efficiently. Polymeric micelles frequently contain a hydrophobic core enclosed by hydrophilic copolymers [338]. The hydrophilic portion of the polymer faces outward in normal micelles, while the lipophilic portion faces the core. The orientation of reverse micelles is the opposite. Solubilizers are included in the surfactant micelle to create mixed micelles. Pharmaceuticals that are difficult to dissolve are ejected into the micelle core by the copolymer’s hydrophobic component. Copolymers’ hydrophobic interactions with the medication are essential for reducing drug release and increasing solubility. It has been tested that different hydrophobic copolymers can successfully solubilize poorly soluble medicines. Drug leakage from polymeric micelles must be reduced during distribution, and drug release must be regulated to provide optimal therapeutic targeting. Either stable confinement of the drug payload within micellar cores or triggered release in response to internal or external stimuli are necessary for targeted drug delivery. While slowly released medications from capsules allow for pharmacological and toxicological effects, prolonged drug release from polymeric micelles in circulation assures congruent pharmacokinetics with the micelles. Stimuli-sensitive polymeric micelles use internal triggers, such as changes in pH, redox potential, temperature, enzyme profiles, and oxygen levels, to take advantage of disease-induced changes in target tissues. Outside stimuli, including heat, ultrasound, near-infrared light, or magnetic fields can also trigger drug release. These methods improve the adaptive drug carriers’ ability for precise drug delivery, especially in sick tissues like malignant ones. Micelles are distinguished by their core-shell structure. The corona shell shields the drug from the mononuclear phagocyte system, allowing for longer blood circulation and less toxicity. They make it possible for hydrophobic medicines to become stable and water-soluble, facilitating effective medication delivery. The ideal micelles for dispensing hydrophobic medications feature a hydrophilic corona to protect and stabilize the medication. Medicines with a high water solubility can have their intravenous administration of hydrophobic medicines made possible by polymeric micelles. Although polymeric micellar systems have several drawbacks, different methods have been created to overcome these obstacles. With the right approaches to drug loading issues, scale-up options, and thorough research into their behavior in biological systems, polymeric micelles can successfully find their place in the market for various biomedical uses. Polymeric micelles present promising opportunities in biomedical applications [339,340].

#### 8.1.2. Nanotheranostic

**Figure 19:**
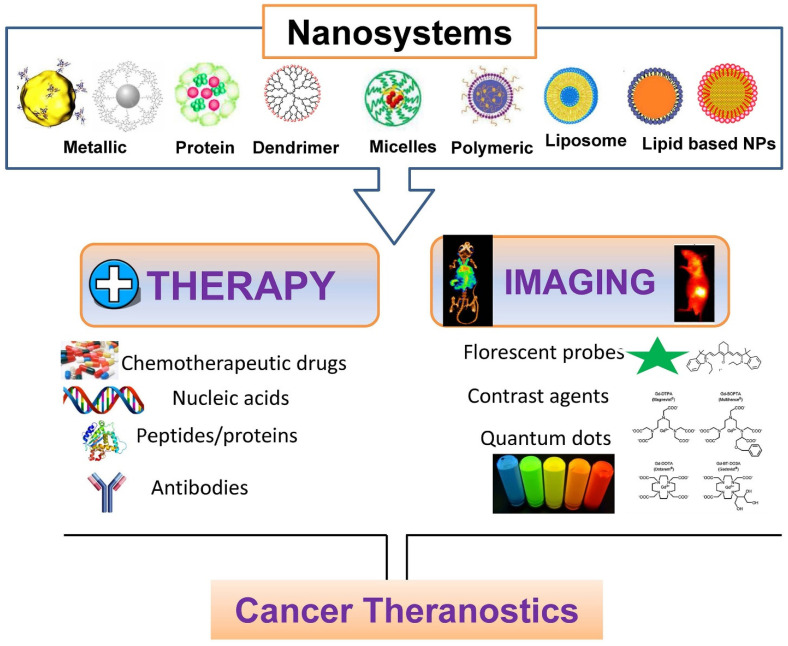
Nanotheranostics: metallic, protein, polymeric, lipid-based, micelle, and dendrimer nanomaterials for cancer theranostics. Reprinted with permission from Ref. [342], Copyright 2019, Authors (CC BY).

For many illnesses, including AIDS, cancer, and microbial disorders, theranostic methods have been suggested. The medication is customized using this personalized treatment approach based on unique molecular profiles or the discovery of biomarkers. Nanotechnology advancements can now combine diagnostic and therapeutic approaches on a single platform. Nanomedicines increase the bioavailability of drugs, shield them from deterioration, and enable precise medication distribution within the body. Compared to conventional medicines, nanostages used in nanotheranostics provide simultaneous illness detection and treatment while improving medication penetration. This new field has the potential to significantly help the pharmaceutical and healthcare sectors by facilitating the creation of molecular sensors, imaging agents, and creative therapeutic agent carriers. Immunoassays and colorimetric tests, as well as gene therapy and targeted drug delivery, are nanotheranostic diagnostic and therapeutic tools that have the potential to transform the diagnosis and treatment of a wide range of illnesses, including cancer, AIDS, cardiovascular disease, infections, and burn wounds [341]. BINMs have significantly enhanced cancer diagnostics and treatments, largely due to their small size, ease of modification, high drug-loading capacity (thanks to their large surface-to-volume ratio), and efficient penetration and retention within targeted tissues. Furthermore, their superior biocompatibility, biodegradability, and multifaceted applications in bioimaging, bio-sensing, diagnostics, and therapeutics have escalated their potential in numerous biomedical fields [342]. Due to their potential to serve as alternative, biocompatible drug delivery systems in cancer theranostics, bio-inspired NPs that mimic natural body components have recently attracted a lot of attention. Unlike non-native drug delivery technologies, these NPs have the innate potential to change systemic bio-distribution, which is their main advantage. This review thoroughly explains numerous BINMs used in cancer theranostics, including liposomes, lipid NPs, bio-synthesized metal NPs, virus NPs, protein NPs, and others (Figure 19).

#### 8.1.3. Gene Therapy

In several medical specialties, gene therapies are becoming more and more cutting-edge treatments. Gene therapy first proposed some 45 years ago as a viable treatment for hereditary monogenic illnesses, is currently being used to treat acquired conditions like cancer immunotherapy. The idea was that a single treatment could offer substantial, possibly curative advantages. For instance, gene-based therapies given to cells with a long lifespan may permit the continued production of crucial proteins. Hematopoietic stem cells (HSCs) that have undergone genetic engineering could provide long-lasting cell replacement, eliminating the requirement for ongoing enzyme administration or transfusion therapy. With initial clinical trials in the early 1990s producing poor findings, including little clinical benefit, unforeseen toxicities, and, in some cases, patient fatalities, converting gene therapy concepts into patient care has had difficulties. This caused a shift in attention to the fundamental science underlying gene therapy strategies. With a better understanding of viral vectors and target cells, a new wave of clinical trials in the late 1990s and early 2000s showed promise, but development was hampered by severe toxicities associated with high gene transfer efficiency. The discipline of gene therapy has made enormous strides over the past ten years, with improvements in safety, gene transfer effectiveness, and delivery spurring major clinical advancements. The FDA has approved several gene therapies, and other agencies worldwide have labeled others as “breakthrough therapies.” The science of gene therapy is about to undergo another revolutionary change because of recent advancements in targeted genome editing [343].

The utilization of BINMs has played a crucial role in advancing the field of micro/nanodevices for gene therapy. These nanomaterials are derived from biological systems and can imitate the structures and functions of biological molecules. As a result, they exhibit enhanced biocompatibility and functionality. Nanomaterials, including lipid-based NPs, protein-based NPs, and DNA/RNA-based nanostructures, function as carriers for gene delivery, offering improved stability, specificity, and efficiency. One illustration of this concept involves the utilization of lipid-based NPs to encapsulate nucleic acids, thereby enabling their efficient transport into cells.

Additionally, DNA nanostructures can be purposefully engineered to serve as carriers for therapeutic genes, allowing for direct delivery. Significantly, BINMs possess the capability to undergo modification or functionalization to augment their targetability and mitigate potential toxicity. By capitalizing on these benefits, micro/nanodevices employing BINMs have demonstrated gene therapy potential. Gene therapy aims to address diseases at the fundamental genetic level by mending, activating, or eliminating specific genes. The ongoing investigation and advancement in this particular domain are anticipated to result in the emergence of gene therapy approaches that are both more efficient and secure.

**Figure 20:**
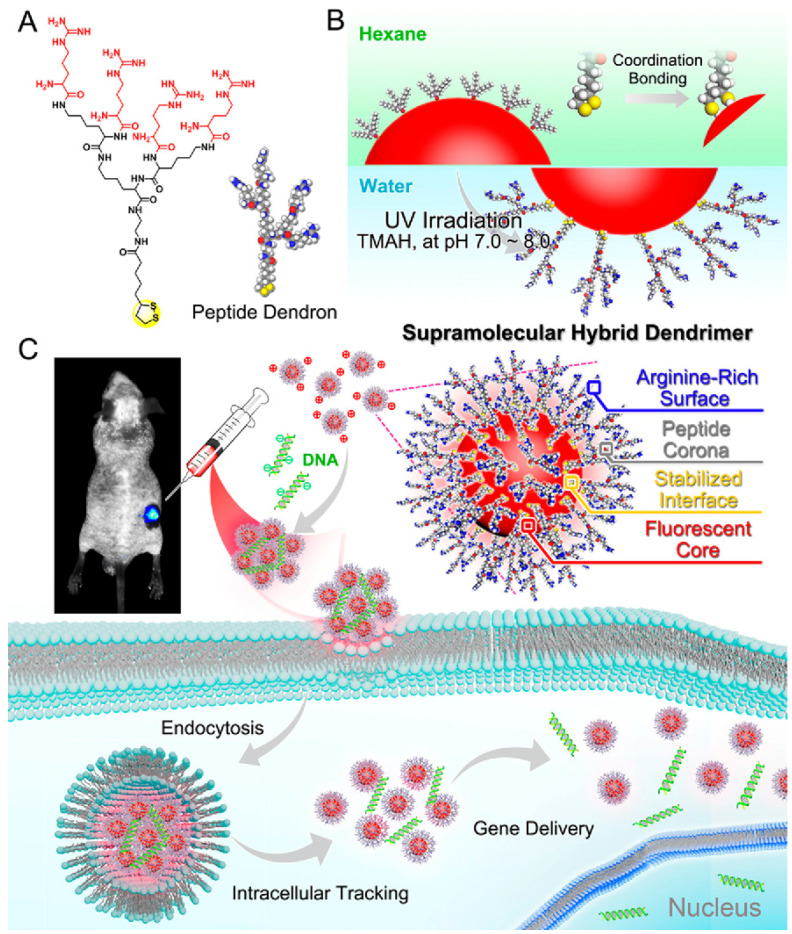
Diagrammatic representations of the self-assembly process and biomedical uses of SHDs. (**A**) Illustration of the chemically dual-functionalized PDs, (**B**) the process of PDs self-assembling onto quantum dots through coordination interactions, and (**C**) use of SHDs featuring complex nanostructures for delivering genes and for biological tracking both in vitro and in vivo. Reprinted with permission from Ref. [344], Copyright 2014, American Chemical Society.

There is a growing interest in the supramolecular self-assembly of dendrons and dendrimers as a powerful and challenging method for generating advanced nanostructures that exhibit exceptional properties. Xu et al. proposed a novel approach involving supramolecular hybridization to fabricate a dendritic system inspired by biological systems [344]. This system demonstrated remarkable versatility and can be utilized as an efficient nanoplatform for various delivery applications (Figure 20). Multifunctional supramolecular hybrid dendrimers (SHDs) were formed by integrating dual-functionalized low-generation peptide dendrons (PDs) onto inorganic NPs, facilitated by an intelligent design. The structural composition of these superhydrophobic surfaces (SHDs) exhibited a highly organized nanoarchitecture, accompanied by a substantial presence of arginine peptides, and demonstrated the ability to emit fluorescence signals. As predicted, the utilization of a bio-inspired supramolecular hybrid strategy dramatically enhances the gene transfection efficacy of self-assembled hydrogel NPs (SHDs) by approximately 50,000 times when compared to standalone polymeric NPs (PDs) at equivalent ratios of polymer to DNA. The bio-inspired self-assembled hydrogel NPs (SHDs) demonstrate several advantageous characteristics in gene delivery. Firstly, they possess low cytotoxicity and are resistant to serum, which enhances their safety and efficacy. Secondly, these SHDs have inherent fluorescence, monitoring various intracellular processes, including cellular uptake, escape from endosomes, and gene release. Lastly, they can serve as a valuable reference for tracking the expression of desired proteins, providing an alternative method for assessing gene delivery efficiency. Significantly, it is important to note that in vivo animal trials have shown that self-healing hydrogels (SHDs) exhibit considerable effectiveness in gene transfection, specifically in muscle tissue and HepG2 tumor xenografts. These trials have also demonstrated the ability of SHDs to perform real-time bioimaging. The anticipated outcome of these supramolecular hybrid dendritic (SHD) structures is the stimulation of research inquiries to utilize bio-inspired dendritic systems for biomedical purposes, encompassing laboratory-based and live organism studies.

With the progress of molecular biology, pharmacogenomics, and proteomics, there is an opportunity to customize the development of bio-inspired nanosystems to cater to individual patients’ specific requirements. Bio-inspired nanomaterials provide numerous advantages, such as the ability to customize their surface, achieve targeted delivery, possess specific geometric properties, ensure biosafety, and facilitate proper disposition. Furthermore, the materials and procedures employed in fabricating these systems exhibit biocompatibility and environmental sustainability, as they necessitate limited processing compared to synthetic materials. Nevertheless, it is essential to acknowledge the potential apprehensions regarding residual solvents or reagents utilized during the synthesis process, which may harm biological systems. However, certain bio-inspired nanosystems exhibit enhanced pharmacological efficacy. There is expected to be a preference for gene carriers based on smart materials that are biocompatible, biodegradable, and safe in the future. These systems can detect the surrounding environment of the host following administration, enabling them to regulate the release of gene molecules that have been loaded within them at a particular target organ with accuracy and pre-determined control. This functionality serves to reduce the occurrence of undesired side effects. Therefore, utilizing bio-inspired gene delivery systems presents a distinct opportunity to develop anticipatory and individualized delivery systems for currently available medications, thereby holding great potential in shaping the future of the biomedical field [345,346].

#### 8.1.4. Self-Propelling Active Nanovehicles

Chemical navigation is a crucial aspect of survival for a wide range of organisms, from bacteria to unicellular and multicellular organisms. The replication of these behaviors through artificial constructs is an emerging field of study, resulting in the development of active nanomaterials capable of converting external energy into mechanical work to achieve directed motion [347]. These nanomaterials can react to various stimuli, including chemical gradients, temperature changes, magnetic fields, and adhesion forces. Nevertheless, the development of self-propelling nano-constructs encounters various obstacles arising from physical limitations. For instance, water, which exhibits a high viscosity at the NP level, poses a significant challenge. Additionally, the randomizing effect of Brownian thermally driven fluctuations further complicates the control of the NPs’ directionality. Two strategies can be employed to address these limitations. The first strategy involves inducing non-reciprocal movements by altering the body shape. The second strategy consists in taking advantage of gradients that modify the local environment of the nanomaterials. Illustrations of these strategies being implemented encompass the utilization of artificial bacterial flagella, which can be effectively manipulated by applying rotating magnetic fields to generate propulsion. The deployment of “spermbots” has been observed, wherein these microscale robots can facilitate the transportation of sperm cells toward the oocyte. Certain NPs can generate gradients autonomously, resulting in self-phoresis or self-propelled movement. Various innovative applications have been documented, including the utilization of silicon nanowires that exhibit a responsive behavior to externally manipulated electrical fields, as well as the development of “microbullets” capable of vaporizing biocompatible fuel and effectively penetrating and altering the structure of tissues. The utilization of bio-inspired methodologies in the design of NPs exhibits considerable promise for their application in drug delivery, targeted therapy, and various other domains within the biomedical field [348,349,350].

#### 8.1.5. Biohybrid Micro/Nanomotors

Throughout history, human ingenuity has frequently drawn inspiration from diverse natural biological systems, exemplified by the development of radar technology, which was influenced by bats’ utilization of ultrasonic waves. The advancement of autonomous artificial micro/nanomotors has been motivated by the existence of biological biomotors such as kinesins, dyneins, and sperm cells. Micro/nanomotors are devices capable of converting various types of energy into mechanical motion, enabling them to execute tasks that passive devices cannot accomplish [351]. Richard Feynman initially introduced the notion of these diminutive devices which has subsequently emerged as a prominent subject of scholarly investigation. Micro/nanomotors possess a diverse array of applications, particularly within biomedicine. These applications encompass drug delivery, biosensors, biological imaging, assisted fertilization, and microsurgery. Nevertheless, notable obstacles must be surmounted, with particular emphasis on the biocompatibility of said motors. To operate optimally, these entities must adapt effectively to the internal microenvironment of the organism, encompassing factors such as temperature, pH, and the immune system. Presently, the utilization of artificial motors is constrained by their inadequate biocompatibility, resulting in their susceptibility to immune system recognition upon introduction into the human body. To enhance biocompatibility, scholars are currently investigating the utilization of biocompatible and biodegradable substances such as polyethylene glycol (PEG) and magnesium. An emerging area of study pertains to biohybrid micro/nanomotors, wherein synthetic materials are integrated with biological constituents. Biohybrid motors exhibit enhanced biocompatibility, improved energy conversion efficiency, and the capacity to react to environmental stimuli intelligently. The cell is the building block of an organism and has receptors on its membrane that enable it to sense environmental inputs and modify its functions. Some cells have complex time-irreversible strokes and low-Reynolds-number autonomous motion processes. Researchers have used this autonomous mobility as motivation to build micro/nanomotors based on intact cells. High biocompatibility, adaptability to diverse internal conditions, and the ability to mix artificial micro/nanostructures with different cell properties like chemotaxis, magnetotaxis, and anaerobism to create multifunctional motors are all benefits of these biohybrid motors. A cell must be simple to grow and capable of large-scale, rapid multiplication to be a good candidate for biohybrid micro/nanomotors. There are several types of micro/nanomotors based on intact cells (sperm cells, bacteria, algae, blood cells, plant pollen, platelets, macrophages, etc.) and different biological components such as enzymes (catalase, urease, glucose oxidase, lipase, etc.) and cellular membrane (RBC, platelet, WBC, tumor cell, cancer cell, etc.) coating, operating in biomedical applications.

Sperm cells, also known as spermatozoa, are the male gametes that possess the ability to exhibit autonomous motility as a result of their flagellar structure. They possess the dual functionality of functioning as both a propulsion mechanism and a means of transporting goods, enabling autonomous movement and precise distribution capabilities. The micro-bio-robot design involved using sperm cells confined within a microtube, which demonstrated self-directed movement that was externally regulated through the implementation of a magnetic layer. Sperm cells possess considerable potential as vehicles for drug delivery, particularly in gynecologic ailments such as cervical cancer [352]. A micromotor propelled by sperm cells has been developed to facilitate the targeted release of drugs, demonstrating encouraging attributes for cancer treatment. Bacteria are abundant and come in various shapes, making them suitable candidates for biohybrid micro/nanomotors. Several bacteria, such as *Magnetococcusmarinus*, *Escherichia coli*, and others, have been utilized to fabricate biohybrid motors for biomedical applications. Magnetotactic bacteria can achieve self-propulsion using external magnetic fields, making them attractive for drug delivery and tumor targeting. Escherichia coli are frequently used due to their swimming ability, and they have been incorporated into micromotors for drug delivery and anti-tumor efficacy. Challenges include addressing safety concerns regarding pathogenic bacteria and ensuring the activity and fitness of bacteria on certain surfaces. Despite these challenges, bacterial biohybrid micro/nanomotors hold promise for advancing the field of micro/nanomotors.

Algae exhibit remarkable biological features despite lacking roots, stalks, and leaves. Spirulinaplatensis (Sp) is a suitable bio-template for biohybrid magnetic micromotors due to its naturally intact three-dimensional helical structure. Researchers used Sp to construct porous hollow micromotors to deliver medicinal and imaging chemicals in vivo. Sp-based biohybrid magnetic robots feature intrinsic fluorescence, MR signals, and low cytotoxicity, making them intriguing for blocking abnormal cell function, particularly malignant tumors, while retaining normal cell function. Sp-based magnet-powered microswimmers use ultrasonics to stimulate neural stem-like cell development. Ultrasound intensity can influence brain stem cell development, enabling minimally invasive neurodegenerative disease treatments. Algae, particularly Sp, have distinctive structures and intriguing biological features, making them promising in modern biotechnology. For biohybrid micro/nanomotors, different algae must be studied.

### 8.2. Bio-Inspired Nanobiosensors

Sensors are pivotal in many products, systems, and manufacturing processes, offering valuable feedback, monitoring capabilities, safety enhancements, and other advantageous features. When conventional sensor technology reaches a state of limited progress, exploring insights from non-engineering disciplines, such as biology, can foster innovative advancements. The field of biomimetic sensor technology is currently in its nascent stage, and it takes inspiration from the intricate sensory systems found in nature. These highly refined systems enable organisms to perform tasks such as navigation, spatial orientation, and prey detection with excellent efficiency. Engineers can construct various types of sensors by comprehending the fundamental principles of sensory physiology in biological systems. Biomimetic sensor designs can replicate biological systems directly or employ analogous principles. Both methodologies have demonstrated efficacy and yielded notable progress in sensor technology [353]. Biomimetic sensor designs offer distinct advantages in comparison to conventional sensor designs.

Retrieving archived sensor design information is a prevalent methodology in developing novel products that detect commonly encountered parameters. Nevertheless, employing unconventional approaches or drawing inspiration from diverse fields of study may be imperative in the context of atypical parameters. The field of sensor design has been influenced by nature, as it presents a wide range of sophisticated sensing and communication techniques observed in diverse organisms such as bacteria, plants, insects, mammals, and reptiles. The design of biomimetic sensors entails replicating various aspects of biological systems, including functional design, morphological design, principles, strategies, behaviors, and manufacturing techniques. The motivation behind these biomimetic devices is derived from rigorous methodologies, careful examination of natural phenomena, and the application of databases that document biological functionalities. Emulating the functionality, principles, morphology, or strategies observed in biological systems represents a form of biomimicry, which can be likened to the reverse-engineering process. In an alternative perspective, abstracting biological systems through analogical reasoning can be seen as an approach that aligns biology with engineering design principles. This approach involves seeking solutions to biological challenges by drawing inspiration from and imitating existing designs. Exploring natural phenomena to derive design inspiration or gain insights into the mechanisms by which biological systems process sensory information has resulted in notable advancements. The biological sensors found in nature have evolved over an extensive time spanning billions of years. These sensors provide enduring and efficient solutions that are well adapted to specific ecological niches. Frequently, these sensors demonstrate characteristics such as minimal energy consumption, heightened sensitivity, and redundancy. The redundancy concept serves as a valuable lesson derived from nature, as numerous biological systems exhibit multiple instances of redundancy to augment reliability and mitigate errors.

Chirality, also known as mirror dissymmetry, is an inherent characteristic observed in geometric entities, and it holds significant importance in biomolecules such as proteins and DNA. Circular dichroism spectroscopy is a technique used to evaluate the impact of a substance on biological, chemical, and physical characteristics. This method quantifies the disparity in the absorption of left and right circularly polarized light. Chirality is regarded as a principle inspired by biology in engineering. Chiral nanomaterials exhibit potential for various applications, such as sensing and catalysis, owing to their distinctive selectivity and specificity.

Nevertheless, there remains a lack of comprehensive understanding regarding the mechanisms that govern the transfer of chirality during the synthesis of inorganic nanomaterials possessing inherent chirality. Examining biological instances of chirality transfer can provide valuable insights for developing chiral inorganic nanomaterials across diverse applications. Chirality is a prevalent characteristic observed in biological entities, significantly influencing their geometries, properties, and behavior. Chiral objects, characterized by the absence of mirror symmetry, are widely observed in the natural world, and they have the potential to confer survival benefits to organisms. The phenomenon of chirality significantly influences the preferential incorporation of amino acids during the process of protein synthesis, thereby exerting a profound impact on the growth and behavior of both plants and animals. Organisms can utilize chiral structures to perceive polarized light and augment contrast within their surroundings. Inorganic materials also observe chirality due to molecular interactions and biological templates. Gaining insight into the processes by which chirality is transferred across various length scales is of utmost importance to effectively replicate and harness chiral nanostructures to design nanomaterials. The phenomenon of hierarchical chirality transfer, which occurs across multiple scales ranging from the molecular to the macroscopic level, has been documented in diverse biological systems. This observation has sparked interest and served as a source of inspiration for developing biomimetic materials and nanotechnologies [354]. Near-infrared (NIR) wavelengths are commonly favored in biomedical applications owing to their superior tissue penetration capabilities. The utilization of CdTe helices has been observed to effectively manipulate light within the near-infrared (NIR) wavelengths, rendering them valuable for various biomedicine and optical computing applications. Chiral molybdenum oxide NPs have the potential to be utilized in photothermal therapy, wherein they can selectively heat tumor tissue when exposed to circularly polarized light while minimizing damage to healthy tissue [355]. The bactericidal effects of gold nanobipyramids conjugated with D-Glu are enhanced, disrupting bacterial cell walls and facilitating the healing process in infected wounds when exposed to near-infrared (NIR) radiation [356]. The inherent structural chirality exhibited by gold nanomaterials can influence the immune system. Diverse immune responses are observed with left-handed and right-handed Au NPs, owing to their distinct interactions with specific receptors and subsequent activation of inflammasomes. The utilization of left-handed NPs as adjuvants in the influenza vaccine has been investigated, revealing a notable increase in antibody production and immune-related cell proliferation compared to right-handed NPs. These discoveries underscore the significance of nanoscale chirality within biological systems, alongside the molecular-scale chirality exhibited by L/D optical centers.

### 8.3. Bio-Inspired Organ-on-Chip (OOC)

Creating new medications is time-consuming and expensive, especially in the pre-clinical stage. Pre-clinical research has a history of using unethical animal experiments that do not always precisely anticipate how people will react to medications. Although they provide an alternative, two-dimensional cell culture models cannot match the intricacy of real tissues and organs. Three-dimensional cell culture models have been created to overcome these restrictions; however, they still lack some physiological elements. The development of organ-on-chip (OOC) systems, which are little devices that replicate the microenvironment of organs and tissues, has recently been made possible by microtechnology. OOCs can build human-based tissue-like structures, operate with minuscule drug concentrations for high-throughput screening, and add biosensors for real-time monitoring of cell survival and functionality, among other benefits. To replicate the response of different tissues to drug exposure methodically, several OOCs can be coupled. OOCs are a potential strategy for drug discovery because they combine the benefits of 2D and 3D cell culture models while offering a platform for drug testing and screening that is more physiologically appropriate [357].

#### 8.3.1. Organ-on-Chip (OOC) Technology

The primary objective of OOC technology is to develop in vitro models that closely mimic the physiological conditions of human organs. This is achieved by integrating cell cultures within microfluidic channels and structures. These systems provide the benefits of a microenvironment that closely resembles physiological conditions and utilize human cell lines that have been extensively studied and characterized. Out-of-cell culture systems possess the inherent capability of parallelization and enhanced throughput, rendering them highly advantageous in drug screening. Nevertheless, constructing organic optoelectronic devices necessitates utilizing advanced manufacturing techniques and selecting meticulously chosen materials. Cell cultures are immobilized on substrates and structures in OOC devices. These chip systems’ cells can be divided into three major categories: primary, immortalized, and stem cells. Primary cells are taken straight from tissues or organs without being altered. They closely mirror their in vivo counterparts’ appearance and metabolism. Primary cells must be obtained, kept alive, and only cultivated temporarily.

Additionally, standardization might be challenging due to differences in cell populations and traits between extractions. Immortalized cells are standardized, easily accessible, and well-characterized cells. They can be made from clinical malignancies or by modifying original cells chemically or virologically so that continuous cell division lasts for a long time. However, relative to their initial in vivo state, the cells’ phenotype may change during immortalization. Because of their physiological properties and regulated differentiation potential, stem cells hold great promise. Induced pluripotent stem cells, produced by reprogramming adult tissues to produce pluripotent stem cells, are becoming increasingly popular due to ethical considerations and the restricted availability of embryonic stem cells. These cells can be differentiated into multiple cell types, facilitating research like personalized drug testing and autologous tissue engineering. In 2012, induced pluripotent stem cells’ discovery was given the Nobel Prize.

#### 8.3.2. Organ Systems on Chips

Due to demographic shifts and the rising demand for new pharmaceuticals in pharmaceutical research, society is exposed to an increasing number of novel chemicals in today’s modern, globalized world. Reliable testing methods are required to ensure these substances are safe and effective. Animals were used mostly in the early toxicological, pharmacological, and environmental testing stages. The 3R approach (reduction, refinement, and replacement of animal experiments) has prompted a move toward alternative techniques. The development of alternative techniques has been encouraged by regulatory bodies like the European Parliament and the Council of the European Union, which has led to the EU’s prohibition on cosmetics containing chemicals that have undergone animal testing. According to industrial firms, alternative approaches offer the potential to advance fundamental research, medicine development, toxicity testing, and environmental studies. Excellent throughput screening, parallelization, excellent data quality, predictability in clinical trials, and cost savings are among the alternatives they are looking for to eliminate the usage of animals. Common in vitro systems, however, cannot fully satisfy all of these demands, which has increased demand for enhanced in vitro models and cutting-edge OOC technologies.

#### 8.3.3. Two-Dimensional Cell Culture to OOC

Early in vitro cell culture models were two-dimensional (2D), but it soon became clear how important three dimensions were. Cell morphology and metabolism were improved by 3D cell culture employing extracellular matrix (ECM) components [358]. Predictability was improved by creating cell–cell interfaces by integrating various cell types onto the semiconductor. Microsystems, inspired by developments in the semiconductor industry, permitted controlled trials with smaller drug doses. They made it possible to imitate in vivo circumstances by precisely controlling the biological milieu and inducing physiological pressures or gradients. Surface alterations made possible by microtechnology also encourage cell self-organization. These developments boosted predictability and complexity without raising variability. For widespread usage in pre-clinical studies, OOC systems must accomplish simplicity, dependability, reproducibility, and ease of use.

#### 8.3.4. Single OOC (SOOC)

Researchers have pursued the development of integrated body systems on a chip through a systematic approach, wherein the initial focus has been on creating individual organ chips that can subsequently be interconnected. The development of these SOOCs was facilitated through the utilization of microchip technology and the progress made in the semiconductor industry. The lung-on-chip was among the initial organ chips documented in Science magazine in 2010, garnering considerable interest [359]. Subsequently, many biomimetic organ systems on chips have been successfully developed. There are several SOOC systems, including liver-on-chip [360], kidney-on-chip [361,362], lung-on-chip [362], gut-on-chip [363], heart-on-chip [363], muscle-on-chip [364], blood–brain barrier-on-chip [365], splenon-on-chip [366], bone marrow-on-chip [367], etc.

#### 8.3.5. Multi-OOC (MOOC)

The utilization of MOOCs represents an interim measure in investigating inter-organ interactions until a fully functional human organ system is realized on a chip. The integration of multiple organ chips enables the examination of intercellular communication and the assessment of different stages of drug metabolism. Two primary approaches exist to construct multi-organ chips: the linkage of pre-existing single-organ chips and the integration of multiple organs into a singular chip device. The latter methodology has been proposed by the TechnischeUniversität Berlin and TissUse GmbH, commencing with a biotechnological device that integrates two distinct compartments, namely the liver and skin [368]. The utilization of a chip system, comparable in size to a conventional microscope slide, facilitated enhanced spatial efficiency and ensured appropriate ratios of physiological fluid to tissue. Researchers have made progress in the step-by-step integration of supplementary organs, such as the small intestine, liver, renal secretion, and skin biopsies, thereby advancing the development of a comprehensive human-on-chip system [369,370].

#### 8.3.6. Human-on-Chip (HOC)

The primary objective of OOC technology is to develop a human-on-chip (HOC) model that replicates the functionalities of several vital organs within a singular microfluidic platform. Numerous governmental initiatives, including those sponsored by the American Defense Advanced Research Projects Agency (DARPA) and the National Institutes of Health (NIH), provide financial backing for research endeavors in this particular domain. There exist two primary approaches in designing an HOC system: the first involves the interconnection of individual single-organ chips, while the second entails the integration of distinct organ compartments onto a single chip. It is imperative to surmount the obstacles encountered in engineering and implementation to attain precise emulation of physiological conditions and dependable predictions of drug effects [371]. One of the primary challenges in this context involves selecting appropriate cell types and mediums while also considering immune responses and the inherent variability in blood composition. Streamlining the culture conditions and chip construction is advisable to enhance the results’ clarity. Additionally, implementing a modular plug-and-play system could facilitate the interconnection of compatible chips in subsequent endeavors [372]. The active participation of the pharmaceutical industry and regulatory agencies is imperative to achieve successful development and validation of organ systems on chips.

#### 8.3.7. Patient-on-Chip (POC)

Stem cells are a type of cellular entity characterized by their undifferentiated state and their capacity to differentiate into diverse specialized cell lineages. There are two primary classifications of stem cells: embryonic stem cells, which are obtained from embryos, and adult stem cells, which are sourced from adult tissues. The utilization of embryonic stem cells in research is hindered by ethical considerations, thus leading to the prevalent use of human-induced pluripotent stem cells (HiPSCs) as a viable substitute. HiPSCs are derived through reprogramming mature cells, acquiring characteristics akin to embryonic stem cells. This reprogramming enables HiPSCs to undergo differentiation into diverse cell lineages upon exposure to specific molecular cues. HiPSCs present a multitude of benefits in the realm of scientific investigation and medical interventions. One potential application of these technologies is the generation of patient-specific tissues for tissue engineering purposes and facilitating patient-specific drug testing. These advancements have the potential to enhance the field of personalized medicine. Incorporation of patient-derived HiPSCs into OOC systems presents a promising approach that synergistically harnesses the advantages of microfluidic technology and genetically compatible human cells. This methodology enables the replication of pathological conditions and genetic variations, enhancing the applicability and precision of drug testing and research endeavors. In addition, utilizing HiPSC technology holds promise in facilitating the advancement of a comprehensive HOC platform. Utilizing HiPSCs derived from a single donor to generate diverse organ tissues can potentially mitigate the occurrence of immune reactions. Furthermore, examining patient-specific variables, including genetic factors, age, gender, and ethnicity, can be readily conducted, thereby facilitating the development of more individualized therapeutic strategies in subsequent endeavors [373]. In general, HiPSCs exhibit considerable potential in facilitating the progression of scientific inquiry and pharmaceutical innovation, culminating in enhanced medical interventions that are more efficacious and tailored to individual patients [374,375,376].

#### 8.3.8. Applications of OOC

Using OOCs exhibits significant potential as in vitro testing platforms for diverse applications. They possess utility in toxicity assessment for cosmetics and chemicals, rendering them indispensable in novel and generic drug advancement alongside specialized domains such as radiation examination. A substantial transformation in pre-clinical test practices toward enhanced efficiency and accuracy can only be achieved through collaborative endeavors. Once successfully designed and validated, OOC systems will substantially impact pharmaceutical research and development. These methods can lessen the need for animal testing and produce more trustworthy outcomes for biowaiver studies for generic formulations and medication development. This enhancement will result in more accurate clinical study forecasts and fewer late-stage failures. Additionally, OOC systems will create new possibilities for pharmacological R&D. They will make it possible to simulate sick creatures, giving researchers a controlled environment to investigate the causes of disease and potential cures. Additionally, custom chips can be produced to customize drug testing for specific patients, resulting in more efficient and individualized therapy. Organ systems on chips can potentially advance pharmaceutical research and significantly transform pre-clinical testing procedures.

OOC presents exciting possibilities for modeling diseases and developing new medications. Researchers have created disease chips to simulate specific disease states and analyze treatment reactions in a controlled setting. A lung-on-chip system was developed by Huh et al. to simulate pulmonary edema, a potentially fatal condition brought on by inflammation and fluid buildup in the lungs [377]. The chip enabled the testing of possible therapeutic substances, including angiopoietin-1 and GSK2193874, and faithfully replicated the effects of interleukin-2 (IL-2) therapy. Nesmith et al. created a bronchial smooth muscle tissue chip to research allergic asthma [378]. The IL-13 and acetylcholine exposure successfully caused the chip to mimic the hypercontraction observed in asthmatic patients. The RhoA inhibitor HA-1077 was put to the test by the researchers, and it showed promise as a possible therapeutic candidate for the treatment of allergic asthma. Tumor spheroids, hydrogels, and ECM proteins have all been used to create in vitro cancer models. Microfluidic systems are being investigated to model tumor formation, tumor–tissue interactions, and metastasis to increase physiological relevance. Researchers can lessen their reliance on animal models by using OOCs to test prospective medications and properly analyze cancer causes. These disease-specific OOCs have demonstrated encouraging outcomes when simulating illness states and assessing medication responses. They have the potential to transform pre-clinical testing, lessen the need for animal testing, and enhance therapy approaches for a range of disorders.

### 8.4. Cancer-on-Chip (COC)

**Figure 21:**
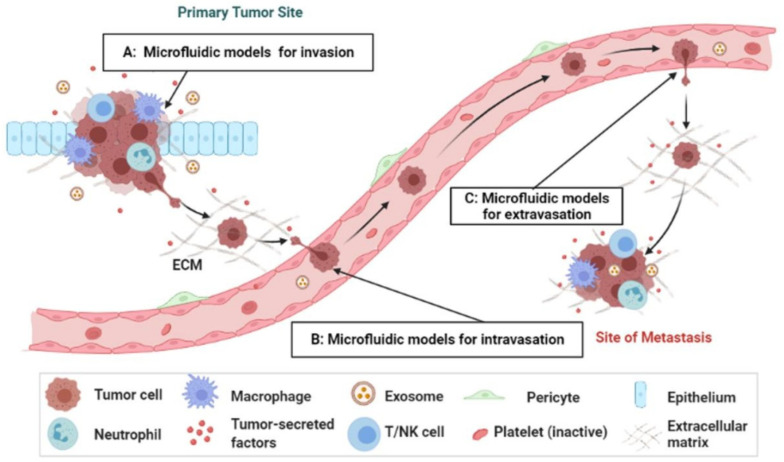
Microfluidic models for tnvasion, intravasation, and extravasation. Reprint with permission of [379], Copyright 2022, Authors (CC BY 4.0).

**Figure 22:**
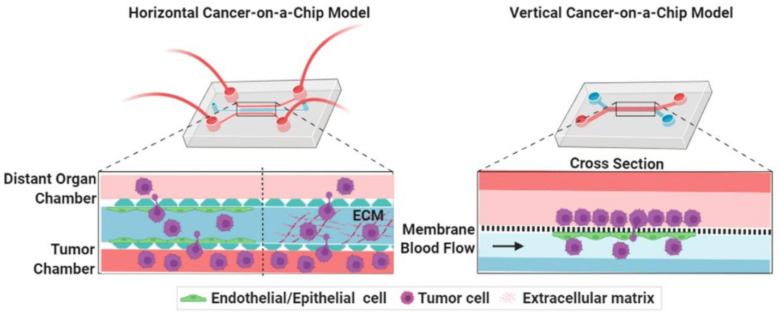
Simple COC models using microfluidics show how cancer cells can spread from their initial site to distant tissues. These designs can be broadly divided into chips that are horizontal and vertical. Different cell types can grow in their zones while still being able to interact with one another thanks to diverse signaling processes in horizontal models where chambers are divided by pillars that are only a few microns in diameter. Channels in vertical chips that simulate both cancer intravasation and extravasation processes are divided by membranes. To replicate more sophisticated tumor behavior, some models may incorporate vertical and horizontal layers, as with the ovarian TME OOC model. Reprinted with permission from Ref. [379], Copyright 2022, Authors (CC BY 4.0).

The utilization of specialized multichannel systems in cancer-on-chip (COC) models has emerged as a potent approach for studying the tumor microenvironment (TME) and its involvement in metastasis. The utilization of microfluidic channels enables the replication of tumors’ biochemistry, geometry, and fluidic transport characteristics by these models, thereby facilitating the examination of intricate interactions associated with metastasis [379]. The functional COC platforms exhibit superior accuracy and capabilities to traditional models, enabling them to provide significant insights into the TME and cell interactions during metastasis. Invasion, intravasation, extravasation, and angiogenesis are all components of the intricate process known as metastasis (Figure 21). Micrometastases are formed when tumor cells extravasate to colonize other organs after invading the extracellular matrix or vascular endothelium and entering circulation. The endothelial blood vessel wall must be broken during the key phases of intravasation and extravasation. For early diagnosis, prognosis prediction, and treatment planning, microfluidic technology holds promise for isolating and counting circulating tumor cells (CTCs). Microfluidic-based COC models are crucial to examine the complex interactions between tumor cells and the TME during invasion, intravasation, and re-growth in secondary organs. These models offer insights that conventional approaches cannot. The primary TME, circulatory microenvironment, and secondary TME are three different tumor microenvironments that interact during metastasis. For cancer cells to pass the endothelium and enter the bloodstream, the extracellular matrix (ECM) must be broken down. Surviving circulating tumor cells (CTCs) multiply and create secondary cancers in distant organs by adjusting to the local microenvironment (Figure 22). The TME contains various stromal elements, including fibroblasts, immune cells, vessels, and ECM. Microfluidic models that continuously expose tumor cell development to biological fluids in a biologically appropriate microenvironment are used to research cancer. Cancer invasion, intravasation, extravasation, and the evaluation of anti-cancer medications can all be studied with the aid of these models.

**Figure 23:**
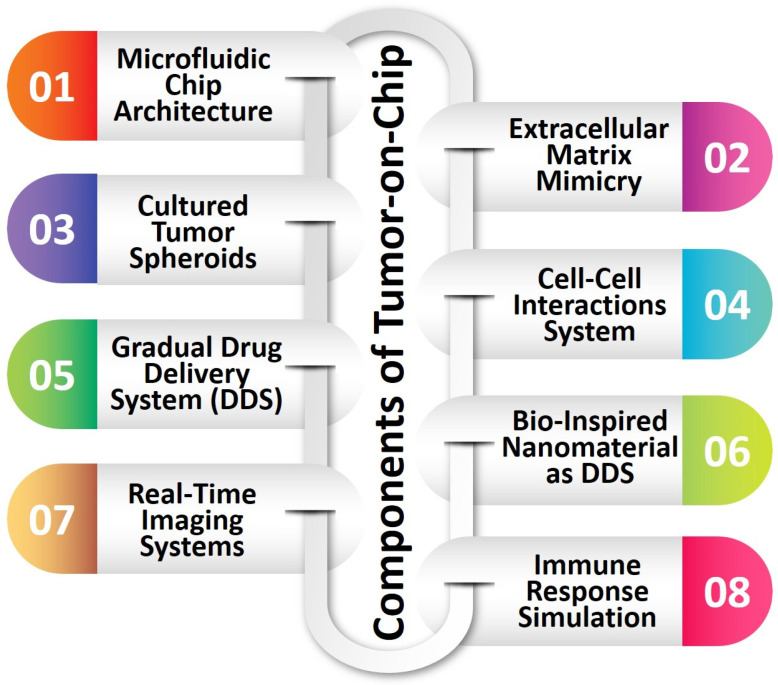
The basic components of TOC.

COC and tumor-on-chip (TOC) are intricately interconnected and serve as mutually reinforcing methodologies within cancer investigation. The primary objective of TOC models is to gain insights into the behavior and characteristics of tumor cells within a precisely regulated microenvironment. These models frequently employ microfluidic channels and compartments to replicate the biochemical and biophysical stimuli that impact the development and advancement of tumors. TOC models have the potential to enhance their fidelity to the TME by including non-tumor cells, such as stromal components, immune cells, and endothelial cells, thereby increasing their complexity. The COC and TOC models employ microfluidic technology to facilitate the uninterrupted provision of essential nutrients, oxygen, and other factors crucial for cellular proliferation and intercellular communication. By establishing physiologically relevant conditions, these models offer more precise depictions of tumor behavior than traditional in vitro cell culture systems. Furthermore, both methodologies can be employed to screen anti-cancer pharmaceuticals and examine the effectiveness of prospective therapeutic interventions. Figure 23 depicts the basic components of a common TOC. The utilization of a tumor-on-chip system fabricated through 3D printing techniques facilitated the cultivation of cells for an extended duration, thereby replicating the intricate process of nutrient and anti-cancer drug transportation within authentic TMEs [380]. The analysis of convective and diffusive transport within the culture chamber was conducted by employing a fluorescent tracer. The GelMA/alginate microbeads were the most efficient in facilitating transport. The microbeads were utilized to cultivate Caco2 cells, and subsequent drug assays mimicking chemotherapy exhibited a rise in cell death and a decline in cellular metabolism. Hypoxic conditions were artificially created within the microspheres, emulating the oxygen-deprived environment typically found in avascular tumors observed in patients. The study showcased the capacity of TOC platforms created through 3D printing for drug testing and examining cancer biology. The increased dimensions of the chip facilitated a higher quantity of biological material that could be used for analysis. The transport characterization demonstrated efficient convective and thoroughly mixed conditions, rendering it a valuable instrument for replicating tumor scenarios and other tissue environments. Additional investigation is warranted to delve into the potential benefits of employing 3D-printed tumor-on-chip systems, specifically regarding their design flexibility and the feasibility of their fabrication and utilization.

### 8.5. Bio-Inspired Wound Healing Dressing Mat

Bio-inspired wound healing dressing mats are a novel class of biomaterials specifically engineered to enhance the process of wound healing by leveraging inspiration from biological systems. These mats are designed to imitate the natural extracellular matrix, creating an environment that promotes cell growth, angiogenesis, and tissue regeneration. These environments provide advantageous conditions for wound healing, diminish the presence of microorganisms, and facilitate the regulated discharge of therapeutic substances such as growth factors and cytokines. Biomaterials derived from natural sources, such as silk proteins (fibroin and sericin), have demonstrated significant promise in wound dressings owing to their biocompatible nature and capacity to stimulate skin tissue regeneration. Integrating regenerative medicine and nanotechnologies offers a potentially effective strategy for tackling the complexities of wound management and promoting improving the healing process.

The integumentary system serves a vital role in numerous physiological processes. However, when the skin becomes compromised due to injuries, it can give rise to significant medical complications, such as heightened morbidity and mortality rates. Non-healing chronic wounds pose a significant challenge, particularly for individuals diagnosed with diabetes, as they may experience limb ulcers that can lead to severe consequences. The principal objective of wound management is to achieve expeditious healing while ensuring both functional and aesthetically satisfactory results. The wound healing process is intricate and encompasses various cellular interactions, secretion of factors, and interactions with the ECM. Comprehending these processes is imperative to formulate efficacious wound management strategies. The impact of diabetes on wound healing is detrimental, highlighting the need for a comprehensive comprehension of the wound environment and pathophysiology to develop more effective strategies for promoting wound healing. Utilizing biomaterials that can release signaling molecules, such as growth factors and cytokines, in a controlled manner has been shown to facilitate the process of angiogenesis and tissue regeneration. The development of effective biomaterials for tissue repair, including three-dimensional living tissues, has been facilitated by advancements in regenerative medicine, nanotechnologies, and bioengineering. Biomaterials derived from biological sources have demonstrated significant potential in treating tissue injuries and enhancing wound healing, owing to their biocompatible nature and capacity to stimulate skin tissue repair [381]. The investigation of biomaterials possessing wound-healing properties has been undertaken for diverse purposes in wound management. These biomaterials create advantageous microenvironments that promote cellular proliferation, inhibit microbial colonization, and facilitate the controlled release of therapeutic agents. The recent progress in wound healing approaches has created novel opportunities within the realm of regenerative medicine and tissue engineering.

**Figure 24:**
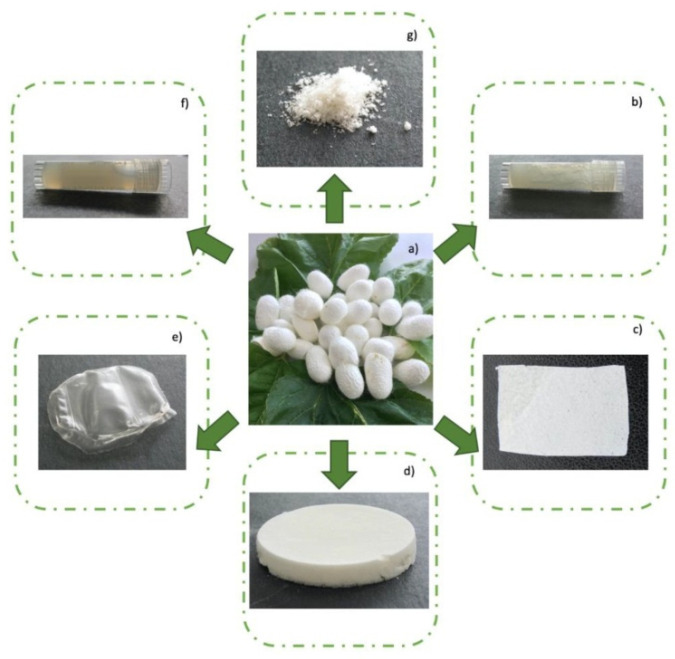
Representation of the instances of BINMs in devices fabricated by processing silkworm cocoons (**a**) for wound healing application: fibroin hydrogel (**b**), electrospun fibroin (**c**), sponge (**d**), film (**e**), solution (**f**), and powder (**g**). Reprinted with permission from Ref. [381].

As a promising biomaterial for tissue repair and regeneration, Bombyxmori’s silk fibroin has attracted much interest [382]. Numerous research teams have investigated the potential of silk fibroin to create cutting-edge methodologies for tissue engineering and wound healing applications, either alone or in combination with other materials and processed in various ways. As biomaterials for wound dressing, many forms of silk fibroin, such as hydrogels, sponges, films, and nanofibers, have been suggested. During various stages of wound healing, these materials maintain moist conditions, permit gas permeability, and improve cell responsiveness. Silkworm cocoon goods include fibroin hydrogel, electrospun fibroin, sponge, film, solution, and powder. These products have a variety of uses in bioengineering, particularly in the treatment of wounds (Figure 24).

### 8.6. Antimicrobial Surface

Using bio-inspired antimicrobial surfaces has generated considerable attention in diverse biomedical contexts owing to their efficacy in combating microbial hazards. These surfaces are influenced by natural defense mechanisms found in plants and microorganisms. They employ light-activated compounds or other biomimetic strategies to generate antimicrobial effects. Within medicine, these surfaces are utilized in various capacities, such as wound dressings, medical implants, and surgical instruments. Their primary function is to mitigate the risk of infections and expedite the healing process. In dentistry, dental implants and orthodontic devices are utilized to minimize bacterial colonization and the formation of biofilms, thereby improving oral health. In addition, implementing bio-inspired antimicrobial coatings on medical equipment and surfaces within hospitals enhances infection control measures and mitigates the potential for healthcare-associated infections. The potential of these bio-inspired antimicrobial surfaces, with their adaptability and inspiration drawn from biological systems, holds significant promise for enhancing healthcare and improving patient outcomes. This is achieved by offering robust protection against microbial pathogens in various biomedical environments.

A range of bio-inspired antimicrobial surfaces have been developed, each exhibiting unique mechanisms of action. Certain surfaces are designed to mimic the micro/nanostructures observed in natural entities such as cicada wings or lotus leaves. These surfaces possess rough and hydrophobic topographies, which effectively hinder the adhesion and colonization of bacteria. Consequently, these surfaces exhibit self-cleaning properties. Some researchers utilize synthetic antimicrobial peptides (AMPs), short chains of amino acids with a wide range of antimicrobial properties. These synthetic AMPs are employed to either disrupt the cell membranes of bacteria or hinder crucial cellular processes. Cationic polymers, which draw inspiration from the positive charge exhibited by antimicrobial peptides (AMPs), interact with bacterial cell membranes with a negative charge. These interactions ultimately disrupt the membrane structure, leading to the demise of the bacterial cells. Moreover, incorporating silver and other metal NPs into surfaces enables the gradual release of antimicrobial ions upon interaction with bacteria. This process disrupts bacterial metabolism and hinders DNA replication. Chitosan-based surfaces, derived from the exoskeletons of crustaceans, offer a biopolymer barrier of natural origin that effectively inhibits bacterial colonization. Mussel-inspired coatings, which utilize polymers functionalized with catechol groups, serve as effective platforms for integrating antimicrobial agents onto diverse surfaces. In addition, photodynamic antimicrobial therapy (PDT) involves the immobilization of light-sensitive agents on various surfaces, which, upon exposure to light, generates reactive oxygen species that effectively eliminate bacteria. In conclusion, using bio-inspired surfaces designed to release bacteriophages, viruses that selectively target and infect bacteria, results in the targeted eradication of bacterial pathogens. Utilizing a wide range of bio-inspired antimicrobial surfaces presents a potential avenue for improving healthcare outcomes and addressing the challenges posed by microbial infections.

#### 8.6.1. Structure-Oriented Surface

Many plants and animals have evolved distinctive surface structures throughout millions of years of evolution, enabling them to endure external threats in difficult environmental circumstances. These organic and synthetic antimicrobial nanostructures, which are crucial to bioengineering, have piqued the curiosity of researchers. These surfaces’ superhydrophobicity and micro/nanotopographies are thought to be responsible for their antibiofouling qualities. Taro leaves, for example, have a unique uneven structural distribution that prevents Gram-negative bacteria from adhering even in humid situations. Staphylococcus aureus has been discovered to resist shark skin’s antibacterial properties. Studies using naturally occurring bactericidal surfaces, such as cicada wings with nanoneedle arrays, have demonstrated the ability to kill bacteria instantly upon direct contact in just 5 min. Dragonfly wings and gecko skin are examples of other species with mechanobactericidal surfaces. Although animal surfaces may have a lower water contact angle than plant surfaces, the antibacterial impact is similar, indicating that hydrophobicity is not the only factor affecting bactericidal effectiveness [383]. Creating next-generation bactericidal surfaces with physico-antimicrobial characteristics has drawn heavily on inspiration from nature. Research into naturally occurring nanostructures has sparked a number of ground-breaking innovations. Researchers fabricate artificial nanostructures on various substrates using bottom-up chemical synthesis and top-down multiway etching techniques. These nanostructures, like carbon nanotubes and ZnO nanorods, have physical and mechanical antibacterial properties that can damage bacterial cell membranes and prevent bacterial adherence. Hydrothermal synthesis and chemical deposition are two surface topography coatings and changes that improve the bactericidal effects. It is possible to replicate nanostructures seen in nature, such as those on cicada wings and pitcher plant surfaces, by combining several processes.

One or both probable manifestations of the antibacterial activity of naturally occurring nanostructured surfaces are the biocidal effect (total destruction of the cellular envelope) or the antibiofouling effect (inhibition of bacterial proliferation). The internalization or insertion of NPs that disrupt membrane function (nanotoxicological effects), physical puncturing, physical tearing, and chemical destructive extraction through oxidative stress are just a few of the factors contributing to the killing mechanism of physico-mechanical antibacterial materials. Mechanical antibacterial materials like carbon nanotubes and graphene impact bacterial adherence, internal cell architecture, and cell migration. These substances repeatedly rupture bacterial cells as part of a cumulative process. According to other studies, bacterial cells are prevented from approaching nanocolumn arrays by the height and spacing of the arrays, leading to a contacting physical puncturing mechanism. When the cells try to migrate on the nanocolumns’ surface, they are broken apart. Some scientists suggest that bacteria produce an extracellular polymeric substance (EPS) under external mechanical stress rather than being directly pierced, which causes bacterial membrane damage through strong attachment to nanocolumns. Since diverse materials and structural configurations may have unique antibacterial effects on different microbes, the precise mechanism is still unclear and up for debate. However, the success of physico-antimicrobial surfaces depends on their specific structures, which are required for their antibacterial capabilities. Compared to chemical mechanisms, the physical antibacterial mechanism is typically faster, and the material’s structure is key to generating an efficient antibacterial effect.

#### 8.6.2. Peptide-Based Surface

Antimicrobial peptides (AMPs) exhibit considerable potential as viable therapeutic options for various diseases, particularly in combating multidrug-resistant bacteria. The global emergence of antibiotic resistance has garnered significant attention, prompting the exploration of alternative solutions, such as AMPs that possess wide-ranging antimicrobial properties. AMPs are synthesized by diverse organisms, identifying more than 5000 distinct AMPs to date [384]. These substances exhibit a specific mode of action by selectively interacting with microbial membranes, resulting in the formation of pores and ultimately leading to the demise of bacteria. Moreover, AMPs exhibit anti-inflammatory, regenerative, and anti-cancer characteristics.

Nevertheless, despite their considerable potential, AMPs encounter certain obstacles in their application. These challenges encompass the toxicity exhibited toward mammalian cells, vulnerability to proteases, and the high costs associated with their production methods. To tackle these concerns, there have been suggestions for using nanotechnology-based delivery methods to augment the stability and biological efficacy of AMPs. The development of bio-inspired NPs has been undertaken to preserve the activity of AMPs while mitigating any potential adverse effects. In addition, AMPs have been employed as surface coatings on implants to mitigate the risk of implant-related infections and promote bone regeneration. AMPs have demonstrated potential in cancer therapy due to their ability to specifically target malignant cells and facilitate the delivery of cancer medications or nucleic acids. AMP-based materials have demonstrated high efficacy in condensing and delivering nucleic acids, thereby protecting against degradation.

#### 8.6.3. Metal/Metal Oxide NP-Based Surface

The wide range of features that metal and metal oxide NPs possess, such as non-toxicity, antibacterial activity, and anti-insecticidal activity, make them useful in the biomedical industry for identifying and treating serious illnesses [385]. Different bio-inspired metals and metal oxide NPs are essential for maintaining life processes, and deficiencies in these substances can cause diseases. For instance, Co NPs exhibit good magnetic, optical, and mechanical properties, making them useful for biomedical applications like magnetic resonance imaging (MRI) and drug delivery, while nanoceria, despite its lack of stability in living systems, shows promising applications in battling cancer and Alzheimer’s disease. Other NPs, such as those made of Au, Ag, Fe, MgO, Ni, Se, and ZnO, also exhibit distinctive properties that can be used in energy storage, biosensing, imaging, and therapies. Chemoresistive nanosensors are created using nanomaterials such as nanorods, nanotubes, and nanobelts, broadening the range of biomedical applications. Overall, diverse features of metal and metal oxide NPs hold considerable promise for increasing biomedical research and healthcare.

#### 8.6.4. Chitosan-Based Surfaces

The antibacterial properties of chitosan have been thoroughly investigated for various uses in the biomedical, cosmetic, food, and agricultural industries. Researchers have studied its usage in self-preserving materials, which have created various goods with antibacterial qualities, including beads, films, fibers, membranes, and hydrogels. Studies on the antimicrobial effects of chitosan have changed over the past 20 years, moving from studies on foodborne and soilborne pathogenic fungi to studies on bacteria, with varied assays and methodologies revealing the underlying mechanisms and factors determining its efficiency. Chitosan’s potential as an antibacterial agent has been further boosted by the development of nanotechnology, which has made it possible to create materials with nanostructures that are more effective at the atomic level [386]. Although the precise mechanism underlying chitosan’s antibacterial activity is not entirely understood, many independent factors have an impact. According to the major hypothesized mechanism, Chitosan adhering to the bacterial cell wall causes cell disruption, changes in membrane permeability, and inhibition of DNA replication, which results in cell death. Chitosan’s polycationic structure, which interacts electrostatically with the anionic components of microbial surfaces, is essential to the substance’s antibacterial effect. Additionally, the shape and size of chitosan particles can affect how they interact with bacterial cell surfaces, with larger NPs behaving differently from smaller ones. Chitosan also has antifungal properties that limit spore germination and radial growth in fungi. Studies have demonstrated its effectiveness against several fungi linked to food and plant rotting. Additionally, chitosan can activate enzymes called chitinases in plant tissues, which act on various fungus species.

#### 8.6.5. Mussel-Inspired Antimicrobial Coatings

Mussel-inspired coatings are developed from the adhesive properties of mussel foot proteins in marine mussels [387]. The proteins in question facilitate the strong attachment of mussels to diverse surfaces within moist and turbulent environments, such as coastal areas in the ocean. Researchers have successfully replicated the bioadhesive chemistry found in mussel foot proteins, resulting in coatings exhibiting strong surface adhesion and antimicrobial characteristics. The fundamental operational principle underlying mussel-inspired coatings centers on integrating catechol molecules. Catechol is a prevalent chemical functional group abundantly present in mussel foot proteins. This collective facilitates robust and enduring adherence to various surfaces, encompassing metals, polymers, ceramics, and even biological tissues. The coatings under consideration utilize catechol groups to establish a durable attachment mechanism by forming covalent and non-covalent bonds with the desired substrate. The antimicrobial properties of these coatings are derived from the distinctive amalgamation of adhesive chemistry and the intrinsic characteristics of catechol. The application of these coatings demonstrates a high level of efficacy in inhibiting bacterial colonization and the formation of biofilms. The presence of adhesive catechol groups results in the disruption of microbial cell membranes, causing destabilization of the membranes and subsequent leakage of vital cellular constituents. Furthermore, the surface roughness and hydrophobicity of the coatings also impede the attachment and proliferation of bacteria. Mussel-inspired antimicrobial coatings significantly advance healthcare outcomes and biomedicine by improving biocompatibility, preventing infections, and supporting tissue regeneration.

#### 8.6.6. Bacteriophage-Based Antimicrobial Surface

Bacteriophages are widespread and are viruses that attack bacterial cells. When Ernest Hankin examined the waters of the Ganges and Jamuna Rivers in India in 1896, he discovered the initial signs of bacterial parasites in the environment. Hankin showed an unidentified material in the river water that has antibacterial capabilities against *Vibrio cholerae*, without specifically identifying phages. While working with Bacillus subtilis two years later, Russian bacteriologist Nikolay Gamaleya noticed a comparable incident [388]. The immobilization of bacteriophages plays a crucial role in advancing biotechnologies, presenting new prospects for detecting pathogenic microorganisms at minimal concentrations, developing materials possessing distinctive antimicrobial characteristics, and facilitating fundamental research on bacteriophages. Bacteriophages of indigenous origin exhibit a notable propensity for a particular bacterial species, and in some cases, a discernible subspecies, primarily due to the recognition of epitopes on the capsid proteins [389]. By means of chemical or genetic modifications, the binding specificity can be modified, thereby enabling redirection toward a diverse range of substrates and analytes beyond the scope of bacteria. Therefore, the attachment of bacteriophages to flat and particulate surfaces is a rapidly growing area of significant scientific fascination [390]. Table 2 thoroughly summarizes all the subsections under this section and lists the most recent biomedical applications of micro/nanodevices fabricated from BINMs.

## 9. Challenges for Bio-Inspired Nanomaterials in Biomedical Applications

**Figure 25:**
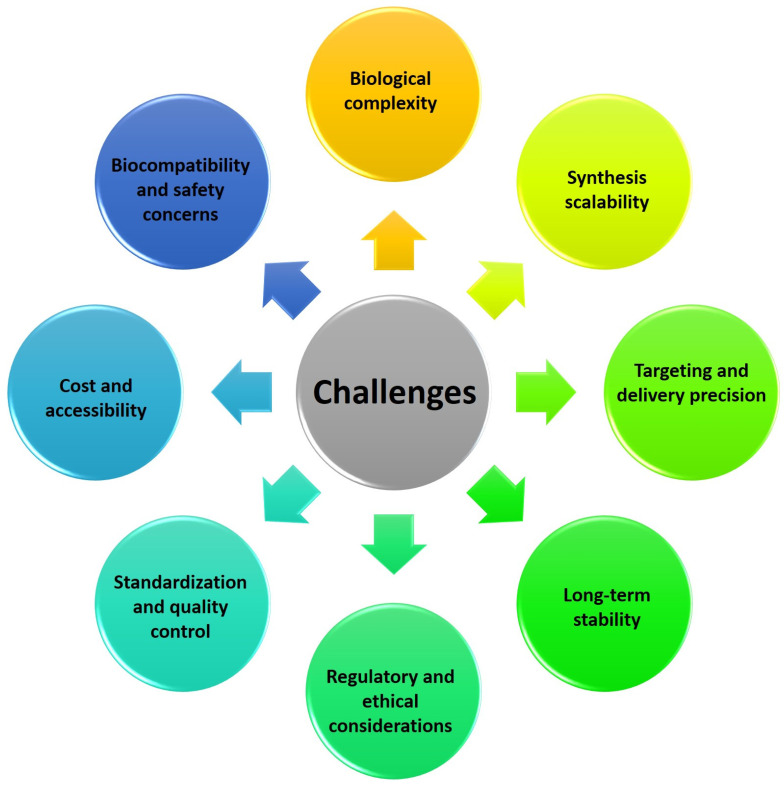
The challenges faced by the BINM-based micro/nanodevices in biomedical applications.

The application of BINMs in biomedicine holds great promise and potential. However, several challenges must be addressed to incorporate these materials and ensure widespread adoption. The biomedical applications of BINMs are still facing some challenges related to biocompatibility and safety concerns, biological complexity, synthesis scalability, targeting and delivery precision, long-term stability, regulatory and ethical considerations, interdisciplinary collaboration, cost and accessibility, standardization, and quality control (Figure 25).

### 9.1. Biocompatibility and Safety

One of the foremost challenges lies in the assurance of biocompatibility and safety of BINMs within intricate biological systems. The cytotoxicity (effects on cell activities and survival) and biocompatibility of BINMs intended for biomedical applications must be assessed [493]. Both in vitro and in vivo tests are included in this biocompatibility assessment. They must pass tests for cytotoxicity, carcinogenicity, reproductive toxicity, immunotoxicity, irritation, sensitization, hemocompatibility, systemic toxicity, and pyrogenicity on BINMs. These assessments are essential for the security of manufacturing employees and patients receiving BINM therapies [494]. Furthermore, a fundamental understanding of the connection between the BINMs’ physicochemical characteristics and their particular biological effects is required to improve their application. For instance, studies show that BINMs can limit cancer formation and manage the scarring process [495]. However, because of the wide variety of BINMs, the abundance of testing model systems, the absence of standardized testing techniques, and the difficulties involved with in vivo tests, difficulties occur when measuring bio-interactions.

#### 9.1.1. Cytotoxicity and Genotoxicity of BINMs

The cytotoxicity of BINMs might be caused via direct necrosis, induced apoptosis, or immunological clearance, depending on their composition, molecular structure, and size. Cytotoxicity studies frequently evaluate metabolic impairment, cell-death marker production, and damaged cell membranes. Several particular tests such as the LDH test (tracks the release of LDH, which is generally present in healthy cells), Caspase-3/7 (measures the amount of caspases produced, which are responsible for apoptosis), and MTT assay (this assay particularly measures the reduction of the tetrazolium salt utilizing redox indicators to gauge metabolic activity changes to evaluate cell viability) are suitable for assessing BINMs. Cell viability may be impacted by BINMs, depending on their composition or geometry [496]. Even though some substances may not result in cell death, they can nonetheless have sub-lethal effects on the genome and epigenome, particularly at lower dosages. The genotoxicity of BINMs has been extensively studied, and common procedures include the Ames test, comet test, micronuclei test, DNA laddering test, and chromosome aberration test. Recently, chemical mutagenicity assessments have also used next-generation sequencing [497]. The ability of BINMs to directly penetrate cells or to catalyze intracellular OH radical generation, which can enhance ROS and DNA damage, is crucial to comprehend [498].

#### 9.1.2. Immunomodulation of BINMs

Both direct and indirect immunomodulation, including immunosuppression and immunostimulation, can be caused by BINMs [499]. Although BINMs can be used to deliver drugs or vaccines utilizing NPs, this article focuses on the immunological reactions that the BINMs cause. Adaptive immunity involves T cells and B cells creating antigen-specific reactions, and innate immunity, which deals with non-specific interactions with immune cells like macrophages, is involved in this. For instance, zinc oxide and silver BINMs have been found to increase the production of IL-6 and IL-8 in kidney cells, indicating improved innate and adaptive responses [500]. Size, surface chemistry, molecule structure, and chemical content of BINMs all impact their immunomodulatory activities. Particularly important in immunomodulation is particle size. For example, NPs (193 nm) induced a stronger immunological response than their microparticle equivalents (1530 nm) [501]. Larger particles may stimulate a stronger serum immunoglobulin response [499]. Identifying sensitivities to specific BINM features is difficult due to the diversity of immunological responses from different BINMs and the varied testing procedures. The intricacy of interpretations can also be increased by the synergistic influence of several components on these processes [502].

#### 9.1.3. Fibrosis Induced by BINMs

BINMs can cause fibrosis, a condition characterized by the excessive accumulation and modification of the extracellular matrix (ECM). Since fibrosis can develop without causing an immediate reaction and because there are no established prognostic tests, our understanding of it is limited. Cells are examined for the expression of fibrosis-related proteins, such as α-smooth muscle actin, transforming growth factor beta (TGF-β), and other ECM components like fibronectin, laminin, and COL I, during in vitro tests for fibrotic reactions. Cells are routinely grown on rigid substrates (in 2D cultures), which might alter gene expression due to the nucleus pore opening from mechanical stresses. This is a known problem. This implies the necessity of in vivo research [503]. Due to their tiny size, NPs can easily enter lung alveoli. They can trigger a variety of reactions, including fibrosis when they come into touch with lung cells. For instance, NPs can increase the production of TGF-β and reactive oxygen species (ROS) [504]. The relationship between NPs and these negative reactions may be complicated because these consequences may not cause immediate discomfort. There are several different ways that NMs cause fibrosis. Unintended immunogenic responses, cytotoxicity, and potential long-term impacts on human health are significant issues that necessitate comprehensive examination and resolution. The nanomaterials produced through biosynthesis exhibited negligible toxicity concerning hematological, biochemical, histological, and DNA damage assessments [505].

### 9.2. Biological Complexity

Nanomaterials must navigate a complicated biological environment with its many cellular networks, interlaced routes, and multiscale mechanisms. These tiny structures meet a continually changing physiological and metabolic milieu when introduced into such systems. Therefore, understanding and forecasting nanomaterial behavior and, more significantly, biological system response is a huge problem. Proteins in the living environment can build a “protein corona” on NPs. Unintended adsorption can drastically change the nanomaterial’s biodistribution, half-life, and therapeutic efficacy. The protein corona can also elicit immunological responses, which may remove nanomaterials quickly or cause unexpected adverse effects. Nanomaterials interact dynamically with cells, life’s building components. Depending on size, charge, and surface properties, nanomaterials may be endocytosed or diffused by cells. On the other hand, cellular absorption can be harmful. It may be useful for delivering treatments directly into cells but may also cause cytotoxicity or interfere with biological functioning. Tissues and cell clumps increase this intricacy. Some tissues are nanomaterial-permeable, while others are impenetrable. The blood–brain barrier blocks most chemicals, including nanomaterials, from entering the brain. Overcoming such constraints without harming the nanomaterial’s functionality demands a delicate balance of design and innovation.

### 9.3. Synthesis Scalability

The synthesis of BINMs frequently encompasses intricate procedures, elaborate molecular architectures, and meticulous functionalization, necessitating a high level of complexity and precision. Scaling up these processes to achieve mass production while preserving their inherent properties and quality poses a substantial technical challenge. A comprehensive approach is employed to address the intricate biological complexities related to using BINMs in biomedical applications. The process entails comprehensive biocompatibility evaluations, including in vitro and in vivo investigations to assess potential cytotoxicity, immunogenicity, and long-term consequences. Furthermore, customized surface modifications and functionalizations are implemented to augment the biocompatibility and targeting specificity of nanomaterials, thereby minimizing any detrimental interactions with biological systems. Again, there has been significant progress in developing sophisticated computational models capable of simulating and predicting the interactions of nanomaterials within intricate biological environments. This advancement has greatly facilitated the process of designing and optimizing the performance of these nanomaterials. Implementing these collaborative approaches plays a significant role in effectively navigating the complex biological environment and facilitating the secure and efficient incorporation of BINMs across various biomedical contexts.

### 9.4. Targeting Delivery

BINMs have expanded biomedical applications, including medication delivery and diagnostics. These NPs can selectively reach desired tissues or cells without disrupting healthy ones, maximizing therapeutic efficacy and minimizing negative effects. Molecular or cellular interaction is a major benefit of nanomaterials in medicine. Their small size lets them negotiate the complex blood vessel network and reach even the most inaccessible body areas. Simply being small is not enough. Surface features, including charge, hydrophobicity, and functional groups determine how these NPs interact with biological substances and cells. Complex systems like the human body have several defenses to identify and destroy alien things. Avoiding the immune system is difficult when using nanomaterials for therapy. Researchers can make these NPs “invisible” to immune cells or use specific biological processes to improve their targeting by altering their surfaces. Active targeting is another trending method. NPs are functionalized with ligands or antibodies that bind to target cell receptors. NPs can bind selectively to cancer cells that overexpress certain receptors, delivering therapeutic chemicals precisely where they’re required without harming healthy cells. The regulated release of medicinal compounds from NPs is crucial. A delayed or inadequate release may not be helpful, whereas an abrupt or excessive release may be harmful. Scientists can sustain and control medication release by modifying nanomaterial composition and structure, keeping drug concentration within the therapeutic window. External stimuli like pH, temperature, or light can also regulate release. Certain nanomaterials release their therapeutic payload in reaction to tumor cells’ acidic environment or inflamed tissues’ high temperatures.

### 9.5. Stability of BINMs

The long-term stability of BINMs within the biological environment is paramount in ensuring sustained therapeutic efficacy. Managing factors such as degradation, aggregation, or alteration of properties over time is necessary. Several techniques can be used to increase the BINMs’ long-term stability for biomedical applications. These include meticulously choosing biocompatible and stable materials, surface modification to prevent degradation, controlled release systems to control therapeutic agent release, encapsulation within protective matrices, use of crosslinking agents to increase stability, thorough biocompatibility testing, in vivo research for in-the-moment insights, and computational modeling to forecast behavior. Researchers can address stability issues and guarantee the long-term effectiveness of BINMs in challenging biological settings by employing these techniques.

### 9.6. Regulatory and Ethical Considerations

The field of nanomaterials in biological applications is characterized by both technological advancements and complex regulatory and ethical considerations. As scientific advancements approach the limits of feasibility, it becomes increasingly imperative to exercise prudence, guaranteeing the responsible introduction of innovations, taking into account considerations of personal well-being and broader societal ramifications. From a regulatory standpoint, the journey from a laboratory notion to a commercially viable product is intricate and complex. Regulatory agencies, such as the Food and Drug Administration (FDA) or the European Medicines Agency (EMA), require a thorough compilation of supporting documentation prior to granting permission. The data presented encompass more than simply the clinical effectiveness of the treatment and explore the possible long-term adverse effects, environmental consequences, and wider societal implications associated with introducing a novel therapeutic approach. This frequently entails conducting extended clinical trials, comprehensive toxicity assessments, and implementing rigorous production protocols to guarantee uniformity and excellence.

The ethical considerations are similarly, if not more, complex. As the manipulation of materials at the nanoscale and their subsequent introduction into the human body are undertaken, inquiries emerge: Has full informed consent been acquired from the patients? Do individuals possess an understanding of the potential long-term ramifications, particularly in cases when these ramifications remain uncertain? How can we effectively promote equitable access to these potentially transformative therapies? Does the potential exist for the exacerbation of pre-existing inequality in healthcare? The issue of privacy arises, especially when these nanomaterials interact with digital technologies. Which individuals or entities are granted access to the data, and what measures are being implemented to ensure their protection?

The environment constitutes an additional dimension inside the ethical framework. The environmental implications of the production, utilization, and disposal of NPs warrant investigation. Do these entities undergo decomposition or persist, resulting in unanticipated ecological disturbances? The multifaceted nature of BINMs also gives rise to many prospects and complexities. Integrating knowledge from other fields can contribute to developing comprehensive solutions, underscoring the importance of proficient interdisciplinary communication. Every academic field is characterized by its own unique vocabulary, methodology, and priorities. It is of utmost importance to prioritize establishing effective communication and a shared vision to prevent the fragmentation of efforts, which may result in overlooking crucial aspects. Fundamentally, the field of bio-inspired nanomaterials for biomedical purposes exhibits significant potential. However, this pursuit is accompanied by many factors surpassing mere scientific obstacles. Each progression is a nuanced choreography of originality and accountability, necessitating attentiveness, anticipation, and a dedication to the collective welfare.

### 9.7. Cost and Accessibility

BINMs are expensive because they require high-end facilities, specialized researchers, and rare raw ingredients. Between basic research and commercial production are many stages, each with its own costs. Fundamental research might take years, followed by synthesis refining, safety studies, and rigorous clinical trials before medicinal use. These trials are necessary to ensure nanomaterial safety and efficacy but are time- and resource-intensive. Other economic aspects must be considered besides production costs. Medical material safety and efficacy regulations can be lengthy and costly. Many nanomaterials are unique, and therefore, regulatory authorities may require extra testing, increasing expenses. Accessibility is another issue, especially for global healthcare. Developed nations may have the facilities and resources to invest in these modern therapies, whereas emerging or undeveloped places may not have the funds. Even if these components are obtained, a lack of skilled staff or facilities to conduct treatments may render the technology worthless. The issue goes beyond procurement. Specialized equipment or conditions may be needed to store and preserve these fragile nanomaterials, straining limited resources. Distributing these items worldwide, especially to rural areas, might be a logistical nightmare without compromising their efficacy. While nanomaterials are expensive, their potential benefits are great. Their capacity to target specific cells or tissues, limit side effects, and even offer new treatments puts them at the forefront of modern medicine. This makes cost and accessibility issues even more important. The effectiveness and accessibility of BINMs must transcend geographical and economic boundaries to improve healthcare. Researchers, industry leaders, and politicians must work together to achieve this balance.

### 9.8. Standardization and Quality Control

Establishing standardization protocols and implementing quality control measures play a crucial role in guaranteeing uniform quality, reproducibility, and adherence to standardized testing methodologies for BINMs. These factors are of utmost importance in facilitating the effective translation of such materials into clinical applications. Overcoming these challenges necessitates the integration of scientific advancements, meticulous experimentation, adherence to regulatory standards, and cooperative endeavors involving researchers, medical professionals, policymakers, and industry stakeholders. The successful resolution of these obstacles will facilitate the efficient implementation of BINMs in various biomedical domains, encompassing diagnostics, drug administration, tissue engineering, and regenerative medicine.

### 9.9. Recent Developments and Commercial Viability of BINM-based Micro/Nanodevices

#### 9.9.1. Commercially Available Nanobiosensors

When a biosensor demonstrates superior performance during real-sample testing, it can proceed to commercial production. The analytical capability of the sensor in practical scenarios will determine its market feasibility [506]. Several biosensors have achieved considerable commercial success, tracking metrics such as blood glucose, cholesterol, malaria, HIV, and uric acid [507]. Furthermore, sensors monitoring cancer and cardiac diseases have garnered significant commercial interest [508]. The affordability of production and scalability are essential for these devices to thrive in the market. To make them more economical, innovations like paper-based and chip-based microfluidic technologies have been introduced [509]. These tools ensure accurate sample management and precise analyte detection. Due to the low cost of paper, paper-based biosensors are attractive for both manufacturers and consumers. A variety of clinical biosensors such as a blood profiler from abbott (iSTAT) (https://www.pointofcare.abbott, accessed on 15 August 2023), glucose monitoring system from allmedicus (GlucoDr) (https://www.lelong.com, accessed on 15 August 2023), blood hemoglobin analyzer (AimStrip) (https://www.1cascade.com, accessed on 15 August 2023), uric acid detector from ApexBio (UASure) (https://redmed.pl, accessed on 15 August 2023), integrated printed circuit biosensor from Acreo (https://www.acreo.se, accessed on 15 August 2023), and pregnancy dipstick from alere (hCG combo) (https://www.alere.com, accessed on 15 August 2023) have been adopted commercially that offer both excellent analytical results and speedy detections. There is also a surge in emerging technologies, including cost-effective prototypes built on paper, elastomers, and combinations of the two [510]. Traditional diagnostics, which can be slow and require bulky equipment, are inadequate for urgent or remote medical situations. In contrast, advanced biosensors address these challenges with swift, on-the-spot testing capabilities. Yet, the high diagnostic needs often outpace traditional methods and current commercial biosensing devices in areas hit hard by epidemics or pandemics. Therefore, there is an immediate demand for scalable, cost-effective prototypes to handle future disease outbreaks better.

#### 9.9.2. Commercially Available Drug Delivery System

Drug delivery systems (DDSs) composed of tiny molecules, peptides, nucleic acids, proteins, and cells commonly encounter obstacles in the delivery process, impeding commercial product development progress. The investigation has uncovered three primary solutions for addressing delivery challenges, which encompass the modification of the medication itself, manipulating the drug’s surrounding environment, and developing a delivery system that can effectively regulate drug interactions within its microenvironment [511]. Small-molecule-based DDSs face challenges related to biodistribution, half-life, exposure, concentration, solubility, permeability, target development, and off-target toxicity. Commercial manufacturers addressed these issues by developing an osmotically controlled release oral-delivery system for methylphenidate HCl (Concerta) to address the drug tolerance issue by regulating its pharmacokinetics [512]. Ritonavir, commercially known as Norvir, is a protease inhibitor often used to treat HIV. It has been chemically modified with thiazole to enhance its metabolic stability and solubility in aqueous environments [513]. Benazepril, commercially known as Lotensin, is an alkyl ester prodrug that is designed to conceal ionizable groups and enhance its lipophilicity [514]. Ezetimibe (Zetia) is a pharmacological agent that is a selective inhibitor of cholesterol absorption. This compound was initially identified through a process known as library screening [515]. Naloxegol, commercially known as Movantik, is a derivative of naloxone that has been PEGylated to inhibit its ability to pass the blood–brain barrier [516].

DDSs composed of protein and peptide molecules confront challenges like enhancing physical stability, managing pharmacokinetic (PK) attributes like half-life and biodistribution, non-invasive application, overcoming biological barriers, minimizing immune reactions, and refining target precision. To address these challenges, commercial producers have adopted a range of strategies. For example, Desmopressin (DDAVP) was developed as a vasopressin analog, incorporating a non-standard amino acid to boost its stability and half-life [517]. Leuprolide acetate’s depot suspension, Lupron Depot, offers a prolonged-release microsphere formula of the luteinizing-hormone-releasing hormone, enhancing its half-life [518]. Insulin human inhalation powder, known as Afrezza, is an inhaled insulin variant comprising microparticles mixed with fumaryl diketopiperazine, ensuring it is apt for inhalation [519]. Semaglutide, or Rybelsus, is an orally administered GLP-1 agonist, blended with SNAC to enhance stomach absorption [520]. Pegademase bovine, or Adagen, is a PEGylated protein treatment designed to prolong half-life while decreasing immunogenic responses [521]. Belatacept, or Nulojix, is an innovatively designed fusion protein that showcases amino acid modifications to heighten its selectivity for CD86 and CD80 [522].

Antibody-based DDSs suffer from similar kinds of obstacles faced by protein- and peptide-based DDSs. Some examples of issues resolved by commercial producers are cited here. Certolizumab pegol, also known as Cimza, is the initial PEGylated antibody fragment to receive approval from the FDA [523]. This modification enhances the half-life of the antibody fragment and improves its solubility. Blinatumomab, commercially known as Blincyto, is a lyophilized antibody formulation that incorporates trehalose to enhance the stability of the antibody structure [524]. Trastuzumab and hyaluronidase-oysk (commercially known as Herceptin Hylecta) are a novel subcutaneous depot formulation that incorporates hyaluronidases to enable Herceptin’s controlled and prolonged release [525]. Panitumumab, commercially known as Vectibix, is the initial entirely human antibody to receive approval from the U.S. FDA [526]. This therapeutic agent effectively mitigates immunogenicity and inhibits the production of anti-antibodies.

Nucleic acid-based DDSs encounter various obstacles in their implementation, including the regulation of pharmacokinetic parameters, maintenance of stability, facilitation of efficient cell membrane penetration, attainment of access to the cytosol or nucleus after uptake, mitigation of immunogenic responses, and prevention of unwanted gene modifications. There are various commercial solutions available to address these concerns. Patisiran, also known as Onpattro, is a therapeutic agent that has received approval from the United States FDA [527]. This therapy utilizes small interfering RNA (siRNA) and lipid NPs to facilitate effective transportation to the liver and uptake by target cells. The therapy also utilizes ionizable cationic lipids to facilitate the drug’s release from endosomes following endocytosis. Fomivirsen, also known as Vitravene, represents the inaugural antisense oligonucleotide to receive approval from the FDA. It incorporates a modification in the form of a phosphorothioate backbone, which enhances its resilience against nucleases [528]. Givosiran, also known as Givlaari, is a GalNAc-siRNA compound that facilitates enhanced cellular absorption in liver hepatocytes [529]. Nusinersen, commercially known as Spinraza, has been granted approval for treating spinal muscular atrophy. This therapeutic intervention incorporates a modification known as 2’-O-methoxyethyl phosphorothioate, which serves the dual purpose of diminishing immunogenicity and enhancing stability [530]. CRISPR technology is utilized to modify CD34^+^ cells with CCR5 to combat HIV-1. The CD34^+^ cells, which are subject to clinical trials with the identifier NCT03164135, undergo ex vivo editing procedures. In order to detect any unintentional modifications, the process of whole-genome sequencing is employed subsequent to post-editing and engraftment [531].

Nucleic acid-based DDSs encounter challenges pertaining to the unpredictability of pharmacokinetic parameters, the need to sustain persistence and viability within the body, the imperative to minimize immune reactions, the preservation of therapeutic properties of cells, the assurance of precise delivery to the intended disease site, and the scalability of manufacturing processes. In light of these challenges, a number of novel ways have been devised. Preclinical alginate implants have been developed to regulate the release of chimeric antigen receptor (CAR) T cells to the specific illness site [532]. The SIG-001 treatment utilizes genetically engineered cells embedded in an antifibrotic matrix to achieve a prolonged therapeutic effect [533]. The administration of fludarabine conditioning chemotherapy is employed to mitigate the immunological rejection response toward infused chimeric antigen receptor (CAR) T cells [534]. Sipuleucel-T, an innovative immunotherapy utilizing dendritic cells that has received approval from the FDA, employs ex vivo antigen presentation to initiate and maintain a therapeutic cellular phenotype [535]. Matrix-induced autologous chondrocyte implantation (MACI) is a novel technique that effectively retains chondrocytes at the intended site [536], making it the initial cell-embedded scaffold product to receive approval from the FDA. Furthermore, Tisagenlecleucel, the first CAR T-cell therapy to receive approval from the FDA, established the standard for manufacturing autologous cell therapies [537].

## 10. Future Perspectives and Concluding Remarks

The ongoing progress in the domain of BINMs for micro/nanodevices in biomedical applications presents significant potential for transforming the healthcare sector and other related fields. The progression from the initial idea to the actualization of these materials has demonstrated their significant capacity to improve the performance of devices, achieve compatibility with biological systems, facilitate self-assembly processes, promote sustainability, and provide a wide range of applications. The utilization of the bio-inspired approach, which draws inspiration from nature’s efficiency and elegance, has not only facilitated the creation of innovative materials but has also provided a new lens through which to tackle intricate problems. Looking toward the future, the field of BINMs is anticipated to explore novel frontiers. The advancement of innovation can be propelled by incorporating various biological inspirations and elucidating complex structure–function relationships inherent in organisms.

Furthermore, the progress made in utilizing biomimetic materials and implementing energy-minimizing designs will facilitate the development of micro/nanodevices that are both highly efficient and environmentally sustainable. The potential applications within the micro/nanodevices domain are extensive and encompass various fields beyond biomedicine. The application of BINMs in various domains such as chemical reaction systems, energy harvesting and storage, environmental protection, sensors, agricultural sustainability, protective clothing, and adaptive materials demonstrates this approach’s wide-ranging capabilities and significant influence. These applications possess the capacity to transform industries and effectively tackle urgent global challenges fundamentally.

Although substantial advancements have been made thus far, there are still obstacles to fully overcome to harness the capabilities of BINMs for biomedical purposes. The successful resolution of challenges related to synthesis intricacies, attainment of accurate interfaces, mitigation of biocompatibility issues, assurance of long-term stability, and negotiation of regulatory and ethical considerations necessitates the collaborative endeavors of interdisciplinary groups. Incorporating knowledge from various disciplines such as biology, chemistry, material science, medicine, engineering, and other related fields will play a crucial role in determining the trajectory of BINMs in the future. BINMs signify a novel biomedical application era characterized by inventive designs, improved functionality, and diverse possibilities. The remarkable trajectory from inspiration to realization underscores the significance of biomimicry in propelling scientific progress. As scholars persist in investigating and enhancing these materials’ design principles, synthesis techniques, and applications, the range of potential outcomes will broaden, leading to a forthcoming era in which BINMs assume a crucial position in influencing our approach to healthcare, technology, and the global landscape.

## Figures and Tables

**Table 2:** A summary of all the subsections under this section and list of the most recent biomedical applications of micro/nanodevices fabricated from BINMs.

Sector	Devices	Bio-Inspiration	Mechanism	Applications	Refs.
Drug Delivery and Therapeutic Applications	Liposome-based drug delivery system	Liposomes	Loaded with chemotherapy drugs	Laryngeal cancer cells	[391]
	Liposome-based nanoarchitectonics	Liposomes	Loaded Ag NP	Cancer management	[392]
	Nano-liposome-based transdermal hydrogel		Targeted delivery of dexamethasone	Rheumatoid arthritis therapy	[393]
	Liposome-based nanocomposite drug delivery system		Loaded with Ag NPs, hyaluronic acid, lipid NPs	Cancer treatment	[394]
	Dendrimer-based nanocomposites	Dendrimers	RNA delivery	Cancer vaccination	[395]
	Dendrimernanosystems	Dendrimer nanomicelles	Adaptive tumor-assisted drug delivery via extracellular vesicle hijacking	Tumor treatment	[396]
	Lipid-coated ruthenium dendrimer conjugation	Dendrimers	Hydrophobic locking protocol	Cancer treatment	[397]
	Dendrimer-gel-derived drug delivery systems		Encapsulation or chemical coupling	Glaucoma medications	[398]
	Erlotinib-loaded dendrimer nanocomposites		Entrapment or encapsulation of drug	Targeted lung cancer chemotherapy	[399]
	Antiglycolytic cancer treatment	Micelles	Considering the unique metabolism of cancer cells	Antiglycolytic cancer treatment	[400]
	Core-(shell-cross-linking)-corona micelles (CSCCMs)		Shell protection strategy	Photo- and pH dual-sensitive drug delivery	[401]
	Polymeric nanocomposite		Polymeric micelles using citraconic amide bonds	Cancer treatment	[402]
	Hyaluronic acid-coated polymeric micelles		Through specific cellular uptake	Liver fibrosis therapy	[403]
	Membrane-coated nanosystems	Blood cells Cancer cells Stem cells Extracellular vesicles Viral capsids Bacteria	Targeting Diagnosis Drug delivery	Theranostic applications	[404]
	Surface plasmon resonance (SPR)-based sensors	Cancer cells	Detected via a change in SPR angle	Detection of cancer	[405]
	Metal–organic framework–azidosugar complex	Cancer cell membrane	Metabolic glycan labeling (MGL)	Breast cancer treatment	[406]
	Gene delivery system	Viral vectors	Nucleic acid molecule in a protein coat	Gene therapy and imaging	[407,408]
		Virus-like particles	Self-assembled capsules composed of viral capsid or envelope proteins that preserve antigenicity	Vaccine, drug, and gene delivery	[409]
		Virosomes	Virion-like phospholipid bilayer vesicle containing an incorporated glycoprotein within an empty compartment	Vaccine, gene, and drug delivery	[410,411,412]
	Enzyme-powered nanomotors	Natural molecular motor	Self-propulsion	Protein delivery and imaging	[413]
	Micro/nanomotors	Natural mobile microorganisms	Self-propulsion	Diagnostics, therapeutics, and theranostics	[414]
		Sperm cells	Propulsion of its own flagellum Directional guidance by chemotaxis, thermotaxis, and rheotaxis	Diagnostics, therapeutics, and theranostics	[415,416,417,418]
		Bacteria	Self-propulsion and driven by stimuli	Diagnostics, therapeutics, and theranostics	[419,420,421]
		Algae	Electro-magnetic field propulsion and driven by stimuli	Diagnostics, therapeutics, and theranostics	[422,423,424]
Biomimetic Nanobiosensors	Biomimetic nanophotonic biosensors	Nature’s photonic crystal	Natural structural color and stimuli-responsive photochemical reaction	Enzyme detection, detection of spiked human serum	[425,426,427]
		Structural colors in chitin-constituted insect shells	Hierarchical structures of carbohydrate nanofibrils such as chitin and cellulose	Nanobiosensor label-free detection of urinary venous thromboembolism biomarker	[428,429,430]
	Metallic nanobiosensors	Bio-inspired metallic NPs	Fluorescence signal variations that depend on the size, color, and surroundings	Electrochemical, colorimetric, and fluorescence nanobiosensors	[431,432,433,434,435,436,437]
	Polymer-composite-based nanobiosensors	Nature’s biorecognition components such as antibody, enzyme, antigen, protein, DNA, etc.	Detection of biological reactions and conversion to signals	Detection of interleukin-8 (IL-8), TNF-α, cancer biomarkers, and neuron-specific enolase (NSE)	[438,439,440,441,442]
	Hydrogel-based piezoelectric sensors	Biological tissues and organisms that exhibit piezoelectric properties	Due to the asymmetric arrangement of atoms or molecules in the crystal structure of the material	Wound healing, ultrasound simulation, and imaging	[443,444,445]
Organ-on-Chip	Single organ-on-chip	Different human organs individually such as liver, kidney, lung, gut, heart, muscle, blood–brain barrier, seplon, bone marrow, etc.	Through the replication and simulation of the physiological functions and interactions of human organs in a microfluidic platform	Drug development and testing, disease modeling, personalized medicine, toxicity screening, and reducing the reliance on animal testing in pharmaceutical research	[363,446,447,448,449,450,451]
	Multiple organs-on-chip	Multiple organs are interconnected similar to the human body	Replicating the physiological and biochemical characteristics of organs in a controlled and interconnected manner	Enabling the study of organ–organ crosstalk, drug responses, and disease progression with higher accuracy and relevance compared to traditional in vitro models	[369,370,452]
	Human-on-chip	Whole human body	Replicating the complexity and functionality of the entire human body in a miniature, interconnected platform	Applicable for accurate and predictive pre-clinical studies, drug testing, and disease modeling	[371,453,454]
	Patients-on-chip	Patient-specific cells, tissues, or induced pluripotent stem cells (iPSCs)	Replicating the unique characteristics of an individual’s biology, including their genetic background, disease conditions, and drug response, in a microscale platform	Particularly valuable for precision medicine, where tailored therapies are developed based on a patient’s specific needs and response	[455,456]
Cancer-on-Chip	3D breast COC	Breast tumors	Replicating key aspects of the breast tumor microenvironment, such as the extracellular matrix composition, stiffness, and architecture	Therapeutic evaluation of drug delivery systems	[457,458,459]
	Pancreatic COC	Pancreatic tumors	The use of microfluidic technology to recreate the microenvironment of pancreatic tumors	Platform that recapitulates the tumor microenvironment and enables detailed investigations into pancreatic cancer biology, drug responses, and potential therapeutic strategies	[456,460,461]
	Lung COC	Lung tumors	Platform that replicates the key features of lung tumors and their microenvironment	Provides a potent tool for studying the biology of lung cancer and creating tailored treatments for this terrible illness by simulating the lung tumor microenvironment, including mechanical forces, oxygen levels, and cellular interactions	[462,463,464]
Wound Healing Dressing Mat	Silk-fibroin-based wound dressings	Silkworm cocoons	Creating a favorable microenvironment for wound healing and tissue regeneration	Wound healing, tissue regeneration, bioactive molecule delivery, cosmetic uses, drug delivery, hemostatic dressings, and tissue engineering scaffolds	[465,466,467]
	Biopolymer nanocomposite thin film	Natural ECM	Creating a flexible and biocompatible film that can adhere to the wound, maintain a moist environment, and release bioactive molecules to promote wound healing and tissue regeneration	Wound healing and tissue regeneration	[468,469,470]
	Bio-inspired adhesive formulations	Gecko feet, mussel adhesive proteins, or insect adhesives	Mimicking the chemical and physical properties of natural adhesives	Medical adhesives for wound closure and healing	[471,472,473]
Bio-inspired Antimicrobial Surface	Structure-oriented antimicrobial surface	Natural surface morphology of plants, animals, and insects	Affecting microbial adhesion, internal cell structures, and cell migration	Antibacterial, antiviral, and antifungal applications	[474,475,476,477]
	Peptide-based surface	Naturally occurring peptide molecules found in various organisms	By specifically interacting with the negatively charged membranes of bacteria, fungi, and viruses, AMPs produce their antimicrobial actions by inducing holes to develop and ultimately cell death	Antimicrobial coating, wound dressing, detection of pathogens, nanomedicine, and medical implants	[478,479,480,481]
	Metal/metal oxide NP-based antimicrobial surface	Natural metallic NPs	Antimicrobial properties of metal/metal oxide NPs	Antimicrobial coating, wound dressing, detection of pathogens, nanomedicine, and medical implants	[385,482,483]
	Chitosan-based antimicrobial surfaces	Chitosan	Interacting with bacterial cell membranes, disrupting their structure, and leading to cell death	Antimicrobial coating, wound dressing, detection of pathogens, nanomedicine, and medical implants	[484,485,486]
	Mussel-inspired antimicrobial coatings	Adhesive properties of mussel foot proteins	The catechol groups facilitate strong and stable interactions with surfaces, providing long-lasting antimicrobial properties	Coating medical devices, implants, and wound dressings	[487,488,489]
	Bacteriophage-based antimicrobial surface	Naturally occurring viruses that specifically target and infect bacteria	These surfaces provide a promising substitute for conventional antibiotics by using bacteriophage selectivity to eradicate particular bacterial infections	Wound dressings, medical implants, and catheters	[490,491,492]

## Data Availability

Data is contained within the article.
